# Selectfluor-Promoted Chemoselective Self-Etherification, Oxidation, and Ritter-Type Amidation of Benzhydrols

**DOI:** 10.1021/acs.joc.6c00350

**Published:** 2026-05-04

**Authors:** Muhammed Hanifi Çelikoğlu, Büşra Odabaş, Arif Daştan, Sefa Uçar, Bilal Nişancı

**Affiliations:** Department of Chemistry, Faculty of Sciences, 37503 Ataturk University, Erzurum 25240, Turkey

## Abstract

The role of in situ generated fluorinated sulfur intermediates, formed through the interaction of the electrophilic fluorine source Selectfluor with sulfur-containing reagents, in the chemoselective transformations of benzhydrols was investigated from a mechanistic perspective. The 1,2-dimethyldisulfide–Selectfluor combination enabled the selective self-etherification of benzhydrols, and this transformation was shown to proceed via benzhydrol-centered reactive intermediates activated by fluorinated sulfur species. Upon increasing the reaction temperature and 1,2-dimethyldisulfide was replaced with carbon disulfide, the reaction pathway was altered, leading to the selective oxidation of benzhydrols to the corresponding benzophenones. Under the same conditions, when nitromethane was employed as the solvent, Ritter-type amidation was achieved via the solvolytic activation of the benzhydrols. Based on control experiments and the effects of reaction parameters, plausible mechanisms for these condition-dependent chemoselective transformations are proposed and discussed.

## Graphical abstract

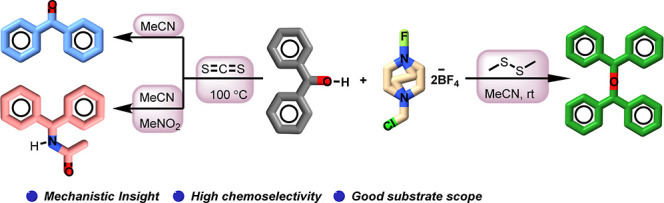

## Introduction

Selectfluor, a strong “N–F”-type electrophilic fluorinating reagent and oxidizing agent, has been extensively employed in the functionalization of organic molecules as its high reactivity allows the formation of key reactive intermediates under mild conditions, together with its safety, low toxicity, and ease of handling.^1^ It is well-known that sulfides rapidly react with Selectfluor to form fluorosulfonium salts.^2^ Similarly, the sulfur atoms in disulfides readily interact with the electrophilic fluorine provided by Selectfluor, leading to the formation of sulfur radicals^3^ or nucleophilically susceptible cationic sulfur centers^4^ as dictated by the reaction conditions. It has been reported that the reactions of aromatic and benzylic disulfides with Selectfluor in aqueous media result in the formation of thiosulfonates, whereas increasing the Selectfluor loading and elevating the reaction temperature lead to the formation of sulfonyl fluorides (Scheme 1a).^5^ The reaction of an aryl disulfide–Selectfluor combination with primary, secondary, and tertiary aliphatic alcohols has been shown to result in the formation of sulfinic esters via sulfination of the alcohols (Scheme 1b).^6^ Sulfinamides have been reported to be obtained in moderate yields from the reaction of the same combination with amines (Scheme 1c).^7^ In our previous study, we achieved the deoxygenation of *N*-heterocyclic *N*-oxides by employing Selectfluor in combination with disulfides (Scheme 1d).^8^ In all of the aforementioned studies, the reaction mechanisms are observed to be initiated by the transfer of fluorine from Selectfluor to the sulfur atom of the disulfide. In addition to the experience gained from deoxygenation and our other previous studies on Selectfluor and sulfide chemistry,^9^ the fact that benzhydrol derivatives provide mechanistically suitable models due to their sensitivity to carbocationic and radical processes motivated us to investigate the reactions of the reactive intermediates generated by the Selectfluor–sulfide combination with these compounds. In our initial experiments, we observed evidence for the formation of a Ritter-type amide arising from the involvement of acetonitrile in the reaction alongside benzhydrol ether and the oxidation product benzophenone. By validating our hypothesis that reactive intermediates bearing a fluorinated sulfur unit can be generated from a Selectfluor–carbon disulfide combination, and by leveraging the benefits of elevated temperature and judicious solvent selection, we uncovered highly chemoselective and unprecedented strategies for the selective self-etherification, oxidation, and Ritter-type amidation of benzhydrols (Scheme 1e) (see Table 1).

**Scheme 1:**
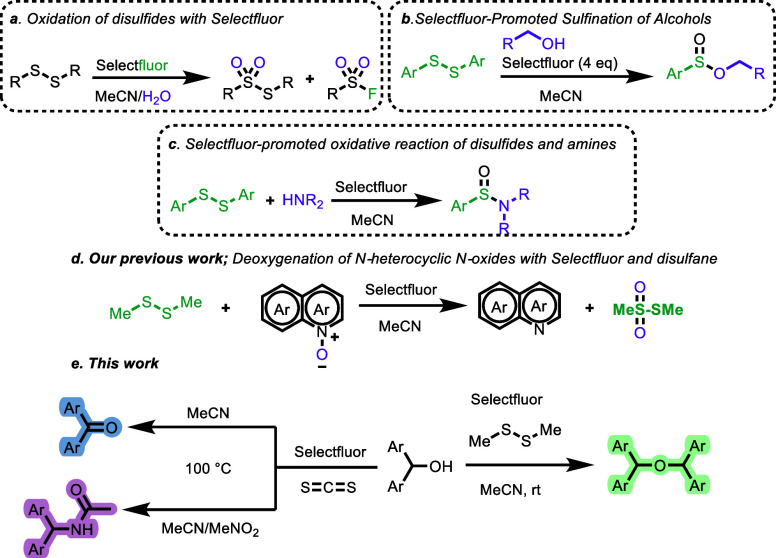
Representative Examples Demonstrating the Reactivity of Selectfluor–Disulfide Mixtures

**Table 1:**
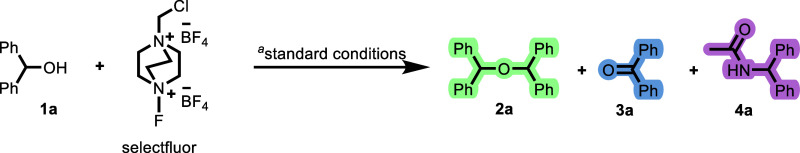
Optimization Study

entry	Selectfluor	additive	solvent	time (h)	temperature	^a^ ^,^ ^b^yield (%)		
						**2a**	**3a**	**4a**
1	2.0 eq	1,2-dimethyldisulfane (2 equiv)	MeCN	17	rt	85	trace	trace
2	2.0 eq	dimethylsulfane (2 equiv)	MeCN	17	rt	44	14	trace
3	2.0 eq	carbon disulfide (2 equiv)	MeCN	17	rt	45	32	trace
4	2.0 eq	1,2-dimethyldisulfane (1 equiv)	MeCN	17	rt	86	trace	trace
5	1.0 eq	1,2-dimethyldisulfane (1 equiv)	MeCN	17	rt	93	-	-
6	1.0 eq	1,2-dimethyldisulfane (0.5 equiv)	MeCN	17	rt	93	-	-
***7***	***0.55 eq***	***1,2-dimethyldisulfane*** ***(0.55 equiv)***	***MeCN***	**17**	***rt***	***94***	***-***	***-***
8	2.0 eq	carbon disulfide (2 equiv)	MeCN	18	60 °C	20	50	25
9	2.0 eq	carbon disulfide (2 equiv)	MeCN	18	80 °C	-	61	35
10	2.0 eq	carbon disulfide (2 equiv)	MeCN	18	100 °C	-	81	trace
11	2.0 eq	carbon disulfide (2 equiv)	CH_3_NO_2_	18	100 °C	-	45	-
12	2.0 eq	carbon disulfide (2 equiv)	HFIP	18	100 °C	-	43	-
13	1.0 eq	carbon disulfide (2 equiv)	MeCN	18	100 °C	-	82	trace
14	2.0 eq	carbon disulfide (1 equiv)	MeCN	18	100 °C	-	82	trace
15	2.0 eq	carbon disulfide (0.5 equiv)	MeCN	18	100 °C	-	94	trace
***16***	***1.0 eq***	***carbon disulfide (0.5 equiv)***	***MeCN***	***18***	***100 °C***	***-***	***94***	***-***
17	1.0 eq	carbon disulfide (0.5 equiv)	MeCN/MeNO_2_ (1:4)	17	60 °C	43	trace	45
18	1.0 eq	carbon disulfide (0.5 equiv)	MeCN/MeNO_2_ (1:4)	17	100 °C	-	52	44
19	1.0 eq	carbon disulfide (0.5 equiv)	MeCN/MeNO_2_ (1:1)	17	100 °C	-	50	46
20	1.0 eq	carbon disulfide (0.5 equiv)	MeCN/MeNO_2_ (4:1)	17	100 °C	-	trace	71
21	1.0 eq	carbon disulfide (0.5 equiv)	MeCN/HFIP (4:1)	17	100 °C	-	trace	68
22	1.0 eq	carbon disulfide (0.5 equiv)	MeCN/DMF (4:1)	17	100 °C	27	15	12
23	1.0 eq	carbon disulfide (0.5 equiv)	MeCN/DMSO (4:1)	17	100 °C	50	trace	36
24	0.5 eq	carbon disulfide (0.5 equiv)	MeCN/MeNO_2_ (4:1)	17	100 °C	-	52	49
25	0.25 eq	carbon disulfide (0.25 equiv)	MeCN/MeNO_2_ (4:1)	17	100 °C	-	trace	72
**26**	**0.25 eq**	**carbon disulfide** **(1 equiv)**	**MeCN/MeNO**_**2**_ **(4:1)**	**17**	**100 °C**	**-**	**trace**	**93**

a Standart conditions: **1a** (0.4 mmol), solvent (5 mL), 17–18 h (determined by TLC).

b Determined by ^1^H NMR based on **1a**.

In the literature, the etherification, Ritter-type amidation, and oxidation of benzhydrols have been investigated separately using strong Brønsted and Lewis acids, metal salts, transition-metal complexes, heterogeneous catalysts, and various oxidative systems. Benzhydrol ethers are typically synthesized under metal-mediated or strongly acidic conditions employing ZnCl_2_, TiF_4_, and Fe­(OTf)_3_, or in the presence of specialized transition-metal catalysts such as (^Ph^_2_NNN)­RuCl_2_(CO),^10^ whereas Ritter-type amidation reactions generally require superacids or high-temperature nitrile-containing media.^11^ In contrast, the oxidation of benzhydrols has been achieved using SO_2_F_2_, peroxides, P_2_O_5_-based systems, or costly and specialized heteropolyacid catalysts such as H_6_P_2_W_18_O_62_.^12^ However, the chemoselective differentiation of these three transformations and their rational control within a single reactive platform have not yet been addressed in a general and practical manner.

## Results an Discussion

Owing to its low cost, facile removal from the reaction mixture after completion, and the good reactivity demonstrated in our previous study,^8^ 1,2-dimethyldisulfane was selected and used in combination with Selectfluor to react with diphenylmethanol in acetonitrile at room temperature. Under these conditions, we unexpectedly discovered the formation of (oxybis­(methanetriyl))­tetrabenzene (**2a**) along with trace amounts of benzophenone (**3a**) and *N*-benzhydrylacetamide (**4a**) (entry 1). In light of this result, we focused on the chemoselective synthesis of these three products (**2a**, **3a**, and **4a**). First, we changed the sulfide reagent and observed a pronounced increase in the proportion of the oxidation product (**3a**), particularly when carbon disulfide was employed (entries 2 and 3). Meanwhile, the equivalent amounts of Selectfluor and 1,2-dimethyldisulfane used in the synthesis of the ether (**2a**) were optimized (entries 4–7); in the reaction carried out in acetonitrile at room temperature, the use of 0.55 equiv of Selectfluor and 0.55 equiv of 1,2-dimethyldisulfane was identified as the optimal conditions for ether synthesis (entry 7). Subsequently, we focused on the chemoselective synthesis of the oxidation and Ritter products (**3a** and **4a**), and we tested the carbon disulfide-containing reactions, which were found to be more prone to oxidation product (**3a**) formation (Entry 3), at 60, 80, and 100 °C, respectively (entries 8–10). Further, by optimizing the reaction solvent and the equivalent amounts of Selectfluor and carbon disulfide (entries 11–16), we determined that the optimal conditions for the synthesis of **3a** involved the use of 1 equiv of Selectfluor and 0.5 equiv of carbon disulfide in acetonitrile at 100 °C (entry 16). We hypothesized that the Ritter product (**4a**) is formed via a diphenylmethylium carbocation and considered that the addition of polar solvents to the reaction, together with acetonitrile, could enhance the yield of the amidation products (**4a**). In line with this hypothesis, the effect of adding various polar solvents to acetonitrile was examined in carbon-disulfide-containing systems (entries 17–26). MeCN/MeNO_2_ mixtures preferentially promoted the formation of the Ritter product (**4a**), while both the solvent ratio and reagent equivalents exerted a pronounced influence on chemoselectivity. At lower Selectfluor and higher carbon disulfide loadings, the amidation product (**4a**) became dominant, and the highest yield of **4a** was achieved in a MeCN/MeNO_2_ (4:1) medium using 0.25 equiv of Selectfluor and 1 equiv of carbon disulfide (entry 26).

Under the optimized conditions, we investigated the functional group tolerance and substrate scope of the self-etherification, oxidation, and Ritter-type amidation of benzhydrols. As shown in Scheme 2, in addition to benzhydrol derivatives bearing electron-donating or electron-withdrawing substituents at the ortho, meta, or para positions, the reactivity of di­(thiophen-2-yl)­methanol, di­(furan-2-yl)­methanol, 10,11-dihydro-5*H*-dibenzo­[*a,d*]­[7]­annulen-5-ol, and 5*H*-dibenzo­[*a,d*]­[7]­annulen-5-ol toward self-etherification, oxidation, and Ritter-type amidation reactions was systematically investigated. Symmetric and asymmetric benzhydrol derivatives bearing electron-donating or -withdrawing substituents at the ortho, meta, or para positions were generally found to undergo all three transformations in good to high yields. In particular, para-substituted benzhydrols were well tolerated across all reaction manifolds, whereas strongly electron-donating methoxy groups led to a pronounced decrease in reactivity in certain cases (Scheme 2). In the self-etherification reaction, a majority of benzhydrol derivatives bearing substituents with diverse electronic properties at the para position were efficiently converted into the corresponding ether products in high yields. In contrast, benzhydrols substituted at the ortho or meta positions were found to be unreactive under these conditions. Heteroaromatic substrates such as di­(thiophen-2-yl)­methanol and di­(furan-2-yl)­methanol were not completely inert; however, they preferentially led to the formation of nonetheric, polymeric-type products rather than the desired ether derivatives. The etherification reactions of methyl 4-(hydroxy­(*p*-tolyl)­methyl)­benzoate and (4-nitrophenyl)­(phenyl)­methanol proceeded in low yields at room temperature, whereas a marked improvement in the etherification efficiency was observed upon increasing the reaction temperature to 80 °C. Nevertheless, as a side effect of the elevated temperature, the amidation product **4g** was obtained as a side product during the synthesis of ether **2g** under these conditions. Substrates with extended π-conjugation, namely, 10,11-dihydro-5*H*-dibenzo­[a,d]­[7]­annulen-5-ol and 5*H*-dibenzo­[a,d]­[7]­annulen-5-ol, underwent a smooth self-etherification process, enabling the selective formation of products containing either single or double bonds. However, under prolonged reaction conditions, the initially formed ether products (**2r** and **2s**) derived from these substrates underwent subsequent disproportionation^13^ to afford the corresponding ketone and diarylmethane, and therefore the reaction times were deliberately kept short. With respect to oxidation, the benzhydrol core exhibited broad functional group tolerance, and a wide range of substituted benzhydrols, as well as 10,11-dihydro-5*H*-dibenzo­[a,d]­[7]­annulen-5-ol, were cleanly converted into the corresponding ketones in high yields. Electron-withdrawing substituents were generally found to accelerate the oxidation process, whereas the presence of a sterically demanding substituent at the ortho position completely suppressed the reactivity under these conditions. Benzhydrol derivatives bearing heteroaromatic substituents were found to be incompatible with the oxidation conditions. Notably, oxidation of 5*H*-dibenzo­[a,d]­[7]­annulen-5-ol resulted in the formation of anthracene and anthracene-9-carbaldehyde (see Supporting Information S16 and S17). Under the optimized conditions, the formation of amidation products was occasionally observed alongside the desired oxidation products. To suppress this side reaction, the amounts of Selectfluor and carbon disulfide were increased, which led to a significant reduction in amidation byproduct formation. Nevertheless, trace amounts of amidation products were still detected in the synthesis of certain benzophenone derivatives. In particular, benzhydrol derivatives bearing electron-donating methyl or methoxy substituents (**3e**, **3f**, and **3l**) predominantly underwent amidation, with the corresponding amide products becoming the major species. Under Ritter-type amidation conditions, benzhydrols bearing aromatic rings with diverse electronic properties were found to participate in amide formation with high selectivity. In contrast, substrates containing heteroaromatic rings were not completely inert; instead, they led to the formation of complex product mixtures that could not be isolated rather than the corresponding amidation products. Notably, benzhydrol derivatives substituted at the ortho or meta positions, which were unreactive in the self-etherification and oxidation reactions, were fully compatible with amidation conditions. With respect to annulen derivatives, the outcome of the amidation reaction exhibited pronounced selectivity, depending on the degree of bond saturation. Moreover, during the synthesis of certain amidation products, oxidation products were also detected as minor byproducts in low yields. Overall, these results demonstrate that the Selectfluor-based system provides broad substrate scope and high chemoselectivity for benzhydrol derivatives while highlighting heteroaromatic frameworks and strongly electron-donating substituents as limiting factors for reactivity.

**Scheme 2:**
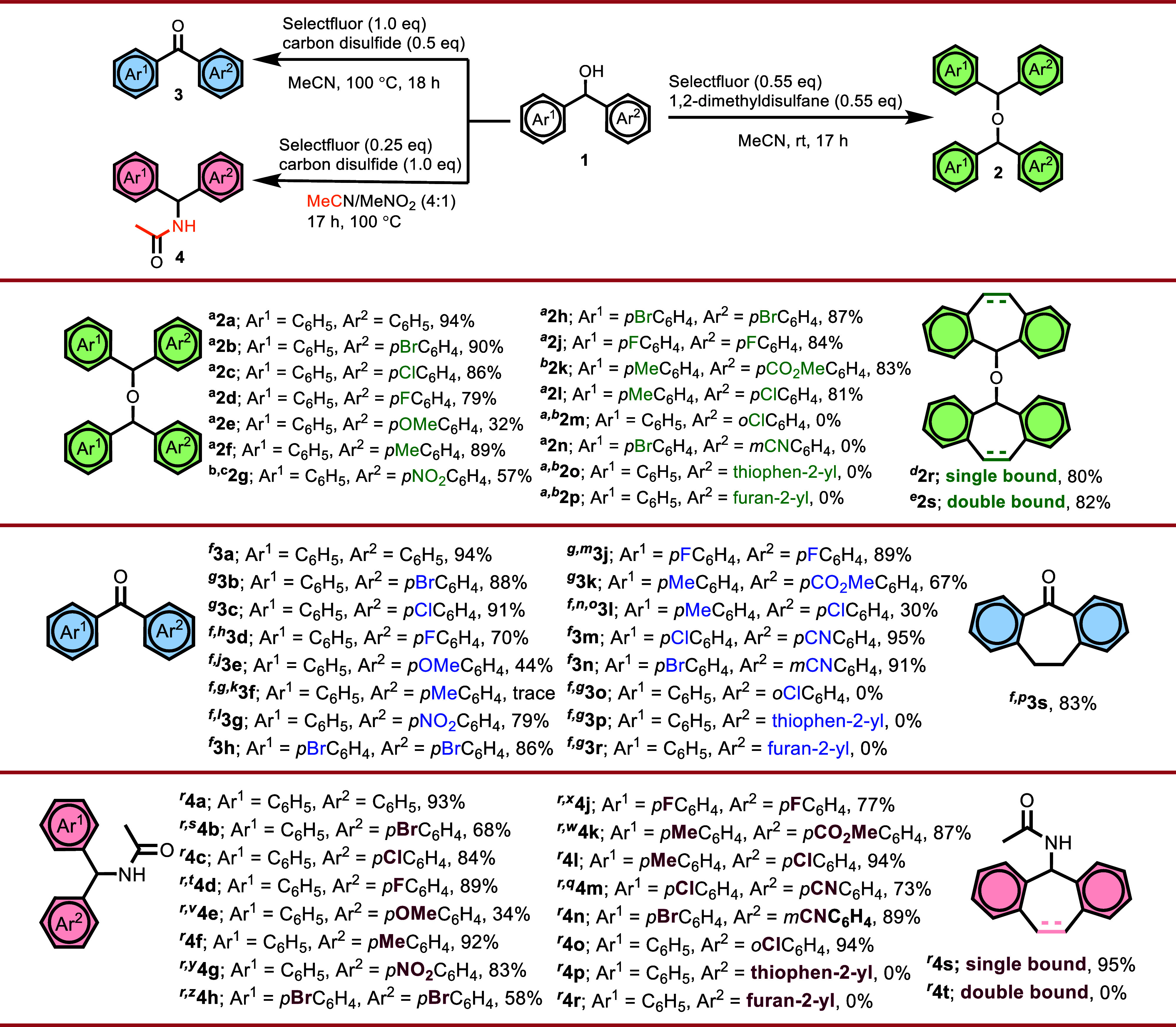
Substrate Scope

a Reaction conditions: **1** (0.4 mmol), Selectfluor (0.22 mmol), 1,2-dimethyldisulfane (0.22 mmol), MeCN (5 mL), rt, 17 h,
b Reactions were carried out at 80 °C.
c
**4g** was obtained as a side product (32%).
d Reaction time was 6 h.
e Reaction time was 1 h.
f
**1** (0.4 mmol), Selectfluor (0.4 mmol), carbon disulfide (0.2 mmol), MeCN (5 mL), 100 °C, 18 h.
g
**1** (0.4 mmol), Selectfluor (1.2 mmol), carbon disulfide (0.6 mmol).
h
**4d** was obtained as a side product (26%).
j
**4e** was obtained as a side product (40%).
k
**4f** was the main product, **l4g** was obtained as a side product (19%).
m
**4j** was obtained as a side product (trace).
n Reactions were carried out at 110 °C.
o
**4l** was obtained as the main product (62%).
p
**4s** was obtained as a side product (trace).
r
**1** (0.4 mmol), Selectfluor (0.1 mmol), carbon disulfide (0.4 mmol), MeCN/MeNO_2_ (5 mL, 4:1), 100 °C, 17 h.
s
**3b** was obtained as a side product (23%).
t
**3d** was obtained as a side product (trace).
v Reaction time was 14 h.
y
**3g** was obtained as a side product (trace).
z
**3h** was obtained as a side product (40%).
x
**3j** was obtained as a side product (15%).
w
**3k** was obtained as a side product (8%).
q
**3m** was obtained as a side product (20%), yields of isolated products are given.

The mechanistic studies presented in Scheme 3 clearly demonstrate that the chemoselective transformations of benzhydrol derivatives observed under the Selectfluor–disulfide/CS_2_ system proceed through well-defined and distinguishable reactive intermediates. Initial control experiments (Scheme 3, a,d) revealed that benzhydrol does not undergo etherification, oxidation, or Ritter-type amidation in the presence of Selectfluor, carbon disulfide, or 1,2-dimethyldisulfane alone, even under conditions employing increased equivalents and elevated temperatures. These results indicate that the reaction is not driven by any single reagent individually; rather, a synergistic interaction between Selectfluor and the sulfur-containing components is essential for the reaction to occur. According to the proposed mechanism, in the initial stage Selectfluor interacts with 1,2-dimethyldisulfane or carbon disulfide, leading to the formation of an electrophilically activated sulfur species with cationic character (Scheme 3g,h). This interaction is directly supported by the ^1^H NMR spectra obtained from the control experiments shown in Scheme 4.

**Scheme 3:**
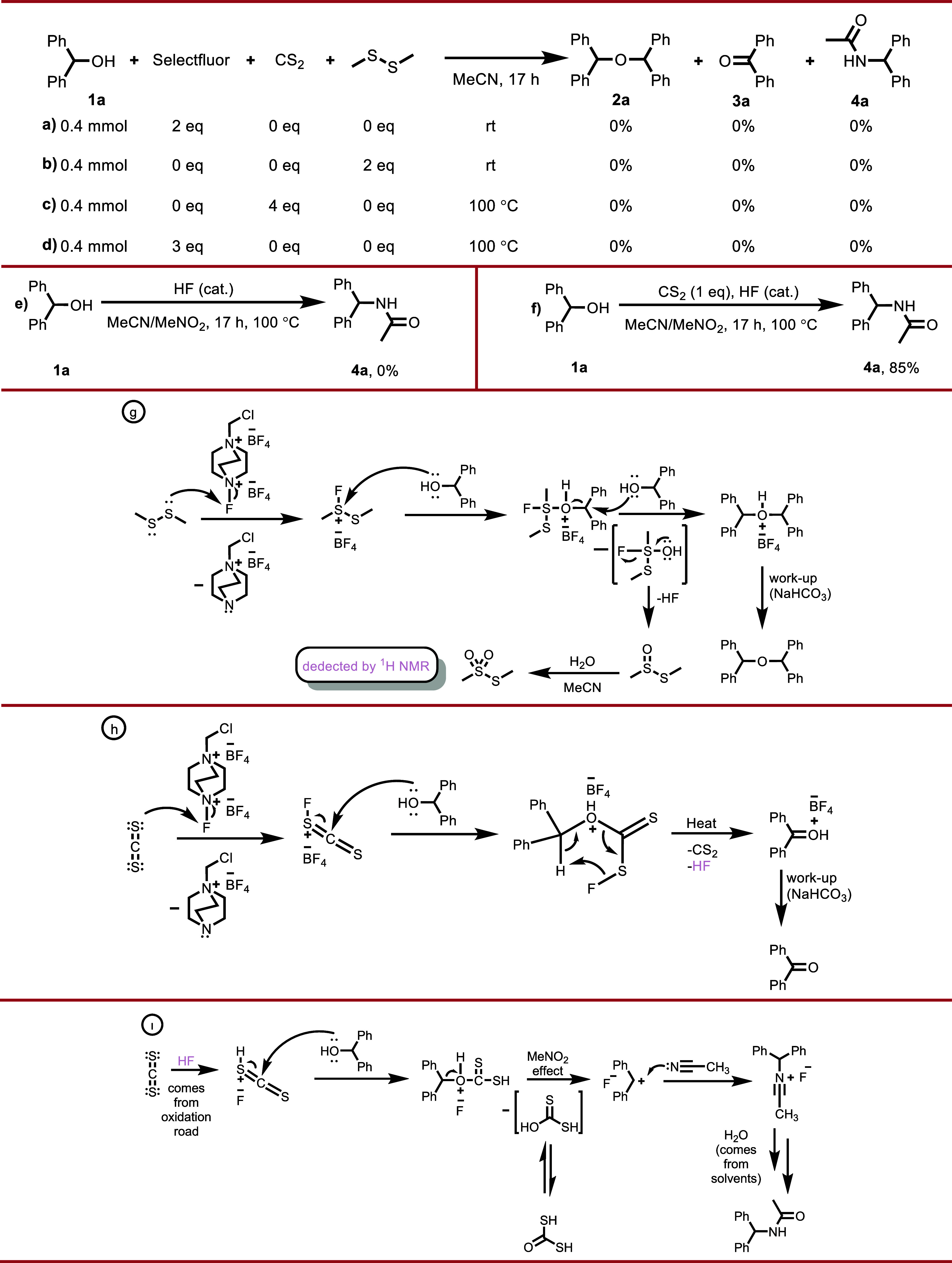
Mechanistic Studies and Proposed Mechanisms

**Scheme 4:**
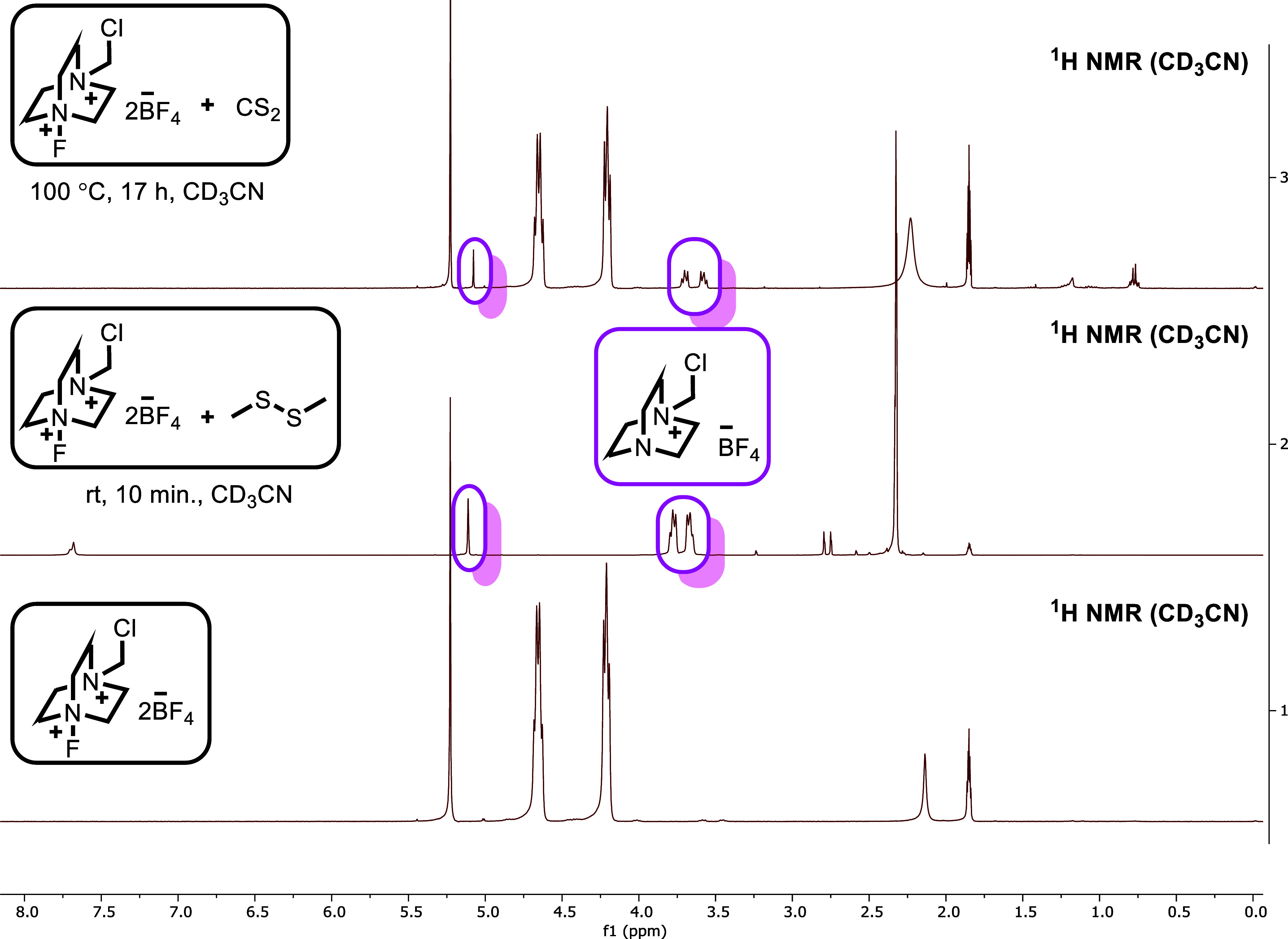
^1^H NMR Spectra of Control Experiments

Indeed, when Selectfluor was mixed separately with 1,2-dimethyldisulfane or CS_2_, the resulting spectra in both cases revealed the loss of the electrophilic fluorine atom from Selectfluor and the formation of the (chloromethyl)­DABCO species. These observations strongly indicate that the fluorine atom is accepted by either CS_2_ or 1,2-dimethyldisulfane, and that the generation of the active sulfur species occurs at this stage. Although the fluorination of the sulfur atom in 1,2-dimethyldisulfane by Selectfluor has been mechanistically proposed in several studies,^4^ no direct experimental evidence supporting this assumption has been explicitly reported in the literature. Moreover, to the best of our knowledge, the interaction between Selectfluor and carbon disulfide as a trigger for reactivity is disclosed here for the first time. The resulting reactive sulfur intermediate subsequently interacts with the hydroxyl group of benzhydrol, leading to the formation of the corresponding intermediate species (Scheme 3g,h). This step constitutes a common entry point for both the etherification and oxidation pathways. Under disulfide-containing conditions, this intermediate is trapped by the nucleophilic attack of a second benzhydrol molecule, resulting in the formation of the symmetric benzhydrol ether (Scheme 3g). In contrast, in the presence of carbon disulfide, the elimination of HF and CS_2_ from the intermediate facilitated by elevated temperature leads to the formation of benzophenone (Scheme 3, h).

According to the proposed mechanism, the formation of *S*-methylmethanesulfinothioate is expected during the etherification process (Scheme 3, g). It has been reported in the literature^5^ that such species undergo further oxidation under Selectfluor/MeCN conditions to afford *S*-methylmethanesulfonothioate. In the present study, the detection of the methanesulfonothioate species in the crude ^1^H NMR spectrum of benzhydrol’s etherification reaction shown in Supporting Information 11 provides compelling experimental evidence in strong support of the proposed etherification mechanism (Scheme 3h). On the other hand, under CS_2_-containing conditions, it is proposed that a carbonodithioate-type complex is formed via the active sulfur species, assisted by the HF released during the oxidation process (Scheme 3g). Subsequent generation of the diphenylmethylium cation from this intermediate, followed by its reaction with acetonitrile, provides a mechanistically consistent pathway toward Ritter-type amidation (Scheme 3, (i). The formation of HF along this pathway and the isolation of the amide product after aqueous workup are fully consistent with the experimental observations. Indeed, a control experiment (Scheme 3, e) designed to probe the role of HF demonstrated that a catalytic amount of HF alone in a MeCN/MeNO_2_ medium is insufficient to initiate amide formation. In contrast, when CS_2_ was added under otherwise identical conditions (Scheme 3, f), the Ritter-type amide product was obtained in a high yield (85%). Beyond these control experiments, several observations from the substrate scope (Scheme 2) further support the proposed mechanism. The formation of trace amounts of oxidation products under the amidation conditions in most cases suggests that the oxidation process is required for the release of catalytic amounts of HF. Moreover, the fact that (2-chlorophenyl)­(phenyl)­methanol, which does not undergo etherification or oxidation nevertheless affords the amide product in high yields, strongly argues against an S_N_2-type displacement pathway and instead supports the involvement of a carbocationic intermediate. In this context, although the *ortho*-chloro substituent imposes significant steric hindrance that disfavors S_N_2 processes, the corresponding (2-chlorophenyl)­(phenyl)­methylium carbocation remains theoretically well suited for Ritter-type amidation. Similarly, the preferential formation of **4s** in high yields, accompanied by the absence of **4t** (Scheme 2), can be rationalized by the relative ease of formation of the 10,11-dihydro-5*H*-dibenzo­[a,d]­[7]­annulen-5-ylium carbocation compared to the more conformationally constrained and planar 5*H*-dibenzo­[a,d]­[7]­annulen-5-ylium carbocation. Collectively, these findings provide strong additional support for the proposed carbocation-mediated mechanistic pathway. In conclusion, when the control experiments (Scheme 3, a–f), product distributions (Scheme 2), and spectroscopic findings (Scheme 4 and Supporting Information 11) are considered collectively, it becomes evident that both the reaction pathway and the observed chemoselectivity are governed by the nature of the reactive sulfur species and the composition of the reaction medium. These results lead to the conclusion that Selectfluor functions in this system not merely as a fluorinating agent but as a key activator that triggers the formation of critical reactive intermediates.

## Conclusion

In summary, a Selectfluor-promoted strategy enabling highly chemoselective self-etherification, oxidation, and Ritter-type amidation of benzhydrols has been developed. The three divergent transformations proceed with excellent chemoselectivity under closely related conditions, allowing controlled access to distinct products from common substrates. Mechanistic studies, supported by control experiments and spectroscopic data, suggest that these reactions operate through related reactive intermediates whose reactivities are dictated by the reaction environment. The protocol exhibits a broad substrate scope and good functional group tolerance, and its synthetic practicality is demonstrated by gram-scale reactions for all three transformations (SI10–SI11). Collectively, this work highlights the versatility of Selectfluor in chemoselective benzhydrol functionalization and provides mechanistic insight into its role in promoting divergent reactivity.

## Supplementary Material



## Data Availability

The data underlying this study are available in the published article and the Supporting Information.

## Notes

General experimental details, general procedures, characterization for all products, scale-up experiments, control experiments, and copies of ^1^H and ^13^C­{^1^H} spectra (PDF)
