# Supplementary material for: Selectfluor-Promoted Chemoselective Self-Etherification, Oxidation, and Ritter-Type Amidation of Benzhydrols
Source: J Org Chem. 2026 May 4;91(19):6687–94. doi: 10.1021/acs.joc.6c00350 (PMC13185097; doi:10.1021/acs.joc.6c00350)
Supplement: Supplementary file 1 [file jo6c00350_si_001.pdf]

Supporting Information

Selectfluor-Promoted Chemoselective Self-Etherification, Oxidation, and Ritter-Type Amidation of Benzhydrols

Muhammed Hanifi Çelikoğlu, Büşra Odabaş, Arif Daştan, Sefa Uçar*, Bilal Nişancı*

e-mail: sefa.ucar@atauni.edu.tr, bnisanci@atauni.edu.tr

Table of contents

General Experimental Details	SI1
General procedure A: Self-Etherification of Benzhydrols	SI1
General procedure B: Oxidation of Benzhydrols	SI1
General procedure C: Ritter-Type Amidation of Benzhydrols	SI1
Characterization for All Products	SI2-SI10
Scale-up of Self-Etherification	SI10
Scale-up of Oxidation	SI11
Scale-up of Ritter-Type Amidation	SI11
Raw ^{1}H NMR spectrum showing the formation of S-methyl methanesulfonothioate during the etherification of diphenylmethanol	SI11
References	SI12-SI13
NMR Spectra	SI14-SI53

General Experimental Details

All reactants, reagents, and solvents were obtained from commercial suppliers and used without further purification. Reactions were carried out in sealed 10 mL or 5 mL Schlenk tubes isolated from moisture and heated using a heating module (equipped with a magnetic stirrer). Work-up and purification procedures were carried out in air using reagent-grade solvents. Analytical thin-layer chromatography (TLC) was performed on silica gel $HSGF_{254}$ precoated plates (0.25 mm thickness), and chromatograms were visualized under UV light at 254 nm. Preparative thin-layer chromatography (PTLC) was conducted on self-prepared HuangHai GF_{254} silica plates (thickness 1.0 ± 0.03 mm). Column chromatography was performed over Merck Silica gel 60F (70-230 mesh ASTM). The ^{1}H- and ^{13}C-NMR spectra were recorded on a Varian-400 or a Bruker-400 spectrometer in $CDCl_3$ using tetramethylsilane as the internal reference. All spectra were recorded at 25 °C and coupling constants (*J* values) are given in Hz. Chemical shifts are given in parts per million (ppm). Abbreviations used to define the multiplicities are as follows: s = singlet; d = doublet; dd = doublet of doublets; m = multiplet. Mass spectra of unknown compounds were recorded on an Agilent Technologies 6530 Accurate-Mass Q-TOF-LC/MS. Melting points are uncorrected. Non-commercial alcohols were prepared by reduction of commercially available by Aldrich benzophenones with $LiAlH_4$ or $NaBH_4$, as described in the literature. For known compounds, only the ^{1}H NMR and ^{13}C NMR spectra are provided, whereas for unknown compounds, HRMS spectra and melting points (for solids) are additionally reported.

Safety Alert: Hydrofluoric acid (HF) is generated during these reactions. Handle with extreme care. Use appropriate personal protective equipment (PPE), including HF-resistant gloves, lab coat, and eye protection. Work in a well-ventilated fume hood and have calcium gluconate gel readily available in case of skin contact.

General procedure A: Self-Etherification of Benzhydrols

In a flame-dried Schlenk tube, to a solution of 1,2-dimethyldisulfane (21 mg, 220 μmol, 0.55 eq) in acetonitrile (0.044 M), Selectfluor (78 mg, 220 μmol, 0.55 eq) was added in a single portion, and the resulting mixture was stirred at room temperature for 10 minutes. A solution of benzhydrol (400 μmol, 1 eq) in acetonitrile (0.4 M) was then introduced, and the reaction was allowed to stir at ambient temperature for 17 hours. Upon completion, the reaction mixture was poured into a separatory funnel containing saturated aqueous $NaHCO_3$ (10 mL). The aqueous layer was extracted with ethyl acetate (2 × 20 mL), and the combined organic extracts were dried over anhydrous Na_2SO_4. The solvent was removed under reduced pressure to give the crude product, which was subsequently purified by preparative thin-layer chromatography (PTLC) using EtOAc/petroleum ether (1:4) as the eluent.

General procedure B: Oxidation of Benzhydrols

In a flame-dried Schlenk tube, to a solution of carbon disulfide (15 mg, 200 μmol, 0.5 eq) in acetonitrile (0.04 M), Selectfluor (142 mg, 400 μmol, 1 eq) was added in a single portion, followed by the addition of a solution of benzhydrol (400 μmol, 1 eq) in acetonitrile (0.4 M). The reaction mixture was stirred at 100 °C for 18 h. Upon completion, the mixture was poured into a separatory funnel containing saturated aqueous $NaHCO_3$ (10 mL). The aqueous phase was extracted with ethyl acetate (2 × 20 mL), and the combined organic layers were dried over anhydrous Na_2SO_4. Concentration under reduced pressure afforded the crude product, which was purified by preparative thin-layer chromatography (PTLC) using EtOAc/petroleum ether (3:7) as the eluent.

General procedure C: Ritter-Type Amidation of Benzhydrols

In a flame-dried Schlenk tube, to a solution of carbon disulfide (30 mg, 400 µmol, 1 eq) in a mixture of acetonitrile/nitromethane (3:1, 0.1 M), Selectfluor (35 mg, 100 µmol, 0.25 eq) was added in a single portion, followed by the addition of a solution of benzhydrol (400 µmol) in acetonitrile (0.4 M). The reaction mixture was stirred at 100 °C for 17 h. After completion, the mixture was transferred into a separatory funnel containing saturated aqueous $NaHCO_3$ (10 mL). The aqueous layer was extracted with ethyl acetate (2 × 20 mL), and the combined organic layers were dried over anhydrous Na_2SO_4. Removal of the solvent under reduced pressure afforded the crude product, which was purified by crystallization using EtOAc/petroleum ether (1:10).

Characterization for All Products

(Oxybis(methanetriyl))tetrabenzene (2a);

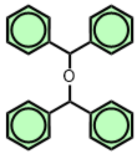

According to General Procedure A, the reaction was performed using 74 mg of diphenylmethanol, yielding 66 mg of **2a** (94% yield). Analytical data were in agreement with the literature[1,3,4]. **^{1}H NMR (400 MHz, Chloroform-*d*)** δ 7.49 – 7.20 (m, 20H), 5.44 (s, 2H). **^{13}C{^{1}H} NMR (100 MHz, Chloroform-*d*)** δ 142.5, 128.6, 127.7, 127.5, 80.2.

4,4'-(Oxybis(phenylmethylene))bis(bromobenzene) (2b);

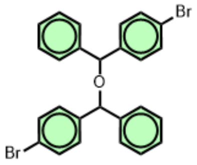

According to General Procedure A, the reaction was performed using 105 mg of (4-bromophenyl)(phenyl)methanol, yielding 91 mg of **2b** (90% yield). Analytical data were in agreement with the literature[1,3]. **^{1}H NMR (400 MHz, Chloroform-*d*)** δ 7.43 – 7.33 (m, 4H), 7.29 – 7.20 (m, 10H), 7.14 (dd, J = 8.4, 3.4 Hz, 4H), 5.24 (s, 2H). **^{13}C{^{1}H} NMR (100 MHz, Chloroform-*d*)** δ 141.6, 141.5, 141.4, 141.2, 131.85, 131.79, 129.1, 129.0, 128.86, 128.81, 128.1, 128.0, 127.4, 127.3, 121.75, 121.68, 79.76, 79.74.

4,4'-(Oxybis(phenylmethylene))bis(chlorobenzene) (2c);

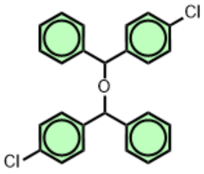

According to General Procedure A, the reaction was performed using 88 mg of (4-chlorophenyl)(phenyl)methanol, yielding 73 mg of **2c** (86% yield). Analytical data were in agreement with the literature[1]. **^{1}H NMR (400 MHz, Chloroform-*d*)** δ 8.29 – 6.16 (m, 18H), 5.38 (s, 2H). **^{13}C{^{1}H} NMR (100 MHz, Chloroform-*d*)** δ 141.7, 141.6, 140.9, 140.8, 133.6, 133.5, 128.9, 128.88, 128.87, 128.84, 128.80, 128.7, 128.1, 128.05, 127.5, 127.4, 79.76, 79.73.

4,4'-(Oxybis(phenylmethylene))bis(fluorobenzene) (2d);

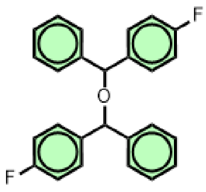

According to General Procedure A, the reaction was performed using 81 mg of (4-fluorophenyl)(phenyl)methanol, yielding 61 mg of **2d** (79% yield). Analytical data were in agreement with the literature[2,3]. **^{1}H NMR (400 MHz, Chloroform-*d*)** δ 7.58 – 7.26 (m, 14H), 7.08 – 7.03 (m, 4H), 5.42 (s, 2H). **^{13}C{^{1}H} NMR (100 MHz, Chloroform-*d*)** 162.46 (d, J = 245.6 Hz), 162.42 (d, J = 245.7 Hz), 142.09, 142.04, 138.19 (d, J = 3.1 Hz), 138.15 (d, J = 3.4 Hz), 129.17 (d, J = 7.0 Hz), 129.09 (d, J = 6.5 Hz), 128.84, 128.81, 128.00, 127.95, 127.46, 127.39, 115.61 (d, J = 21.4 Hz), 115.57 (d, J = 21.4 Hz), 79.72, 79.68.

4,4'-(Oxybis(phenylmethylene))bis(methoxybenzene) (2e);

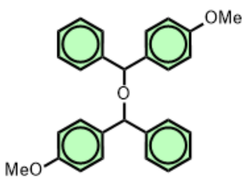

According to General Procedure A, the reaction was performed using 86 mg of (4-methoxyphenyl)(phenyl)methanol, yielding 26 mg of **2e** (32% yield). Analytical data were in agreement with the literature[1,3]. **^{1}H NMR (400 MHz, Chloroform-*d*)** δ 7.62 – 7.13 (m, 14H), 6.86 (d, J = 8.6 Hz, 4H), 5.34 (s, 2H), 3.79 (s, 6H). **^{13}C{^{1}H} NMR (100 MHz, Chloroform-*d*)** δ 159.2, 159.1, 142.9, 142.7, 134.7, 134.5, 128.9, 128.8, 128.6, 128.5, 127.51, 127.45, 127.37, 127.29, 114.00, 113.96, 79.61, 79.58, 55.5 (2C).

4,4'-(Oxybis(phenylmethylene))bis(methylbenzene) (2f);

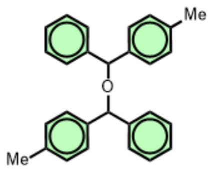

According to General Procedure A, the reaction was performed using 80 mg of phenyl(p-tolyl)methanol, yielding 68 mg of **2f** (89% yield). Analytical data were in agreement with the literature[1,2,3]. **^{1}H NMR (400 MHz, Chloroform-*d*)** δ 8.02 – 6.69 (m, 18H), 5.43 (s, 2H), 2.38 (s, 6H). **^{13}C{^{1}H} NMR (100 MHz, Chloroform-*d*)** δ 142.9, 142.8, 139.7, 139.5, 137.4, 137.3, 129.38, 129.35, 128.62, 128.60, 127.58 (2C), 127.54, 127.51, 127.47, 127.41, 80.0 (2C), 21.4 (2C).

4,4'-(Oxybis(phenylmethylene))bis(nitrobenzene) (2g);

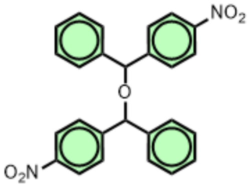

According to General Procedure A, the reaction was performed using 92 mg of (4-nitrophenyl)(phenyl)methanol at 80 °C, yielding 50 mg of **2g** (57% yield). Analytical data were in agreement with the literature[3]. **^{1}H NMR (400 MHz, Chloroform-*d*)** δ 8.26 – 8.12 (m, 4H), 7.63 – 7.48 (m, 4H), 7.44 – 7.27 (m, 10H), 5.46 (s, 2H). **^{13}C{^{1}H} NMR (100 MHz, Chloroform-*d*)** δ 149.5, 149.0, 147.7, 147.5, 140.3, 139.9, 129.3, 129.2, 129.0, 128.7, 127.9, 127.8, 127.6, 127.4, 124.2, 123.9, 80.0, 79.9. *N*-((4-nitrophenyl)(phenyl)methyl)acetamide (**4g**) was obtained as side product (35 mg, 32% yield).

4,4',4'',4'''-(Oxybis(methanetriyl))tetrakis(bromobenzene) (2h);

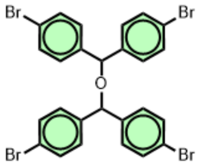

According to General Procedure A, the reaction was performed using 137 mg of bis(4-bromophenyl)methanol, yielding 116 mg of **2h** (87% yield, white solid, mp 156-158 °C). **^{1}H NMR (400 MHz, Chloroform-*d*)** δ 7.57 – 7.38 (m, 8H), 7.28 – 7.03 (m, 8H), 5.26 (s, 2H). **^{13}C{^{1}H} NMR (100 MHz, Chloroform-*d*)** δ 140.4, 132.1, 128.9, 122.2, 79.3.

4,4',4'',4'''-(Oxybis(methanetriyl))tetrakis(fluorobenzene) (2j);

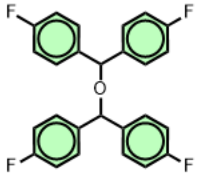

According to General Procedure A, the reaction was performed using 88 mg of bis(4-fluorophenyl)methanol, yielding 71 mg of **2j** (84% yield). Analytical data were in agreement with the literature[1,4]. **^{1}H NMR (400 MHz, Chloroform-*d*)** δ 7.39 – 7.21 (m, 8H), 7.16 – 6.88 (m, 8H), 5.33 (s, 2H). **^{13}C{^{1}H} NMR (100 MHz, Chloroform-*d*)** δ 162.5 (d, J = 246.3 Hz), 137.7 (d, J = 3.1 Hz), 129.0 (d, J = 8.0 Hz), 115.7 (d, J = 21.5 Hz), 79.1.

Dimethyl 4,4'-(oxybis(p-tolylmethylene))dibenzoate (2k);

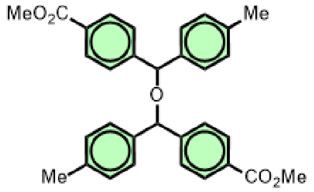

According to General Procedure A, the reaction was performed using 103 mg of methyl 4-(hydroxy(p-tolyl)methyl)benzoate at 80 °C, yielding 82 mg of **2k** (83% yield, viscous liquid). **^{1}H NMR (400 MHz, Chloroform-*d*)** δ 8.00 (d, J = 4.1 Hz, 2H), 7.98 (d, J = 4.2 Hz, 2H), 7.44 (d, J = 8.0 Hz, 4H), 7.22 (d, J = 3.6 Hz, 2H), 7.20 (d, J = 3.6 Hz, 2H), 7.15 (d, J = 3.8 Hz,

2H), 7.13 (d, J = 3.7 Hz, 2H), 5.39 (s, 2H), 3.90 (s, 6H), 2.34 (s, 3H), 2.33 (s, 3H). **^{13}C{^{1}H} NMR (100 MHz, Chloroform-*d*)** δ 167.18, 167.15, 147.8, 147.5, 138.5, 138.2, 138.0, 137.9, 130.0, 129.9, 129.6, 129.5, 129.3 (2C), 127.6, 127.4, 127.2, 127.0, 79.9, 79.8, 52.35, 52.34, 21.42, 21.40. **HRMS (ESI) m/z: [M + Na]$^+$** calcd for $C_{32}H_{30}O_5$, 517.1985; found, 517.2007.

4,4'-(Oxybis((4-chlorophenyl)methylene))bis(methylbenzene) (2l);

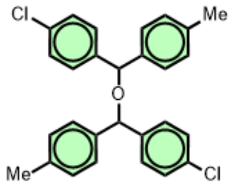

According to General Procedure A, the reaction was performed using 93 mg of (4-chlorophenyl)(p-tolyl)methanol, yielding 72 mg of **2l** (81% yield). Analytical data were in agreement with the literature[4]. **^{1}H NMR (400 MHz, Chloroform-*d*)** δ 7.33 – 7.25 (m, 8H), 7.25 – 7.08 (m, 8H), 5.31 (s, 2H), 2.34 (s, 3H), 2.33 (s, 3H). **^{13}C{^{1}H} NMR (100 MHz, Chloroform-*d*)** δ 141.2, 141.0, 138.8, 138.6, 137.8, 137.7, 133.4, 133.3, 129.5, 129.4, 128.8, 128.7, 128.69, 128.6, 127.5, 127.3, 79.4 (2C), 21.4, 21.39.

5,5'-Oxybis(10,11-dihydro-5H-dibenzo[a,d][7]annulene) (2r);

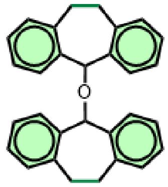

According to General Procedure A, the reaction was performed using 84 mg of 10,11-dihydro-5*H*-dibenzo[*a,d*][7]annulen-5-ol, yielding 64 mg of **2r** (80% yield). Reaction time was 6 hour. Analytical data were in agreement with the literature[5]. **^{1}H NMR (400 MHz, Chloroform-*d*)** δ 7.21 – 7.11 (m, 16H), 5.37 (bs, 2H), 4.17 – 3.36 (bs, 4H), 2.92 (bs, 4H). **^{13}C{^{1}H} NMR (100 MHz, Chloroform-*d*)** δ 140.5, 138.6, 130.6, 128.3, 128.30, 126.0, 83.4, 32.2.

5,5'-Oxybis(5H-dibenzo[a,d][7]annulene) (2s);

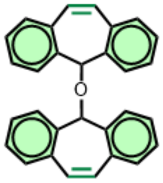

According to General Procedure A, the reaction was performed using 84 mg of 5*H*-dibenzo[*a,d*][7]annulen-5-ol, yielding 66 mg of **2s** (82% yield). Reaction time was 1 hour. Analytical data were in agreement with the literature[6]. Exo–exo conformer (**4**:1); **^{1}H NMR (400 MHz, Chloroform-*d*)** δ 7.96 (d, J = 7.8 Hz, 4H), 7.44 – 7.40 (m, 4H), 7.32 – 7.29 (m, 4H), 7.27 – 7.23 (m, 4H), 7.02 (s, 4H), 5.01 (s, 2H). **^{13}C{^{1}H} NMR (100 MHz, Chloroform-*d*)** δ 139.5, 132.8, 131.4, 129.0, 128.0, 126.4, 122.5, 77.0. Exo–endo conformer ((**1**:4), not all signals could be assigned unambiguously due to low intensity and overlap with signals of main conformer, only selectable signals); **^{1}H NMR (400 MHz, Chloroform-*d*)** δ 5.60 (s, 1H), 4.58 (s, 1H). **^{13}C{^{1}H} NMR (100 MHz, Chloroform-*d*)** δ 131.2, 130.8, 130.3, 129.7, 128.5, 128.1, 128.0, 127.7, 126.1, 123.3.

Benzophenone (3a);

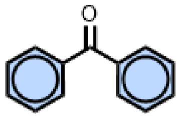

According to General Procedure B, the reaction was performed using 74 mg of diphenylmethanol, yielding 69 mg of **3a** (94% yield). Analytical data were in agreement with the literature[7]. **^{1}H NMR (400 MHz, Chloroform-*d*)** δ 7.88 – 7.69 (m, 4H), 7.60 – 7.54 (m, 2H), 7.51 – 7.42 (m, 4H). **^{13}C{^{1}H} NMR (100 MHz, Chloroform-*d*)** δ 197.0, 137.8, 132.7, 130.3, 128.5.

(4-Bromophenyl)(phenyl)methanone (3b);

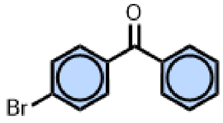

According to General Procedure B, the reaction was performed using 105 mg of (4-bromophenyl)(phenyl)methanol (400 µmol), carbon disulfide (46 mg, 600 µmol) and Selectfluor (425 mg, 1.2 mmol) yielding 92 mg of **3b** (88% yield). Analytical data were in agreement with the literature[8]. **^{1}H NMR (400 MHz, Chloroform-*d*)** δ 7.82 – 7.74 (m, 2H), 7.71 – 7.65 (m, 2H), 7.64 – 7.55 (m, 3H), 7.50 – 7.45 (m, 2H). **^{13}C{^{1}H} NMR (100 MHz, Chloroform-*d*)** δ 195.8, 137.4, 136.5, 132.9, 131.85, 131.80, 130.2, 128.6, 127.7.

(4-Chlorophenyl)(phenyl)methanone (3c);

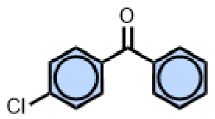

According to General Procedure B, the reaction was performed using 87 mg of (4-chlorophenyl)(phenyl)methanol (400 µmol), carbon disulfide (46 mg, 600 µmol) and Selectfluor (425 mg, 1.2 mmol) yielding 78 mg of **3c** (91% yield). Analytical data were in agreement with the literature[7]. **^{1}H NMR (400 MHz, Chloroform-*d*)** δ 7.82 – 7.69 (m, 4H), 7.67 – 7.57 (m, 1H), 7.56 – 7.40 (m, 4H). **^{13}C{^{1}H} NMR (100 MHz, Chloroform-*d*)** δ 195.8, 139.1, 137.4, 136.1, 132.9, 131.7, 130.2, 128.9, 128.6.

(4-Fluorophenyl)(phenyl)methanone (3d);

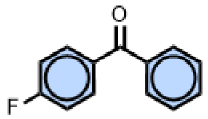

According to General Procedure B, the reaction was performed using 81 mg of (4-fluorophenyl)(phenyl)methanol, yielding 56 mg of **3d** (70% yield). Analytical data were in agreement with the literature[7]. **^{1}H NMR (400 MHz, Chloroform-*d*)** δ 7.80 – 7.73 (m, 2H), 7.71 – 7.63 (m, 2H), 7.55 – 7.45 (m, 1H), 7.39 (t, J = 7.6 Hz, 2H), 7.06 (t, J = 8.6 Hz, 2H). **^{13}C{^{1}H} NMR (100 MHz, Chloroform-*d*)** δ 195.5, 165.6 (d, J = 253.7 Hz), 137.7, 134.0 (d, J = 3.5 Hz), 132.9 (d, J = 9.2 Hz), 132.7, 130.1, 128.6, 115.7 (d, J = 21.9 Hz). *N*-((4-fluorophenyl)(phenyl)methyl)acetamide (**4d**) was obtained as side product (25 mg, 26% yield).

(4-Methoxyphenyl)(phenyl)methanone (3e);

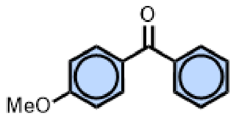

According to General Procedure B, the reaction was performed using 85 mg of (4-methoxyphenyl)(phenyl)methanol, yielding 37 mg of **3e** (44% yield). Analytical data were in agreement with the literature[7]. **^{1}H NMR (400 MHz, Chloroform-*d*)** δ 7.88 – 7.81 (m, 2H), 7.79 – 7.73 (m, 2H), 7.63 – 7.53 (m, 1H), 7.51 – 7.44 (m, 2H), 7.08 – 6.90 (m, 2H), 3.89 (s, 3H). **^{13}C{^{1}H} NMR (100 MHz, Chloroform-*d*)** δ 195.8, 163.4, 138.5, 132.8, 132.1, 130.4, 130.0, 128.4, 113.8, 55.7. *N*-((4-methoxyphenyl)(phenyl)methyl)acetamide (**4e**) was obtained as side product (40 mg, 40% yield).

(4-nitrophenyl)(phenyl)methanone (3g);

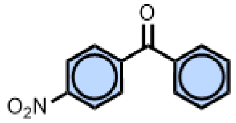

According to General Procedure B, the reaction was performed using 92 mg of (4-nitrophenyl)(phenyl)methanol, yielding 72 mg of **3g** (79% yield). Analytical data were in agreement with the literature[8]. **^{1}H NMR (400 MHz, Chloroform-*d*)** δ 8.38 – 8.27 (m, 2H), 8.00 – 7.88 (m, 2H), 7.86 – 7.72 (m, 2H), 7.71 – 7.62 (m, 1H), 7.56 – 7.51 (m, 2H). **^{13}C{^{1}H} NMR (100 MHz, Chloroform-*d*)** δ 195.1, 150.1, 143.1, 136.5, 133.7, 130.9, 130.3, 128.9, 123.8. *N*-((4-nitrophenyl)(phenyl)methyl)acetamide (**4g**) was obtained as side product (21 mg, 19% yield).

Bis(4-bromophenyl)methanone (3h);

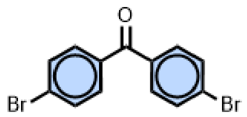

According to General Procedure B, the reaction was performed using 137 mg of bis(4-bromophenyl)methanol, yielding 117 mg of **3h** (86% yield). Analytical data were in agreement with the literature[9]. **^{1}H NMR (400 MHz, Chloroform-*d*)** δ 7.64 (s, 8H). **^{13}C{^{1}H} NMR (100 MHz, Chloroform-*d*)** δ 194.7, 136.1, 132.0, 131.6, 128.1.

Bis(4-fluorophenyl)methanone (3j);

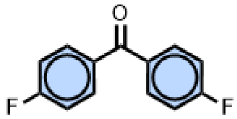

According to General Procedure B, the reaction was performed using 88 mg of bis(4-fluorophenyl)methanol (400 µmol), carbon disulfide (46 mg, 600 µmol) and Selectfluor (425 mg, 1.2 mmol) yielding 78 mg of **3j** (89% yield). Analytical data were in agreement with the literature[10]. **^{1}H NMR (400 MHz, Chloroform-*d*)** δ 8.57 – 7.55 (m, 4H), 7.47 – 6.63 (m, 4H). **^{13}C{^{1}H} NMR (100 MHz, Chloroform-*d*)** δ 194.0, 165.6 (d, J = 254.5 Hz), 133.9 (d,

J = 2.6 Hz), 132.7 (d, J = 9.2 Hz), 115.8 (d, J = 21.8 Hz). *N*-(bis(4-fluorophenyl)methyl)acetamide (**4j**) was obtained as side product (trace).

Methyl 4-(4-methylbenzoyl)benzoate (3k);

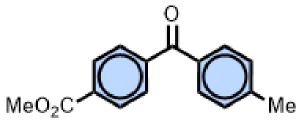

According to General Procedure B, the reaction was performed using 103 mg of methyl 4-(hydroxy(p-tolyl)methyl)benzoate (400 µmol), carbon disulfide (46 mg, 600 µmol) and Selectfluor (425 mg, 1.2 mmol) yielding 68 mg of **3k** (67% yield). Analytical data were in agreement with the literature[11]. **^{1}H NMR (400 MHz, Chloroform-*d*)** δ 8.14 (d, J = 8.2 Hz, 2H), 7.81 (d, J = 8.2 Hz, 2H), 7.71 (d, J = 8.0 Hz, 2H), 7.30 (d, J = 7.9 Hz, 2H), 3.96 (s, 3H), 2.45 (s, 3H). **^{13}C{^{1}H} NMR (100 MHz, Chloroform-*d*)** δ 196.0, 166.6, 144.2, 141.9, 134.5, 133.2, 130.6, 129.9, 129.7, 129.4, 52.7, 21.9.

(4-Chlorophenyl)(p-tolyl)methanone (3l);

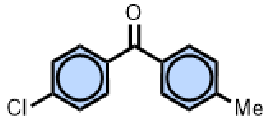

According to General Procedure B, the reaction was performed using 93 mg of (4-chlorophenyl)(p-tolyl)methanol at 110 °C, yielding 28 mg of **3l** (30% yield). Analytical data were in agreement with the literature[12]. **^{1}H NMR (400 MHz, Chloroform-*d*)** δ 7.73 (d, J = 8.5 Hz, 2H), 7.69 (d, J = 8.1 Hz, 2H), 7.45 (d, J = 8.5 Hz, 2H), 7.29 (d, J = 8.1 Hz, 2H), 2.44 (s, 3H). **^{13}C{^{1}H} NMR (100 MHz, Chloroform-*d*)** δ 195.5, 143.8, 138.8, 136.4, 134.7, 131.6, 130.4, 129.3, 128.8, 21.9. *N*-((4-chlorophenyl)(p-tolyl)methyl)acetamide (**4l**) was obtained as main product (68 mg, 62% yield).

4-(4-Chlorobenzoyl)benzonitrile (3m);

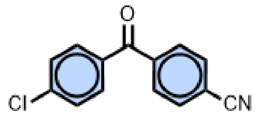

According to General Procedure B, the reaction was performed using 97 mg of 4-((4-chlorophenyl)(hydroxy)methyl)benzonitrile, yielding 91 mg of **3l** (95% yield). Analytical data were in agreement with the literature[13]. **^{1}H NMR (400 MHz, Chloroform-*d*)** δ 7.88 – 7.83 (m, 2H), 7.82 – 7.77 (m, 2H), 7.76 – 7.69 (m, 2H), 7.54 – 7.45 (m, 2H). **^{13}C{^{1}H} NMR (100 MHz, Chloroform-*d*)** δ 194.0, 141.1, 140.2, 134.8, 132.5, 131.6, 130.3, 129.3, 118.1, 116.1.

3-(4-Bromobenzoyl)benzonitrile (3n);

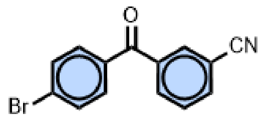

According to General Procedure B, the reaction was performed using 115 mg of 3-((4-bromophenyl)(hydroxy)methyl)benzonitrile, yielding 104 mg of **3n** (91% yield). Analytical data were in agreement with the literature[14]. **^{1}H NMR (400 MHz, Chloroform-*d*)** δ 8.04 (s, 1H), 8.01 (d, J = 8.0 Hz, 1H), 7.88 (d, J = 7.7 Hz, 1H), 7.75 – 7.57 (m, 5H). **^{13}C{^{1}H} NMR (100 MHz, Chloroform-*d*)** δ 193.6, 138.4, 135.8, 135.2, 133.9, 133.5, 132.3, 131.7, 129.8, 128.8, 118.0, 113.3.

10,11-Dihydro-5H-dibenzo[a,d][7]annulen-5-one (3s);

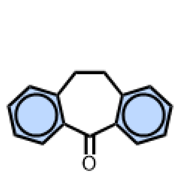

According to General Procedure B, the reaction was performed using 85 mg of 10,11-dihydro-5*H*-dibenzo[*a,d*][7]annulen-5-ol, yielding 70 mg of **3s** (83% yield). Analytical data were in agreement with the literature[15]. **^{1}H NMR (400 MHz, Chloroform-*d*)** δ 8.02 (dd, J = 7.8, 1.5 Hz, 2H), 7.43 (td, J = 7.4, 1.5 Hz, 2H), 7.33 (td, J = 7.6, 1.4 Hz, 2H), 7.22 (dd, J = 7.6, 1.3 Hz, 2H), 3.21 (s, 4H). **^{13}C{^{1}H} NMR (100 MHz, Chloroform-*d*)** δ 195.9, 142.2, 138.9, 132.6, 130.8, 129.5, 126.8, 35.2. *N*-(10,11-dihydro-5*H*-dibenzo[*a,d*][7]annulen-5-yl)acetamide (**4s**) was obtained as side product (trace).

Oxidation of 5*H*-dibenzo[*a,d*][7]annulen-5-ol

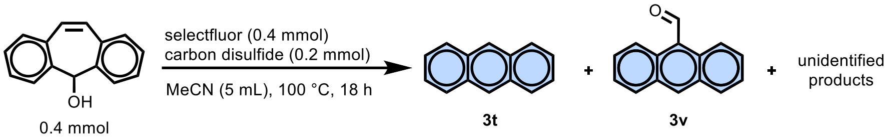

According to General Procedure B, the reaction was performed using 84 mg of 5*H*-dibenzo[*a,d*][7]annulen-5-ol. After purification by preparative thin-layer chromatography (PTLC) using EtOAc/petroleum ether (1:4) as the eluent, anthracene (**3t**) was obtained (not pure). Anthracene-9-carbaldehyde (**3v**) could not be isolated; however, its formation was indicated by characteristic signals observed in the ^{1}H NMR spectrum of the crude reaction mixture, which were consistent with literature data[20]. The presence of additional unidentified products is also evident from the ^{1}H NMR spectrum of the crude reaction mixture.

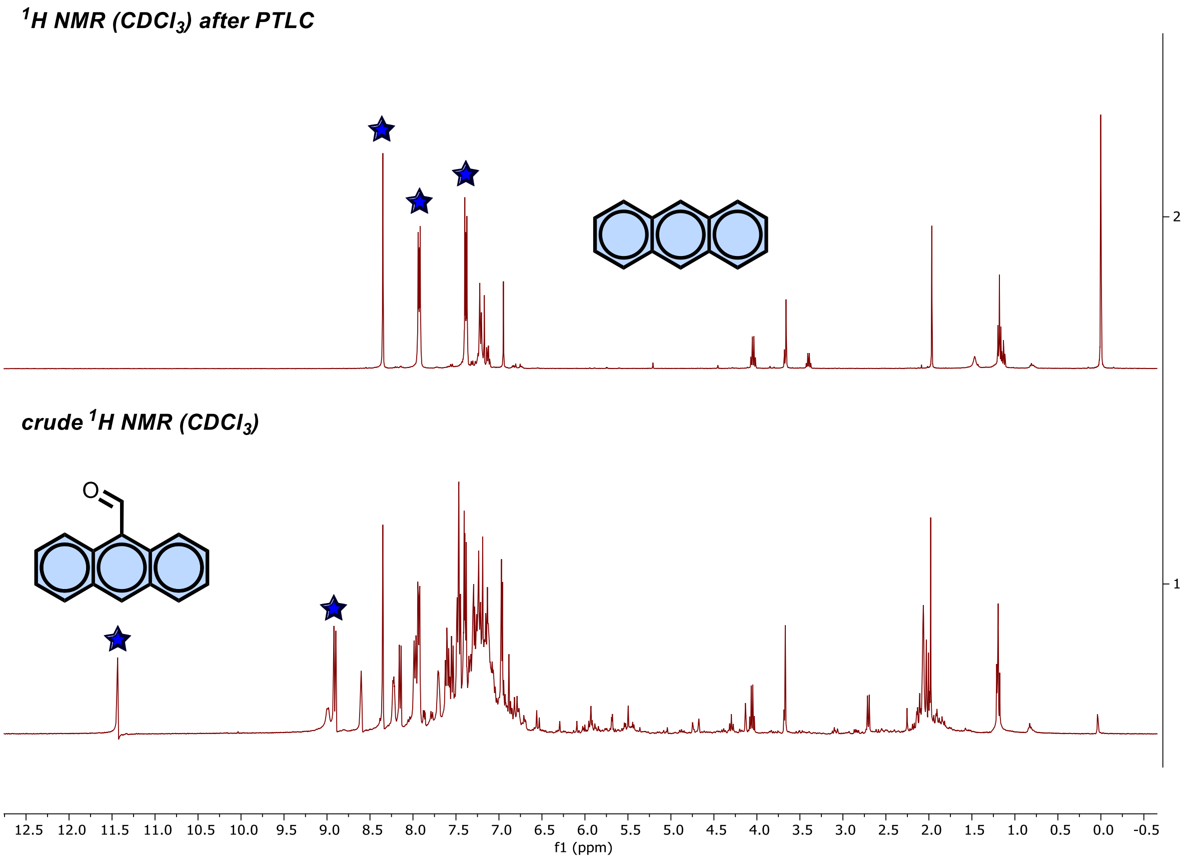

***N*-benzhydrylacetamide (4a);**

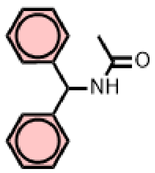

According to General Procedure C, the reaction was performed using 74 mg of diphenylmethanol, yielding 84 mg of **4a** (93% yield). Analytical data were in agreement with the literature[16]. **^{1}H NMR (400 MHz, Chloroform-*d*)** δ 7.39 – 7.15 (m, 10H), 6.33 (bs, 1H), 6.24 (d, J = 8.1 Hz, 1H), 2.01 (s, 3H). **^{13}C{^{1}H} NMR (100 MHz, Chloroform-*d*)** δ 169.4, 141.8, 128.9, 127.7 (2C), 57.2, 23.5.

N-((4-bromophenyl)(phenyl)methyl)acetamide (4b);

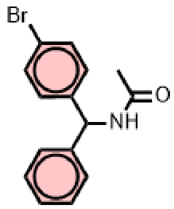

According to General Procedure C, the reaction was performed using 105 mg of (4-bromophenyl)(phenyl)methanol, yielding 83 mg of **4b** (68% yield). Analytical data were in agreement with the literature[16]. **^{1}H NMR (400 MHz, Chloroform-*d*)** δ 7.44 (d, J = 8.4 Hz, 2H), 7.37 – 7.24 (m, 3H), 7.22 – 7.15 (m, 2H), 7.09 (d, J = 8.4 Hz, 2H), 6.30 (bs, 1H), 6.16 (d, J = 7.8 Hz, 1H), 2.04 (s, 3H). **^{13}C{^{1}H} NMR (100 MHz, Chloroform-*d*)** δ 169.6, 141.1, 140.7, 132.0, 129.3, 129.1, 128.1, 127.7, 121.6, 56.9, 23.4. (4-bromophenyl)(phenyl)methanone (**3b**) was obtained as side product (24 mg, 23% yield).

N-((4-chlorophenyl)(phenyl)methyl)acetamide (4c);

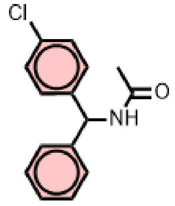

According to General Procedure C, the reaction was performed using 88 mg of (4-chlorophenyl)(phenyl)methanol, yielding 88 mg of **4c** (84% yield). Analytical data were in agreement with the literature[16]. **^{1}H NMR (400 MHz, Chloroform-*d*)** δ 7.37 – 7.24 (m, 5H), 7.19 – 7.17 (m, 2H), 7.15 (d, J = 8.5 Hz, 2H), 6.40 (d, J = 7.8 Hz, 1H), 6.18 (d, J = 7.8 Hz, 1H), 2.03 (s, 3H). **^{13}C{^{1}H} NMR (100 MHz, Chloroform-*d*)** δ 169.7, 141.1, 140.2, 133.5, 129.1, 129.0, 128.96, 128.0, 127.7, 56.8, 23.4.

N-((4-fluorophenyl)(phenyl)methyl)acetamide (4d);

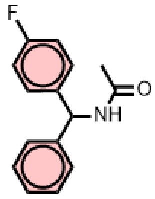

According to General Procedure C, the reaction was performed using 81 mg of (4-fluorophenyl)(phenyl)methanol, yielding 87 mg of **4d** (89% yield). Analytical data were in agreement with the literature[17]. **^{1}H NMR (400 MHz, Chloroform-*d*)** δ 7.38 – 7.24 (m, 3H), 7.22 – 7.14 (m, 4H), 7.00 (t, J = 8.7 Hz, 2H), 6.25 – 6.20 (m, J = 7.6 Hz, 2H), 2.03 (s, 3H). **^{13}C{^{1}H} NMR (100 MHz, Chloroform-*d*)** δ 169.4, 162.3 (d, J = 246.0 Hz), 141.5, 137.5 (d, J = 3.3 Hz), 129.3 (d, J = 8.0 Hz), 129.0, 127.9, 127.6, 115.7 (d, J = 21.4 Hz), 56.6, 23.5. (4-fluorophenyl)(phenyl)methanone (**3d**) was obtained as side product (trace).

N-((4-methoxyphenyl)(phenyl)methyl)acetamide (4e);

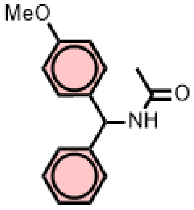

According to General Procedure C, the reaction was performed using 86 mg of (4-methoxyphenyl)(phenyl)methanol, yielding 35 mg of **4e** (34% yield). Reaction time was 14 hour. Analytical data were in agreement with the literature[17]. **^{1}H NMR (400 MHz, Chloroform-*d*)** δ 7.31 – 7.07 (m, 5H), 7.02 (d, J = 8.6 Hz, 2H), 6.73 (d, J = 8.7 Hz, 2H), 6.46 (d, J = 8.1 Hz, 1H), 6.07 (d, J = 8.3 Hz, 1H), 3.67 (s, 3H), 1.88 (s, 3H). The product could not be isolated in an absolutely pure form; however, the selected characteristic signals were found to be consistent with the literature data. Therefore, the **^{13}C{^{1}H} NMR** spectrum was not reported.

N-(phenyl(p-tolyl)methyl)acetamide (4f);

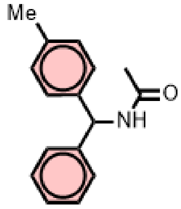

According to General Procedure C, the reaction was performed using 80 mg of phenyl(p-tolyl)methanol, yielding 89 mg of **4f** (92% yield). Analytical data were in agreement with the literature[17]. **^{1}H NMR (400 MHz, Chloroform-*d*)** δ 7.44 – 7.18 (m, 5H), 7.16 – 6.99 (m, 4H), 6.25 (d, J = 8.0 Hz, 1H), 6.19 (d, J = 8.0 Hz, 1H), 2.33 (s, 3H), 2.04 (s, 3H). **^{13}C{^{1}H} NMR (100 MHz, Chloroform-*d*)** δ 169.5, 141.8, 138.7, 137.5, 129.6, 128.9, 127.63, 127.60, 127.55, 57.0, 23.5, 21.3.

N-((4-nitrophenyl)(phenyl)methyl)acetamide (4g);

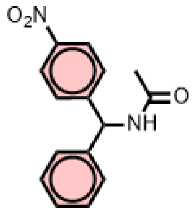

According to General Procedure C, the reaction was performed using 92 mg of (4-nitrophenyl)(phenyl)methanol, yielding 90 mg of **4g** (83% yield). Analytical data were in agreement with the literature[18]. **^{1}H NMR (400 MHz, Chloroform-*d*)** δ 8.14 (d, J = 8.5 Hz, 2H), 7.39 (d, J = 8.7 Hz, 2H), 7.37 – 7.28 (m, 3H), 7.17 (d, J = 7.7 Hz, 2H), 6.53 (bs, 1H), 6.25 (d, J = 7.5 Hz, 1H), 2.04 (s, 3H). **^{13}C{^{1}H} NMR (100 MHz, Chloroform-*d*)** δ 169.9, 149.1, 147.3, 140.3, 129.4, 128.5, 128.2, 127.9, 124.0, 57.2, 23.4. (4-nitrophenyl)(phenyl)methanone (**3g**) was obtained as side product (trace).

N-(bis(4-bromophenyl)methyl)acetamide (4h);

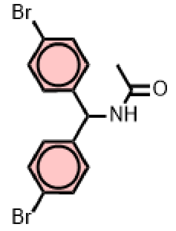

According to General Procedure C, the reaction was performed using 137 mg of bis(4-bromophenyl)methanol, yielding 89 mg of **4h** (58% yield, white solid, mp 192-194 °C). **^{1}H NMR (400 MHz, Chloroform-*d*)** δ 7.44 (d, J = 8.4 Hz, 4H), 7.06 (d, J = 8.4 Hz, 4H), 6.18 (d, J = 8.0 Hz, 1H), 6.12 (d, J = 8.0 Hz, 1H), 2.03 (s, 3H). **^{13}C{^{1}H} NMR (100 MHz, Chloroform-*d*)** δ 169.5, 140.2, 132.1, 129.3, 122.0, 56.3, 23.5. **HRMS (ESI) m/z: [M + H]$^+$** calcd for $C_{15}H_{13}Br_2NO$, 381.9437; found, 381.9449. Bis(4-bromophenyl)methanone (**3h**) was obtained as side product (54 mg, 40% yield).

N-(bis(4-fluorophenyl)methyl)acetamide (4j);

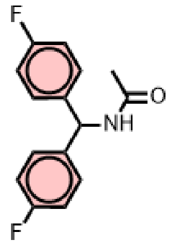

According to General Procedure C, the reaction was performed using 88 mg of bis(4-fluorophenyl)methanol, yielding 80 mg of **4j** (77% yield). Analytical data were in agreement with the literature[18]. **^{1}H NMR (400 MHz, Chloroform-*d*)** δ 7.18 – 7.14 (m, 4H), 7.03 – 6.99 (m, 4H), 6.22 (bs, 1H), 6.18 (d, J = 7.5 Hz, 1H), 2.05 (s, 3H). **^{13}C{^{1}H} NMR (100 MHz, Chloroform-*d*)** δ 169.5, 162.3 (d, J = 246.5 Hz), 137.2 (d, J = 3.3 Hz), 129.3 (d, J = 8.4 Hz), 115.9 (d, J = 21.6 Hz), 56.1, 23.5. Bis(4-fluorophenyl)methanone (**3j**) was obtained as side product (13 mg, 15% yield).

Methyl 4-(acetamido(p-tolyl)methyl)benzoate (4k);

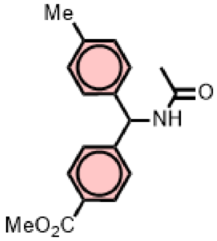

According to General Procedure C, the reaction was performed using 103 mg of methyl 4-(hydroxy(p-tolyl)methyl)benzoate, yielding 104 mg of **4k** (87% yield, white solid, mp 158-160 °C). **^{1}H NMR (400 MHz, Chloroform-*d*)** δ 7.99 (d, J = 8.0 Hz, 2H), 7.31 (d, J = 8.0 Hz, 2H), 7.14 (d, J = 7.9 Hz, 2H), 7.07 (d, J = 7.9 Hz, 2H), 6.24 (d, J = 7.8 Hz, 1H), 6.00 (d, J = 7.8 Hz, 1H), 3.90 (s, 3H), 2.33 (s, 3H), 2.08 (s, 3H). **^{13}C{^{1}H} NMR (100 MHz, Chloroform-*d*)** δ 169.3, 167.0, 146.9, 138.2, 138.0, 130.1, 129.8, 129.4, 127.8, 127.4, 57.0, 52.4, 23.6, 21.3. **HRMS (ESI) m/z: [M + H]$^+$** calcd for $C_{18}H_{19}NO_3$, 298.1438; found, 298.1448. Methyl 4-(4-methylbenzoyl)benzoate (**3k**) was obtained as side product (8 mg, 8% yield).

N-((4-chlorophenyl)(p-tolyl)methyl)acetamide (4l);

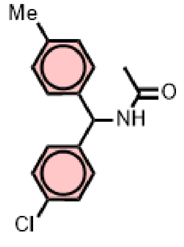

According to General Procedure C, the reaction was performed using 93 mg of (4-chlorophenyl)(p-tolyl)methanol, yielding 103 mg of **4l** (94% yield). Analytical data were in agreement with the literature[19]. **^{1}H NMR (400 MHz, Chloroform-*d*)** δ 7.33 – 7.22 (m, 2H), 7.17 – 7.10 (m, 4H), 7.06 (d, J = 8.1 Hz, 2H), 6.36 (bs, 1H), 6.13 (d, J = 7.9 Hz, 1H), 2.33 (s, 3H), 2.00 (s, 3H). **^{13}C{^{1}H} NMR (100 MHz, Chloroform-*d*)** δ 169.5, 140.5, 138.3, 137.8, 133.3, 129.7, 128.9, 128.9, 127.6, 56.5, 23.5, 21.3.

***N*-((4-chlorophenyl)(4-cyanophenyl)methyl)acetamide (4m);**

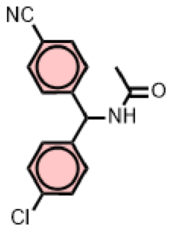

According to General Procedure C, the reaction was performed using 97 mg of 4-((4-chlorophenyl)(hydroxy)methyl)benzonitrile, yielding 83 mg of **4m** (73% yield, white solid, mp 137-139 °C). **^{1}H NMR (400 MHz, Chloroform-*d*)** δ 7.62 (d, J = 8.3 Hz, 2H), 7.32 (d, J = 8.3 Hz, 2H), 7.33 (d, J = 8.3 Hz, 2H), 7.11 (d, J = 8.3 Hz, 2H), 6.23 (d, J = 7.6 Hz, 1H), 6.11 (d, J = 7.6 Hz, 1H), 2.08 (s, 3H). **^{13}C{^{1}H} NMR (100 MHz, Chloroform-*d*)** δ 169.6, 146.4, 138.8, 134.4, 132.8, 129.5, 129.2, 128.2, 118.7, 111.8, 56.6, 23.5. **HRMS (ESI) m/z: [M + H]$^+$** calcd for $C_{16}H_{13}ClN_2O$, 285.0789; found, 285.0803. 4-(4-chlorobenzoyl)benzonitrile (**3m**) was obtained as side product (19 mg, 20% yield).

***N*-((4-bromophenyl)(3-cyanophenyl)methyl)acetamide (4n);**

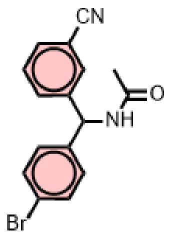

According to General Procedure C, the reaction was performed using 115 mg of 3-((4-bromophenyl)(hydroxy)methyl)benzonitrile, yielding 117 mg of **4n** (89% yield, white solid, mp 143-145 °C). **^{1}H NMR (400 MHz, Chloroform-*d*)** δ 7.58 – 7.40 (m, 6H), 7.05 (d, J = 8.4 Hz, 2H), 6.43 (d, J = 7.8 Hz, 1H), 6.18 (d, J = 7.7 Hz, 1H), 2.05 (s, 3H). **^{13}C{^{1}H} NMR (100 MHz, Chloroform-*d*)** δ 169.7, 142.8, 139.5, 132.4, 132.2, 131.6, 130.8, 129.8, 129.5, 122.5, 118.8, 113.0, 56.3, 23.4. **HRMS (ESI) m/z: [M + H]$^+$** calcd for $C_{16}H_{13}BrN_2O$, 329.0284; found, 329.0290.

***N*-((2-chlorophenyl)(phenyl)methyl)acetamide (4o);**

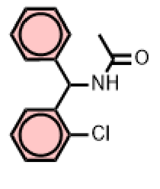

According to General Procedure C, the reaction was performed using 88 mg of (2-chlorophenyl)(phenyl)methanol, yielding 98 mg of **4o** (94% yield, white solid, mp 176-178 °C). **^{1}H NMR (400 MHz, Chloroform-*d*)** δ 7.61 – 6.96 (m, 9H), 6.51 (d, J = 7.8 Hz, 1H), 6.30 (d, J = 7.8 Hz, 1H), 2.04 (s, 3H). **^{13}C{^{1}H} NMR (100 MHz, Chloroform-*d*)** δ 169.4, 140.3, 138.8, 133.9, 130.5, 129.1, 129.1, 128.9, 127.8, 127.6, 127.2, 55.3, 23.4. **HRMS (ESI) m/z: [M + H]$^+$** calcd for $C_{15}H_{14}ClNO$, 260.0837; found, 260.0850.

***N*-(10,11-dihydro-5H-dibenzo[a,d][7]annulen-5-yl)acetamide (4s);**

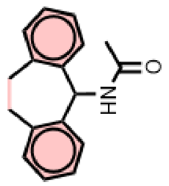

According to General Procedure C, the reaction was performed using 84 mg of 10,11-dihydro-5*H*-dibenzo[*a,d*][7]annulen-5-ol, yielding 95 mg of **4s** (95% yield, white solid, mp 273-275 °C). **^{1}H NMR (400 MHz, Chloroform-*d*)** δ 7.42 (dd, J = 7.0, 2.0 Hz, 2H), 7.24 – 7.12 (m, 6H), 6.30 (bs, 1H), 6.24 (d, J = 7.7 Hz, 1H), 3.48 – 3.24 (m, 2H), 3.20 – 2.99 (m, 2H), 1.97 (s, 3H). **^{13}C{^{1}H} NMR (100 MHz, Chloroform-*d*)** δ 168.7, 139.3, 138.4, 130.6, 130.3, 128.3, 126.8, 58.9, 33.2, 23.7. **HRMS (ESI) m/z: [M + Na]$^+$** calcd for $C_{17}H_{17}NO$, 274.1202; found, 274.1214.

Scale-up of Self-Etherification

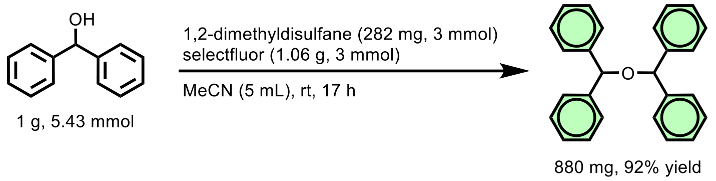

In a round-bottom flask, 1,2-dimethyldisulfane (282 mg, 3 mmol) was dissolved in acetonitrile (5 mL). Selectfluor (1.06 g, 3 mmol) was added in one portion, and the resulting mixture was stirred at room temperature for 10 minutes. A solution of diphenylmethanol (1 g, 5.43 mmol) in acetonitrile (1 mL) was then introduced, and the reaction was allowed to stir at ambient temperature for 17 hours. Upon completion, the reaction mixture was poured into a separatory funnel containing saturated aqueous

$NaHCO_3$ (15 mL). The aqueous layer was extracted with ethyl acetate (2 × 50 mL), and the combined organic extracts were dried over anhydrous Na_2SO_4. The solvent was removed under reduced pressure to give the crude product, which was subsequently purified by column chromatography using EtOAc/petroleum ether (1:4) as the eluent. Pure (oxybis(methanetriyl))tetrabenzene (**2a**) was obtained as a solid (910 mg, 92% yield).

Scale-up of Oxidation

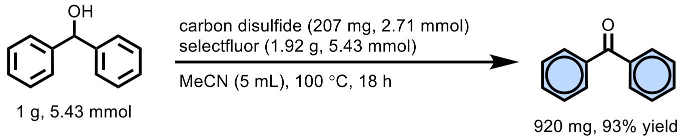

In a pressure tube, carbon disulfide (207 mg, 2.71 mmol) was dissolved in acetonitrile (5 mL). Selectfluor (1.92 g, 5.43 mmol) was added in one portion, followed by the addition of a solution of diphenylmethanol (1 g, 5.43 mmol) in acetonitrile (1 mL). The reaction mixture was stirred at 100 °C for 18 h. Upon completion, the mixture was poured into a separatory funnel containing saturated aqueous $NaHCO_3$ (15 mL). The aqueous phase was extracted with ethyl acetate (2 × 50 mL), and the combined organic layers were dried over anhydrous Na_2SO_4. Concentration under reduced pressure afforded the crude product, which was purified by column chromatography using EtOAc/petroleum ether (3:7) as the eluent. Pure benzophenone (**3a**) was obtained as a solid (920 mg, 93% yield).

Scale-up of Ritter-Type Amidation

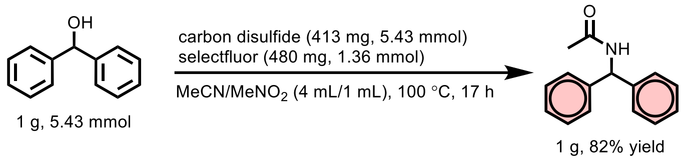

In a flame-dried Schlenk tube, carbon disulfide (413 mg, 5.43 mmol) was dissolved in a mixture of acetonitrile/nitromethane (3 mL/1 mL). Selectfluor (480 mg, 1.36 mmol) was added in one portion, followed by the addition of a solution of diphenylmethanol (1g, 5.43 mmol) in acetonitrile (1 mL). The reaction mixture was stirred at 100 °C for 17 h. After completion, the mixture was transferred into a separatory funnel containing saturated aqueous $NaHCO_3$ (15 mL). The aqueous layer was extracted with ethyl acetate (2 × 50 mL), and the combined organic layers were dried over anhydrous Na_2SO_4. Removal of the solvent under reduced pressure afforded the crude product, which was purified by crystallization using EtOAc/petroleum ether (1:10). Pure *N*-benzhydrylacetamide (**4a**) was obtained as a solid (1 g, 82% yield).

Raw ^{1}H NMR spectrum showing the formation of S-methyl methanesulfonothioate during the etherification of diphenylmethanol

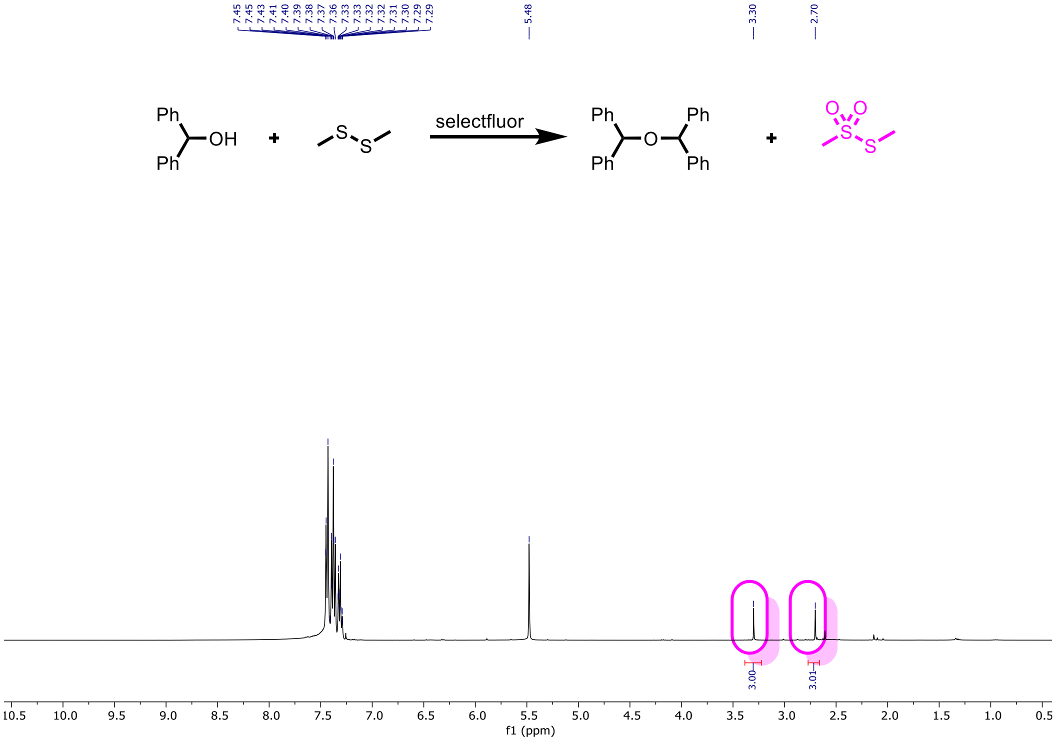

REFERENCES

1. Brahmachari, G., Banerjee, B., **2013**, "Facile Synthesis of Symmetrical bis (benzhydryl) ethers using p-Toluenesulfonyl Chloride Under Solvent-Free Conditions", Organic and medicinal chemistry letters, 3(1),1.

2. Das, T., Chakraborty, A., Sarkar, A., **2014**, "Solvent Control of Product Diversity in Palladium-Catalyzed Addition of Arylboronic acid to Aryl Aldehydes", *Tetrahedron Letters*, *55*(37), 5174-5178.

3. Singh, A. G., Alawaed, A. A., Ramachandran, P. V., **2024**, "Titanium Tetrafluoride Catalysis for the Dehydrative Conversion of Diphenylmethanols to Symmetric and Unsymmetric Ethers", *RSC advances*, 14(33), 24236-24239.

4. Wang, H., Zhu, X., Lu, Y., Li, Y., Gao, X., **2011**, Sodium Bisulfite: An Efficient Catalyst for Ether Formation via Dehydration of Aromatic/Aliphatic Alcohol", *Chinese Journal of Chemistry*, 29(6), 1180-1184.

5. Jereb, M., Vražič, D., **2013**, "Iodine-Catalyzed Disproportionation of Aryl-Substituted Ethers under Solvent-Free Reaction Conditions", *Organic & Biomolecular Chemistry*, 11(12), 1978-1999.

6. Lichtenberg, C., Bloch, J., Gianetti, T. L., Büttner, T., Geier, J., Grützmacher, H. **2015**, "Diolefins with an Ether/thioether Functionality as Ligands in the Coordination Sphere of Ni and Rh", *Dalton Transactions*, 44(46), 20056-20066.

7. Chenniappan, V. K., Silwal, S., Rahaim, R. J., **2018**, "Ni/Ti Dual Catalytic Cross-Coupling of Nitriles and Organobromides to Access Ketones. *ACS catalysis*, 8(5), 4539-4544.

8. Zhao, B., Lu, X., **2006**. "Cationic Palladium (II)-Catalyzed Addition of Arylboronic Acids to Nitriles. One-step Synthesis of Benzofurans from Phenoxyacetonitriles", *Organic Letters*, 8(26), 5987-5990.

9. Kobayashi, K., Nishimura, Y., Gao, F., Gotoh, K., Nishihara, Y., Takagi, K., **2011**, "Rh-Catalyzed Carbonylation of Arylzinc Compounds Yielding Symmetrical Diaryl Ketones by the Assistance of Oxidizing Agents", *The Journal of Organic Chemistry*, 76(6), 1949-1952.

10. Wang, X., Liu, F. D., Tu, H. Y., Zhang, A. D., **2014**, "One-pot Synthesis of Diarylmethanones Through Palladium-Catalyzed Sequential Coupling and Aerobic Oxidation of Aryl Bromides with Acetophenone as a Latent Carbonyl Donor", *The Journal of Organic Chemistry*, 79(14), 6554-6562.

11. Li, H., Yang, M., Qi, Y., Xue, J., **2011**, "Ligand-Free Pd-Catalyzed Carbonylative Cross-Coupling Reactions under Atmospheric Pressure of Carbon Monoxide: Synthesis of Aryl Ketones and Heteroaromatic Ketones", *Europen Journal of Organic Chemistry*, 2662–2667.

12. Sun, N., Sun, Q., Zhao, W., Jin, L., Hu, B., Shen, Z., Hu, X., **2019**, "Ligand-Free Palladium-Catalyzed Carbonylative Suzuki Coupling of Aryl Iodides in Aqueous CH_3CN with Sub-Stoichiometric Amount of $Mo(CO)_6$ as CO Source", *Advanced Synthesis & Catalysis*, 361(9), 2117-2123.

13. Karthikeyan, J., Parthasarathy, K., Cheng, C. H., **2011**, "Synthesis of Biarylketones and Phthalides from Organoboronic Acids and Aldehydes Catalyzed by Cobalt Complexes", *Chemical Communications*, 47(37), 10461-10463.

14. Li, G., Szostak, M., **2020**, "Kinetically Controlled, Highly Chemoselective Acylation of Functionalized Grignard Reagents with Amides by N−C Cleavage", *Chemistry–A European Journal*, 26(3), 611-615.

15. Yamada, T., Saito, K., Akiyama, T., **2016**, "Transformation of Trifluorotoluenes Triggered by Titanium (IV) Chloride-Catalyzed Hydrodefluorination using Hydrosilanes", *Advanced Synthesis & Catalysis*, 358(1), 62-66.

16. Sanz, R., Martínez, A., Guilarte, V., Álvarez-Gutiérrez, J. M., Rodríguez, F., **2007**, "The Ritter Reaction under Truly Catalytic Brønsted Acid Conditions", *Europen Journal of Organic Chemistry*, 2007, 4642–4645.

17. Crampton, R., Woodward, S., Fox, M., **2011**, "Bis-Sulfamyl Imines: Potent Substrates for Asymmetric Additions of Arylboroxines under Rhodium Catalysis", *Advanced Synthesis & Catalysis*, 353(6), 903-906.

18. Barbero, M., Bazzi, S., Cadamuro, S., Dughera, S., **2009**, "o-Benzenedisulfonimide as a Reusable Brønsted Acid Catalyst for Ritter-Type Reactions", *Europen Journal of Organic Chemistry*, 430–436.

19. Zhang, Y., Dong, J., Liu, L., Liu, L., Zhou, Y., Yin, S. F., **2017**, "Manganese (III) Acetate Catalyzed Oxidative Amination of Benzylic $C(sp^3)$–H Bonds with Nitriles", *Organic & Biomolecular Chemistry*, 15(14), 2897-2901.

20. Liu, X., Xia, Q., Zhang, Y., Chen, C., Chen, W., **2013**, "Cu-NHC-TEMPO Catalyzed Aerobic Oxidation of Primary Alcohols to Aldehydes", *The Journal of Organic Chemistry*, 78(17), 8531-8536.

NMR Spectra

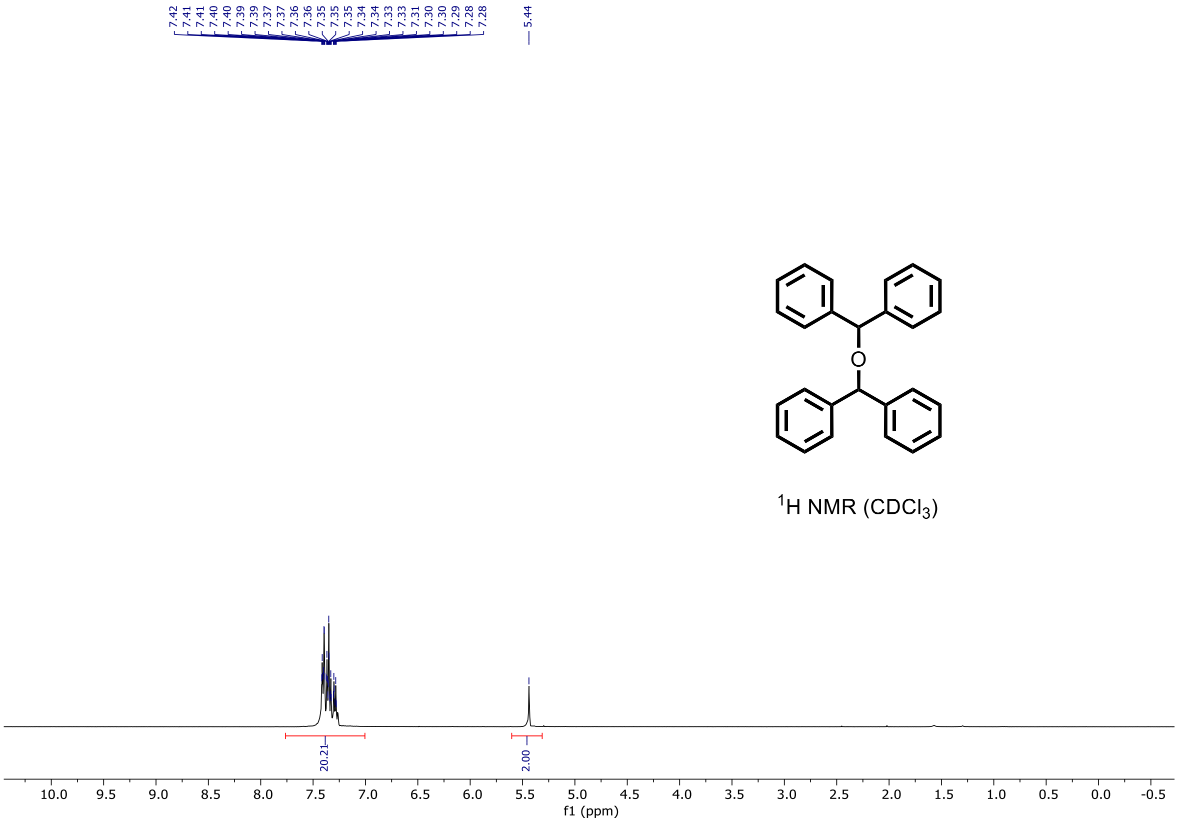
7.42
7.41
7.41
7.40
7.40
7.39
7.39
7.37
7.37
7.36
7.36
7.35
7.35
7.35
7.34
7.34
7.33
7.33
7.31
7.30
7.30
7.29
7.28
7.28
5.44
^{1}H NMR ($CDCl_3$)
20.21
2.00
10.0 9.5 9.0 8.5 8.0 7.5 7.0 6.5 6.0 5.5 5.0 4.5 4.0 3.5 3.0 2.5 2.0 1.5 1.0 0.5 0.0 -0.5
f1 (ppm)

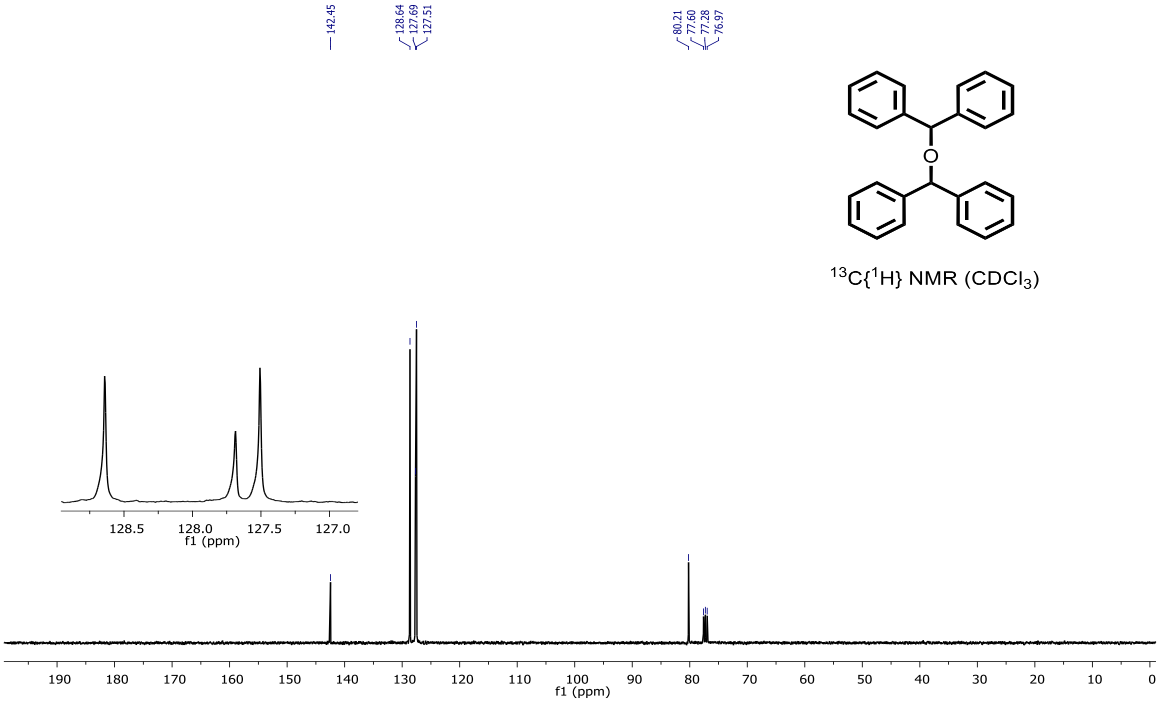
142.45
128.64
127.69
127.51
80.21
77.60
77.28
76.97
$^{13}C\{^{1}H\}$ NMR ($CDCl_3$)
128.5 128.0 127.5 127.0
f1 (ppm)
190 180 170 160 150 140 130 120 110 100 90 80 70 60 50 40 30 20 10 0
f1 (ppm)

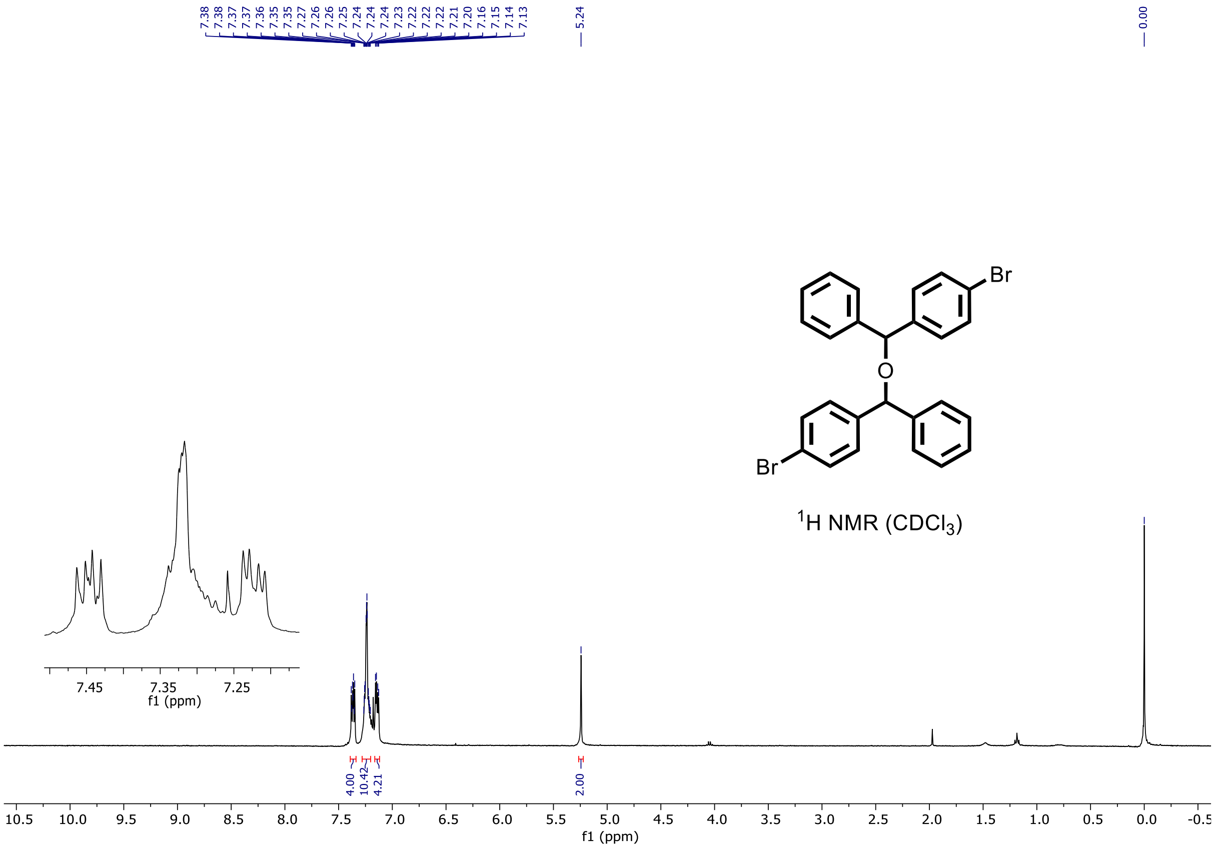
Br
O
Br
^{1}H NMR ($CDCl_3$)
f1 (ppm)

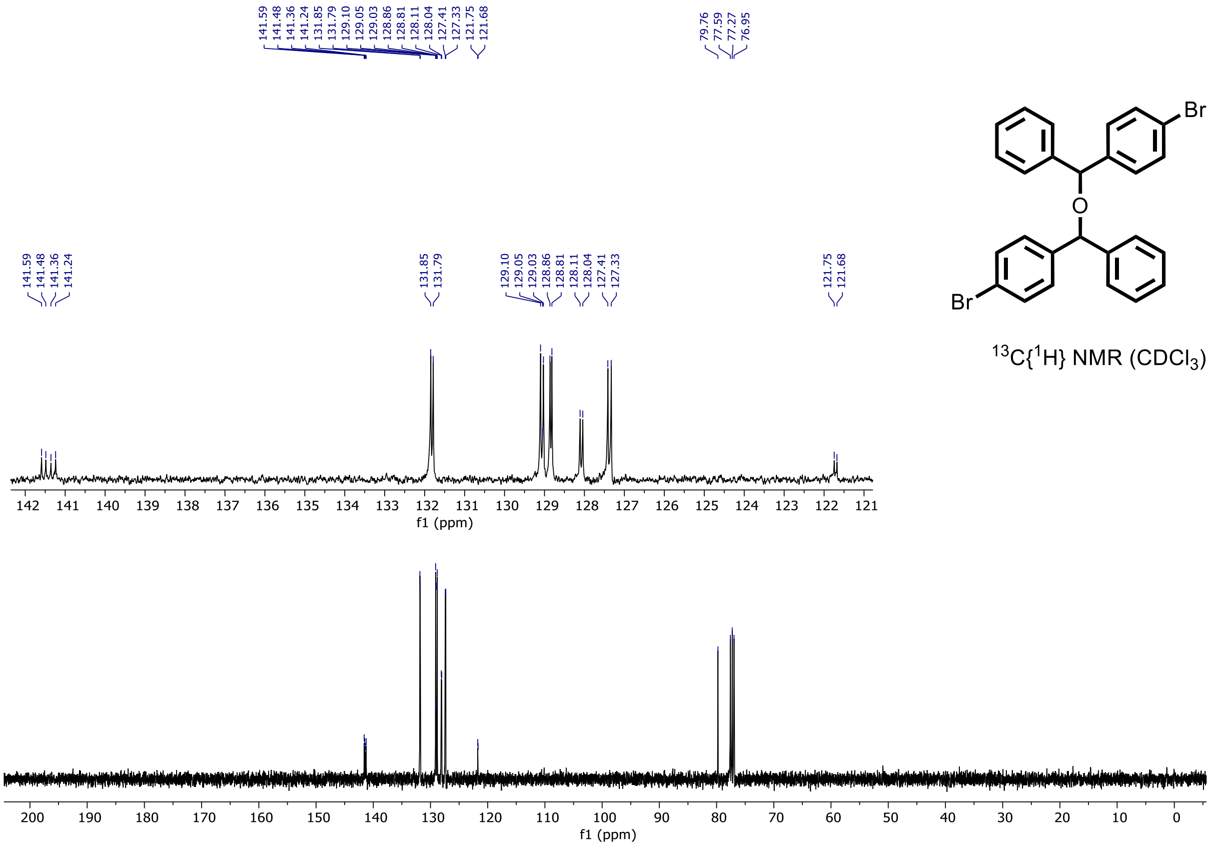
Br
O
Br
^{13}C{^{1}H} NMR ($CDCl_3$)
f1 (ppm)

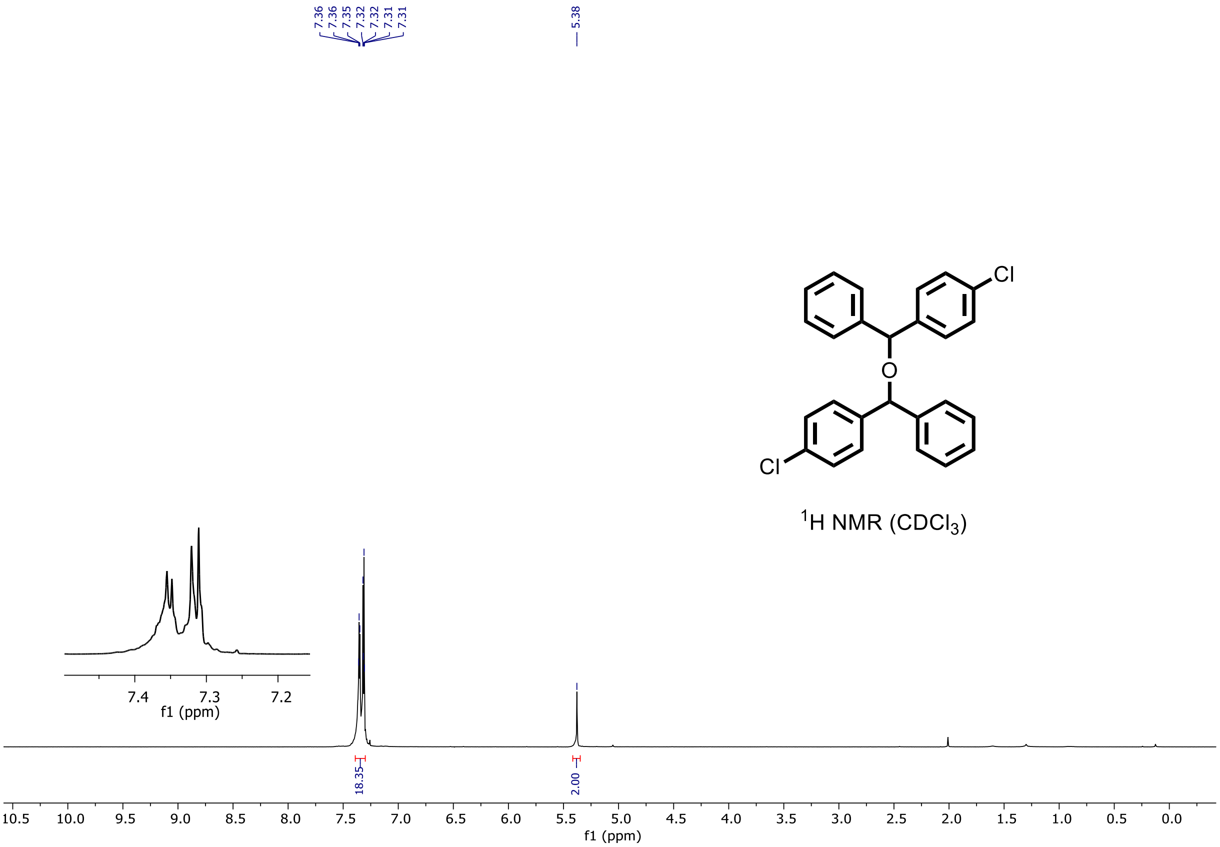

7.36
7.36
7.35
7.32
7.32
7.31
7.31
5.38
Cl
O
Cl
^{1}H NMR (CDCl$_3$)
18.35
2.00
f1 (ppm)

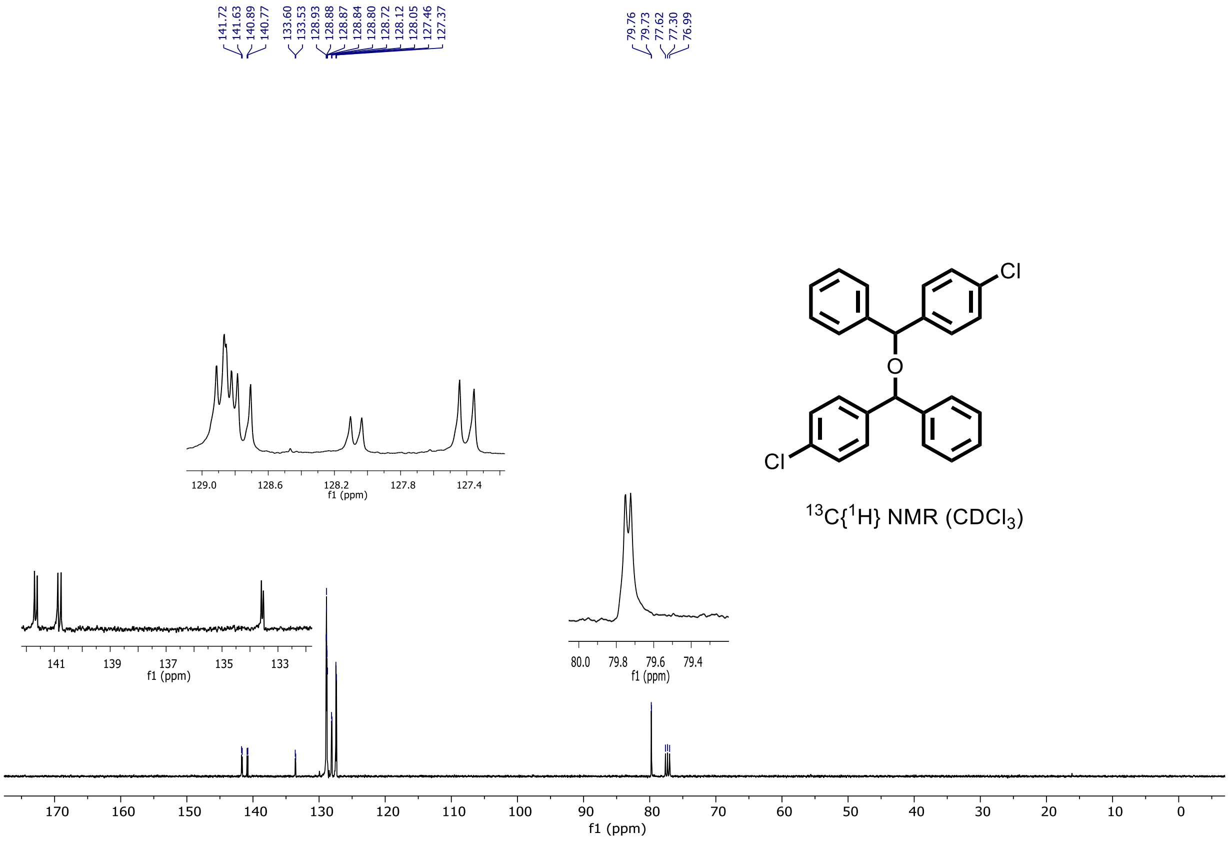

141.72
141.63
140.89
140.77
133.60
133.53
128.93
128.88
128.87
128.84
128.80
128.72
128.12
128.05
127.46
127.37
79.76
79.73
77.62
77.30
76.99
Cl
O
Cl
^{13}C{^{1}H} NMR (CDCl$_3$)
f1 (ppm)

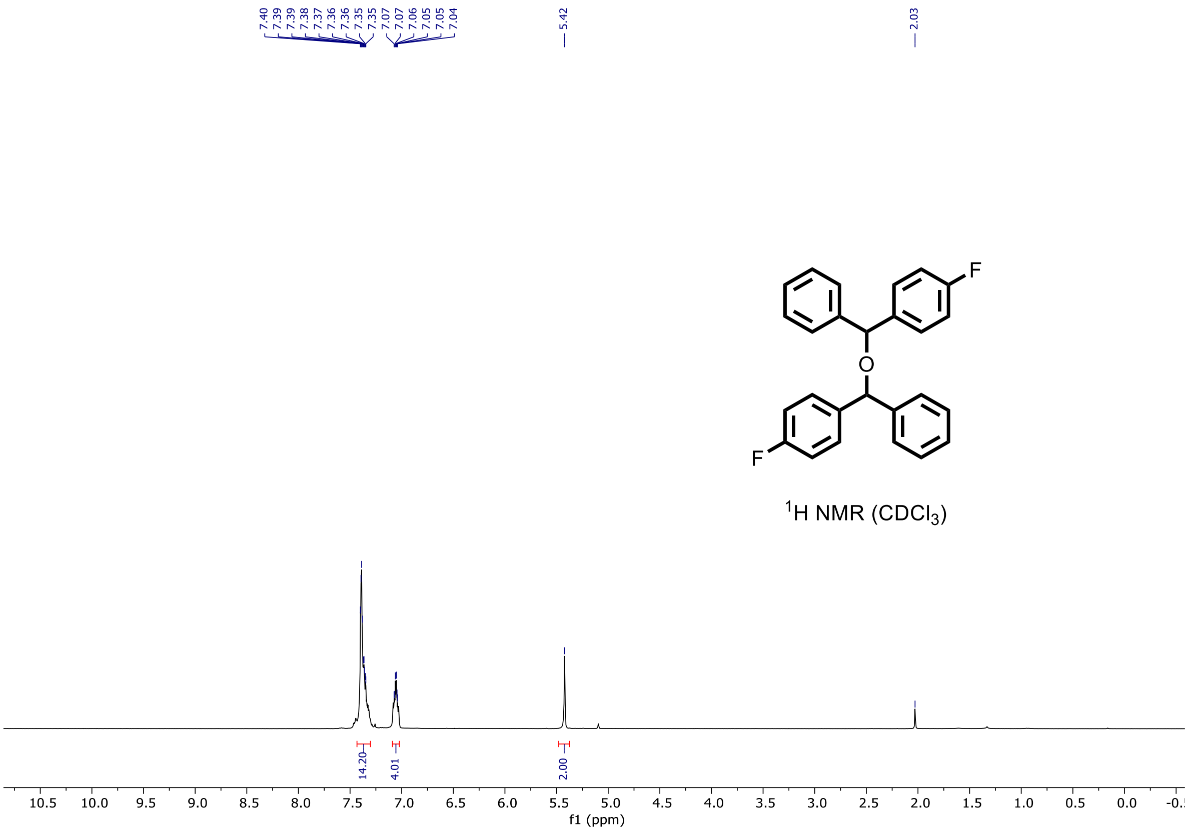
7.40
7.39
7.39
7.38
7.37
7.36
7.36
7.35
7.35
7.07
7.07
7.06
7.05
7.05
7.04
5.42
2.03
F
O
F
^{1}H NMR (CDCl$_3$)
14.20
4.01
2.00
10.5 10.0 9.5 9.0 8.5 8.0 7.5 7.0 6.5 6.0 5.5 5.0 4.5 4.0 3.5 3.0 2.5 2.0 1.5 1.0 0.5 0.0
f1 (ppm)

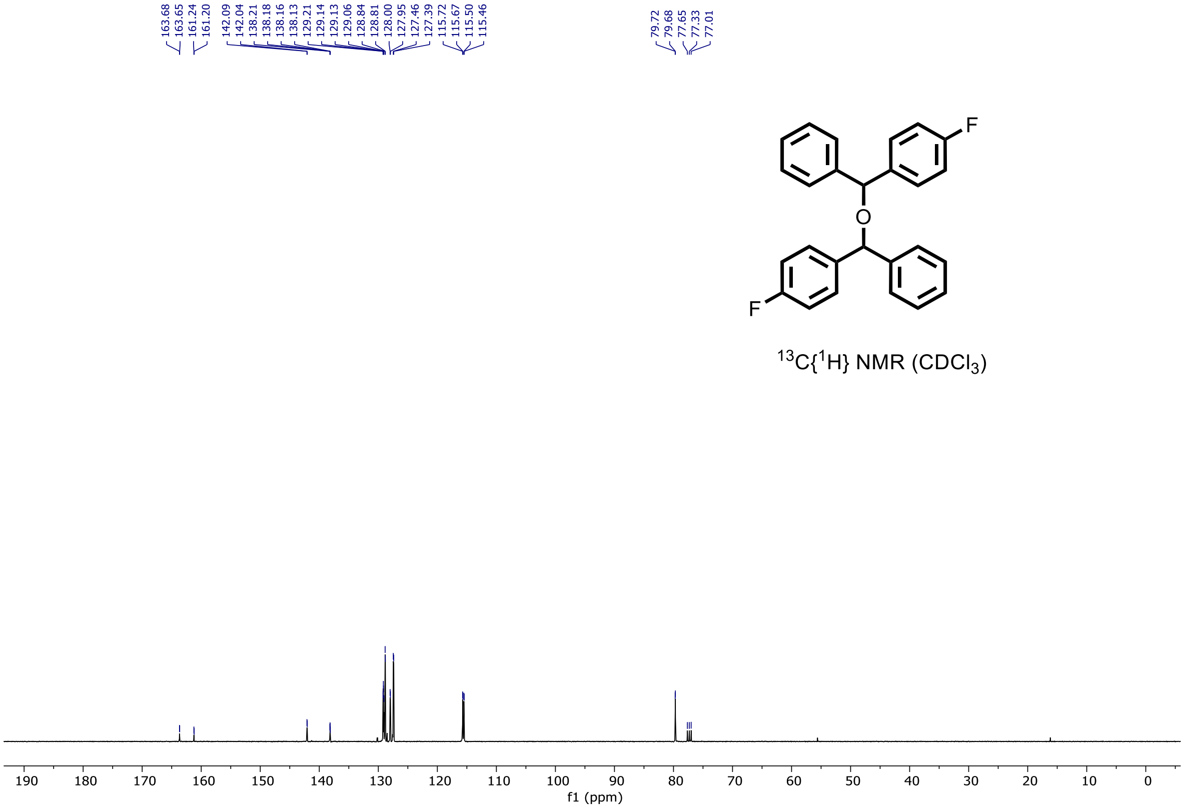
163.68
163.65
161.24
161.20
142.09
142.04
138.21
138.18
138.16
138.13
129.21
129.14
129.13
129.06
128.84
128.81
128.00
127.95
127.46
127.39
115.72
115.67
115.50
115.46
79.72
79.68
77.65
77.33
77.01
F
O
F
^{13}C{^{1}H} NMR (CDCl$_3$)
190 180 170 160 150 140 130 120 110 100 90 80 70 60 50 40 30 20 10 0
f1 (ppm)

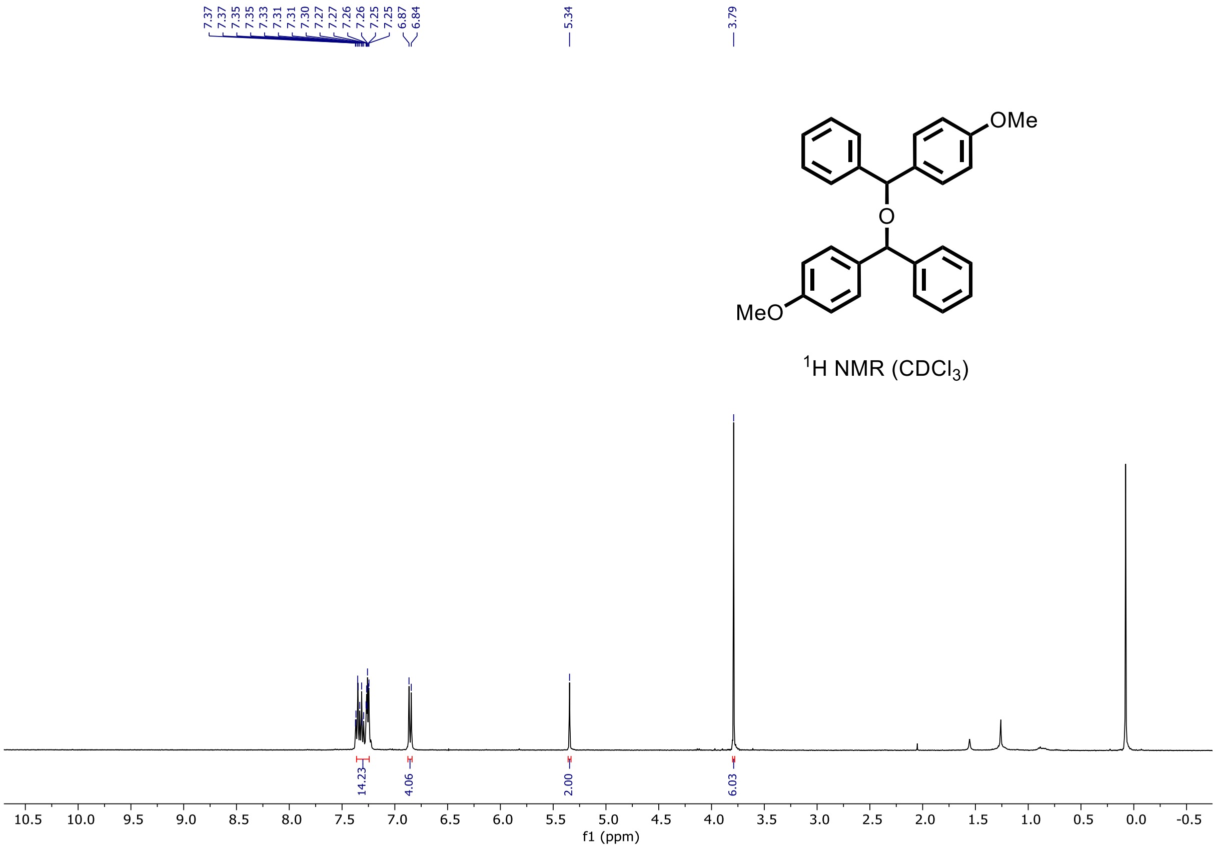
OMe
O
MeO
^{1}H NMR ($CDCl_3$)
f1 (ppm)

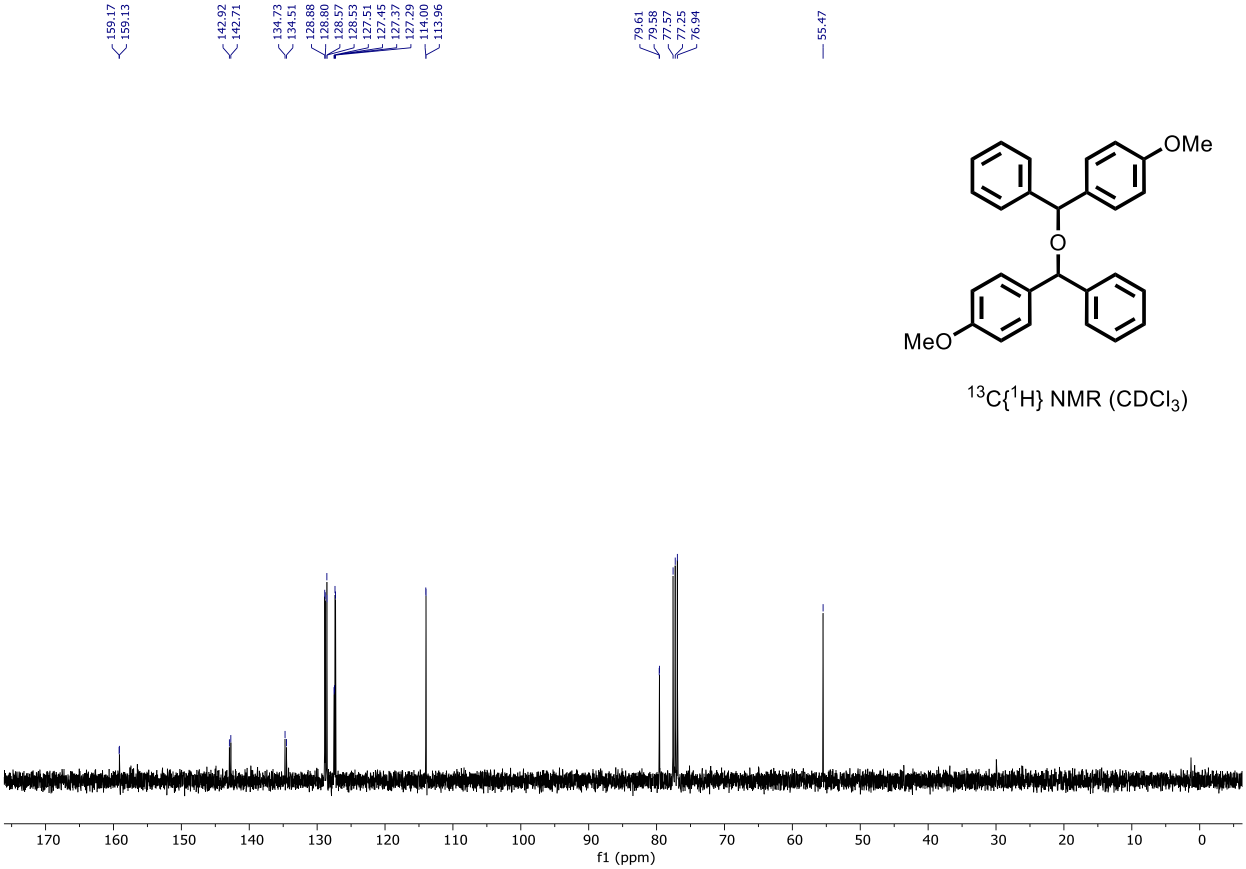
OMe
O
MeO
^{13}C{^{1}H} NMR ($CDCl_3$)
f1 (ppm)

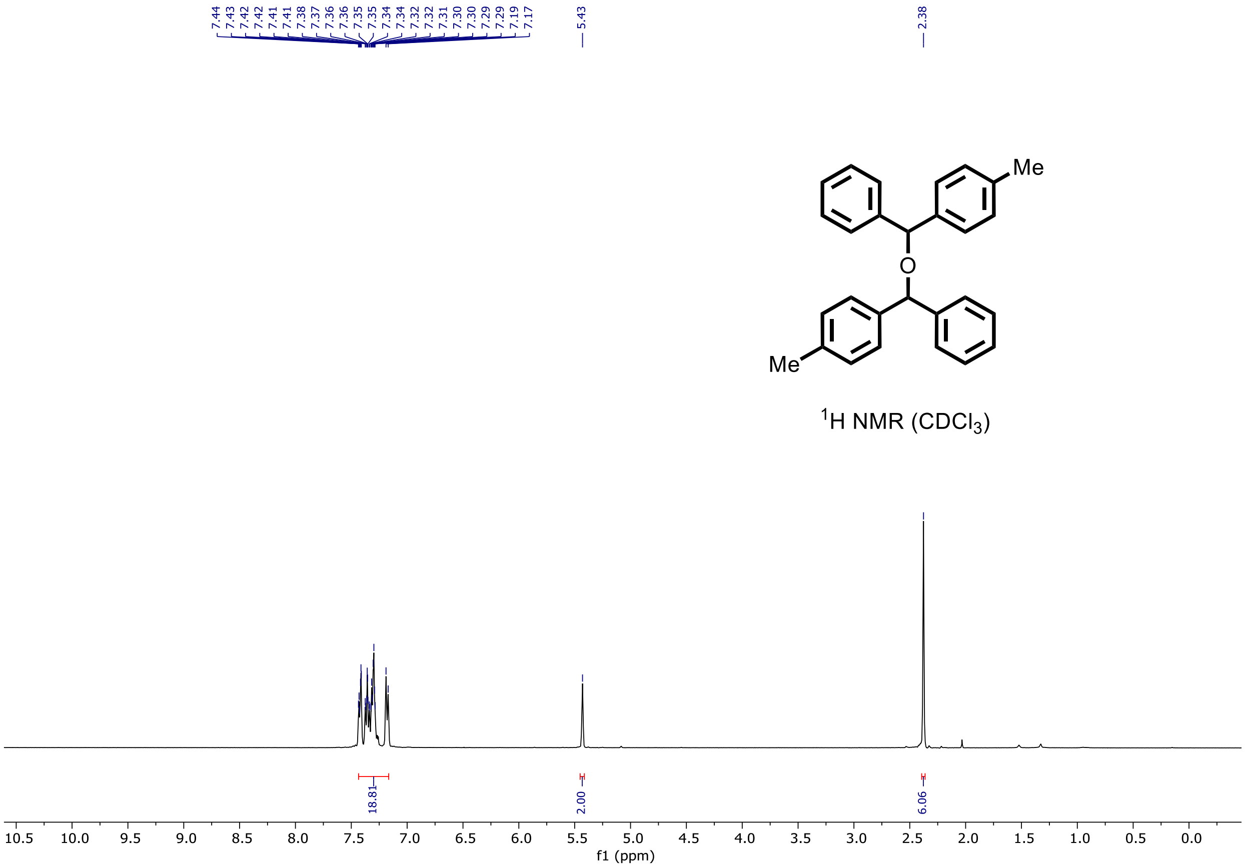

7.44
7.43
7.42
7.42
7.41
7.41
7.38
7.37
7.36
7.36
7.35
7.35
7.34
7.34
7.32
7.32
7.31
7.30
7.30
7.29
7.29
7.19
7.17
5.43
2.38
Me
O
Me
^{1}H NMR (CDCl$_3$)
18.81
2.00
6.06
10.5
10.0
9.5
9.0
8.5
8.0
7.5
7.0
6.5
6.0
5.5
5.0
4.5
4.0
3.5
3.0
2.5
2.0
1.5
1.0
0.5
0.0
f1 (ppm)

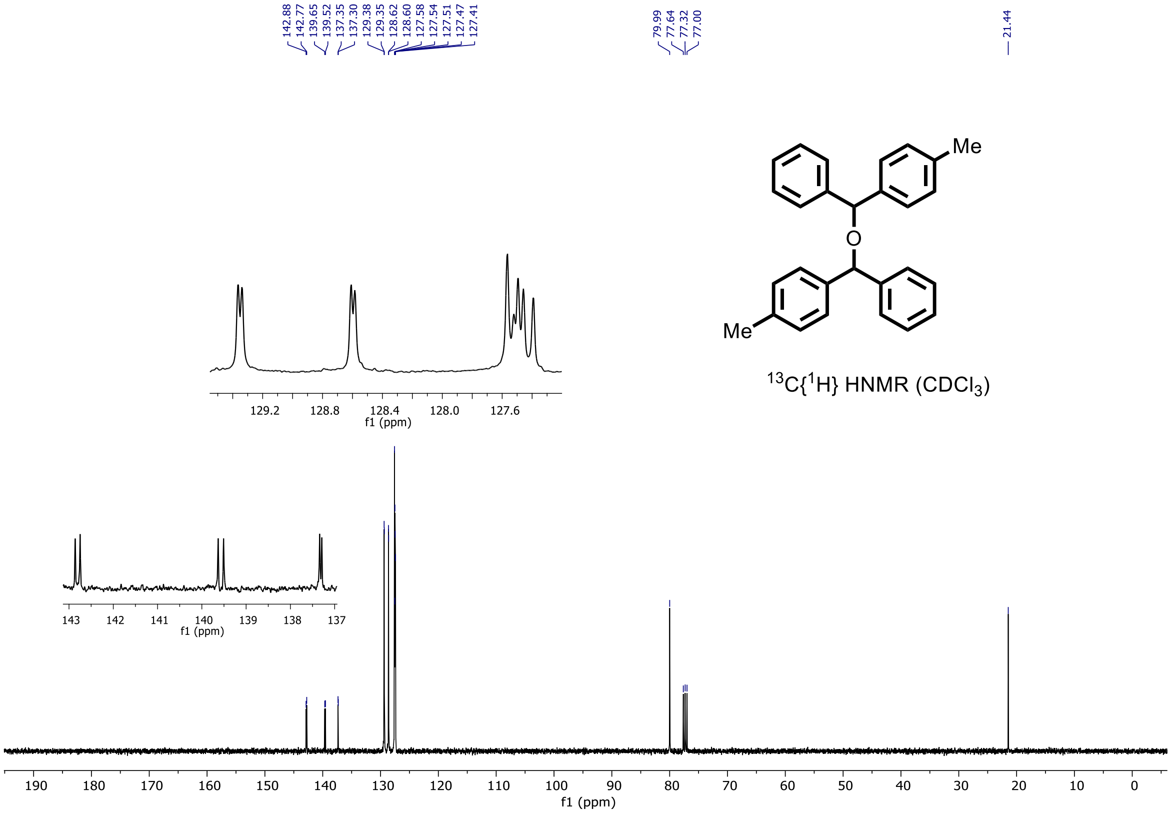

142.88
142.77
139.65
139.52
137.35
137.30
129.38
129.35
128.62
128.60
127.58
127.54
127.51
127.47
127.41
79.99
77.64
77.32
77.00
21.44
Me
O
Me
^{13}C{^{1}H} HNMR (CDCl$_3$)
129.2
128.8
128.4
128.0
127.6
f1 (ppm)
143
142
141
140
139
138
137
f1 (ppm)
190
180
170
160
150
140
130
120
110
100
90
80
70
60
50
40
30
20
10
0
f1 (ppm)

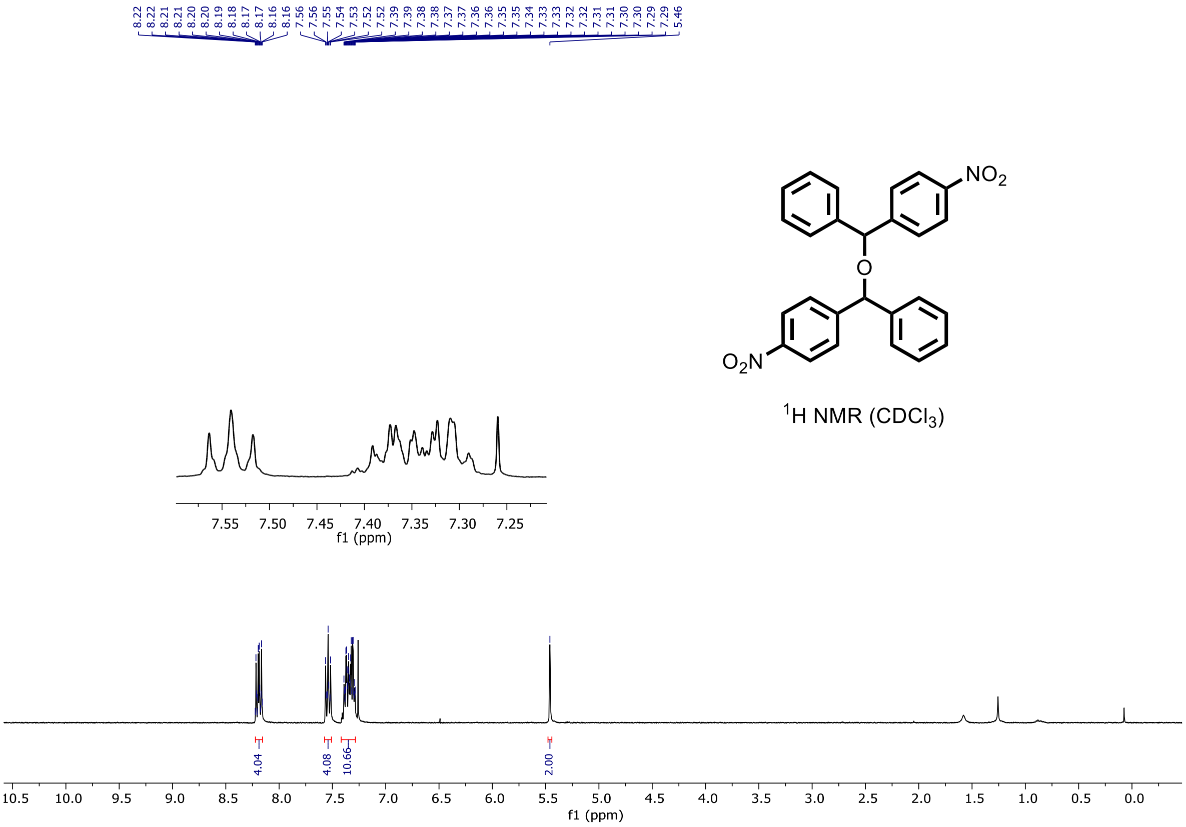

^{1}H NMR ($CDCl_3$)

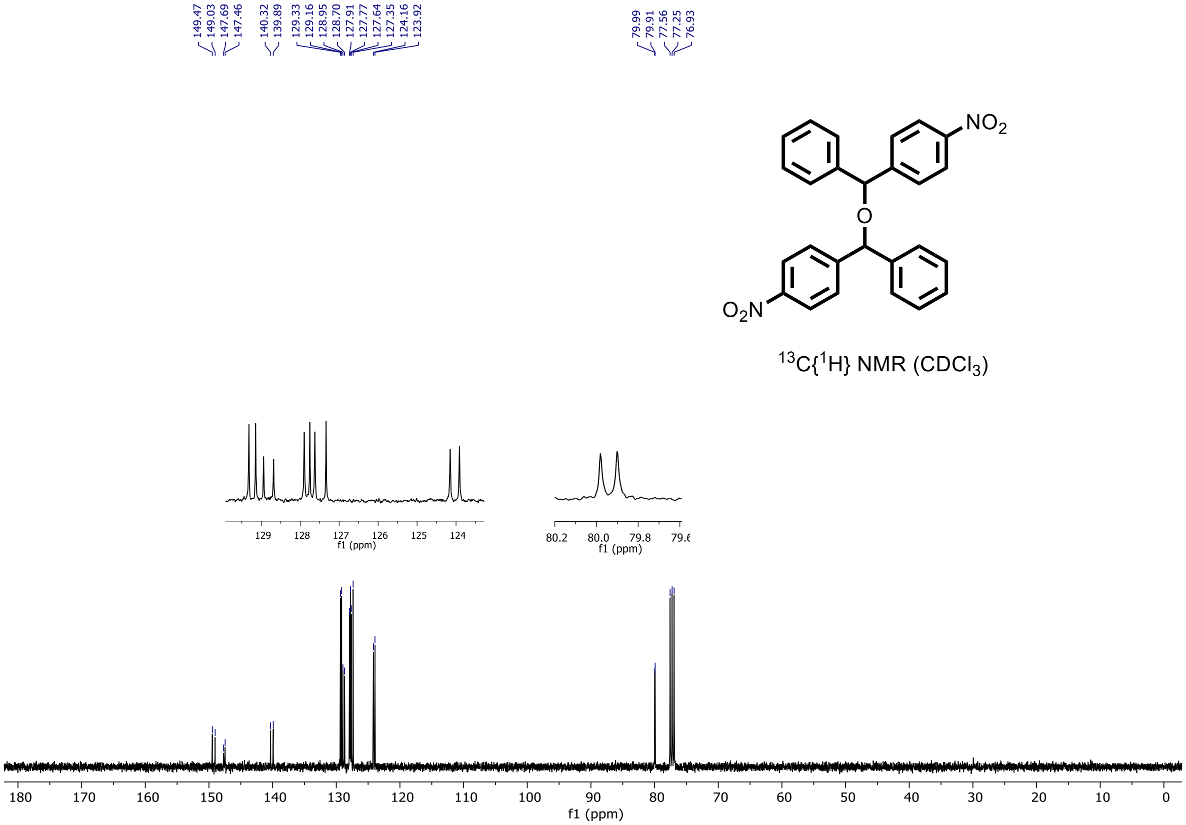

$^{13}C\{^{1}H\}$ NMR ($CDCl_3$)

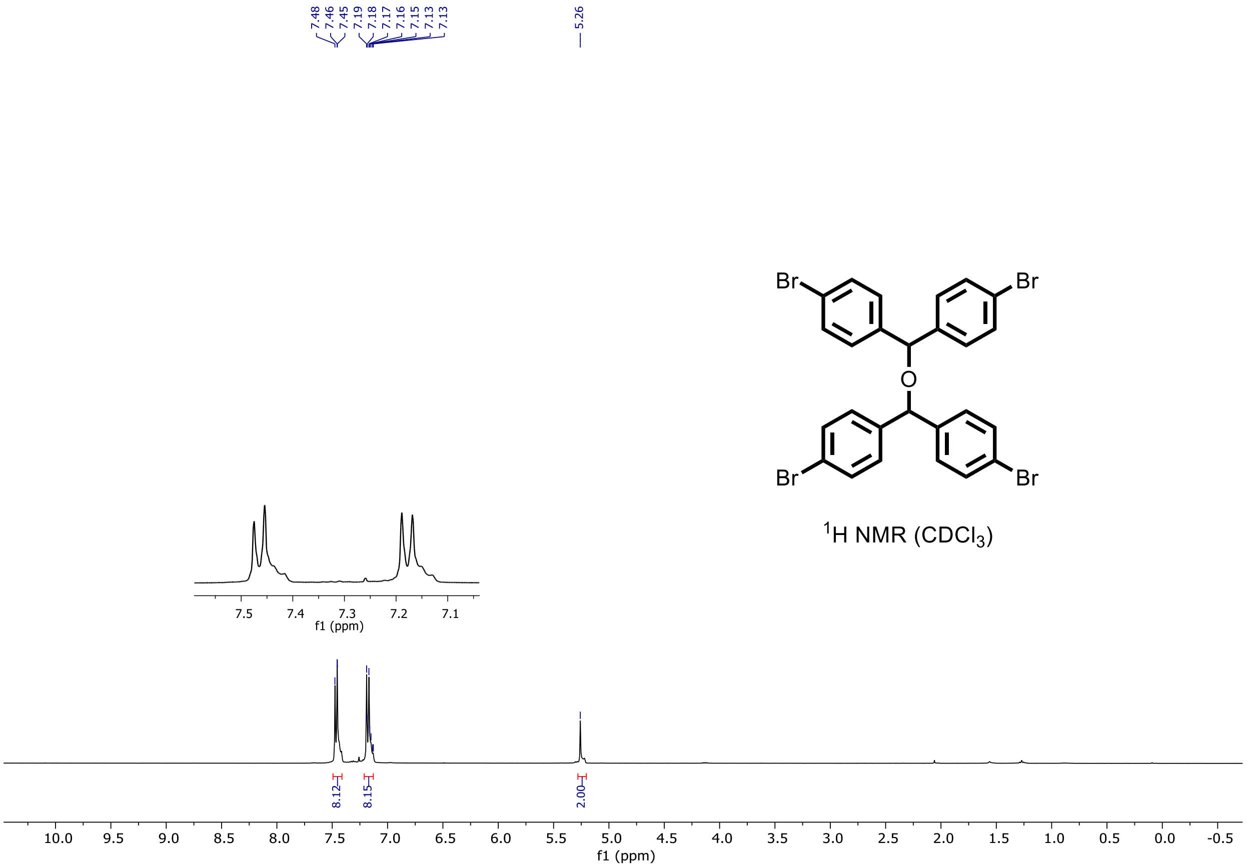
7.48
7.46
7.45
7.19
7.18
7.17
7.16
7.15
7.13
7.13
5.26
Br
Br
O
Br
Br
1H NMR (CDCl3)
7.5
7.4
7.3
7.2
7.1
f1 (ppm)
8.12
8.15
2.00
10.0
9.5
9.0
8.5
8.0
7.5
7.0
6.5
6.0
5.5
5.0
4.5
4.0
3.5
3.0
2.5
2.0
1.5
1.0
0.5
0.0
-0.5
f1 (ppm)

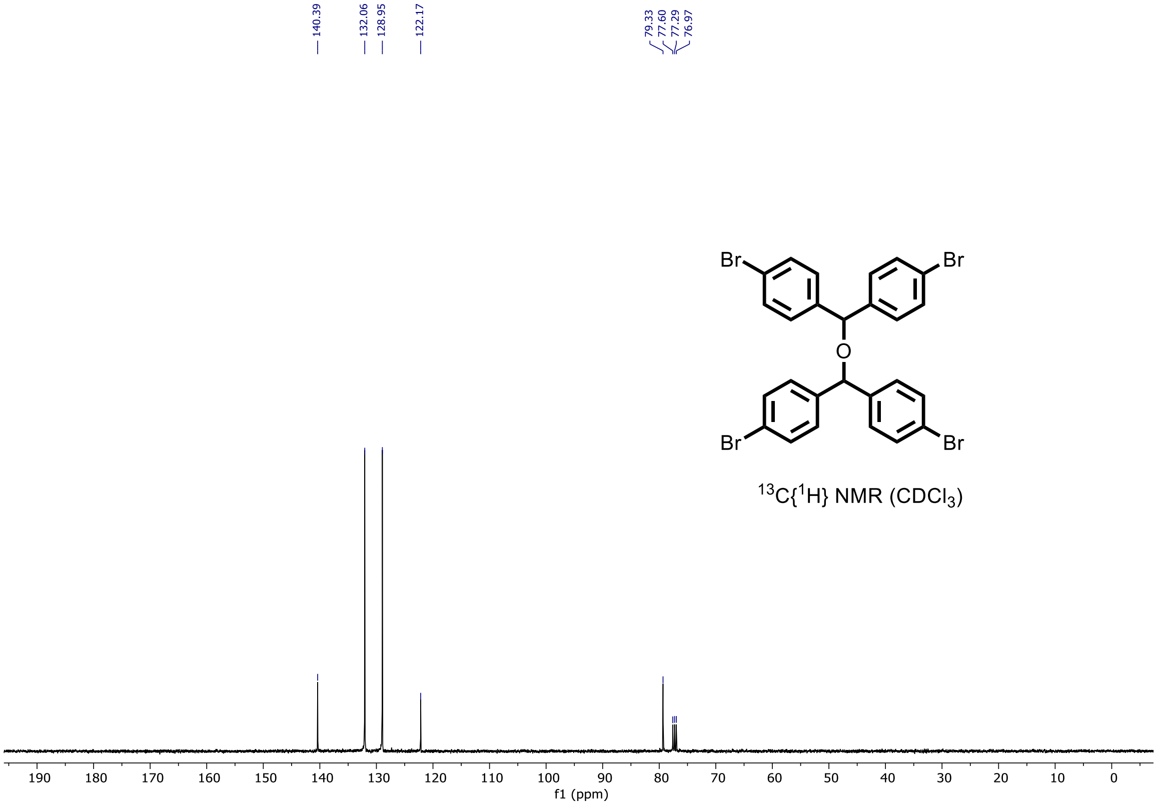
140.39
132.06
128.95
122.17
79.33
77.60
77.29
76.97
Br
Br
O
Br
Br
13C{1H} NMR (CDCl3)
190
180
170
160
150
140
130
120
110
100
90
80
70
60
50
40
30
20
10
0
f1 (ppm)

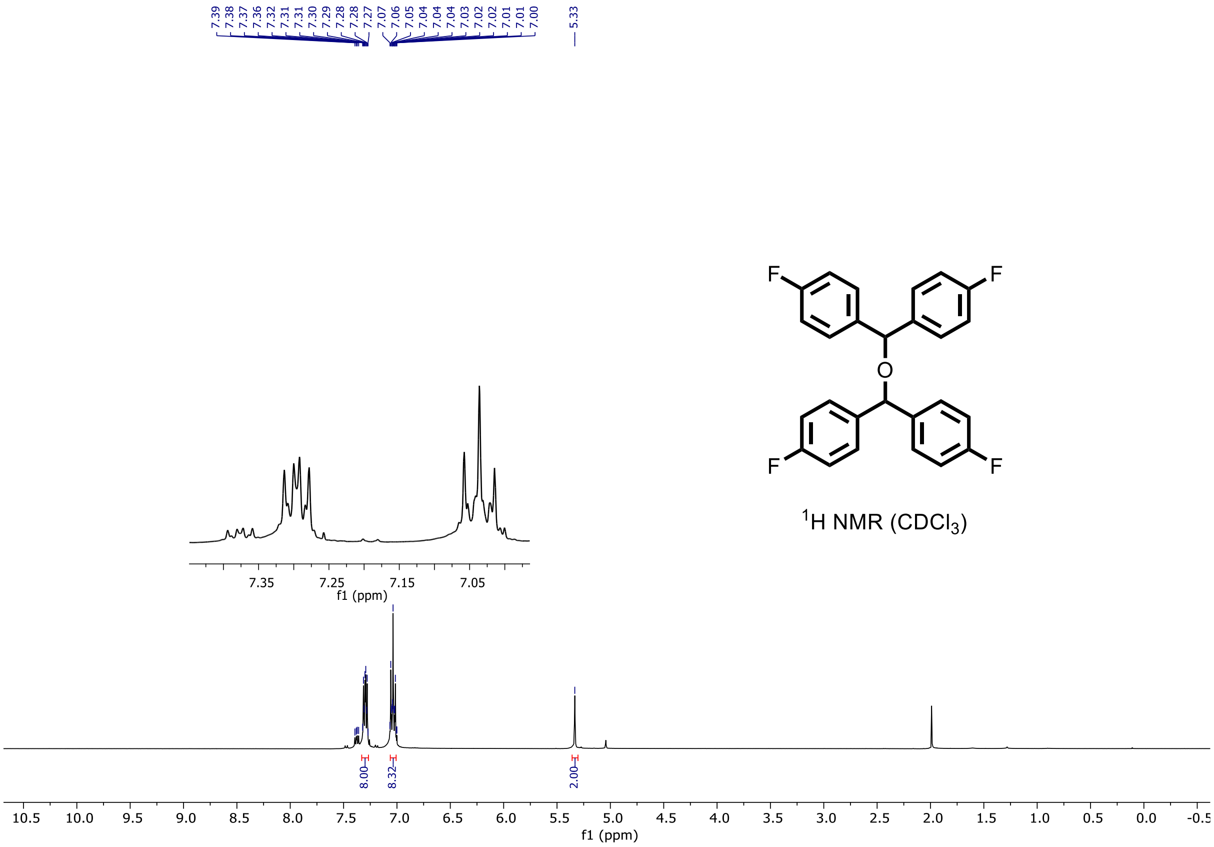
7.39
7.38
7.37
7.36
7.32
7.31
7.31
7.30
7.29
7.28
7.28
7.27
7.07
7.06
7.05
7.04
7.04
7.03
7.02
7.02
7.01
7.01
7.00
5.33
F
F
O
F
F
^{1}H NMR (CDCl$_3$)
7.35
7.25
7.15
7.05
f1 (ppm)
8.00
8.32
2.00
10.5
10.0
9.5
9.0
8.5
8.0
7.5
7.0
6.5
6.0
5.5
5.0
4.5
4.0
3.5
3.0
2.5
2.0
1.5
1.0
0.5
0.0
-0.5
f1 (ppm)

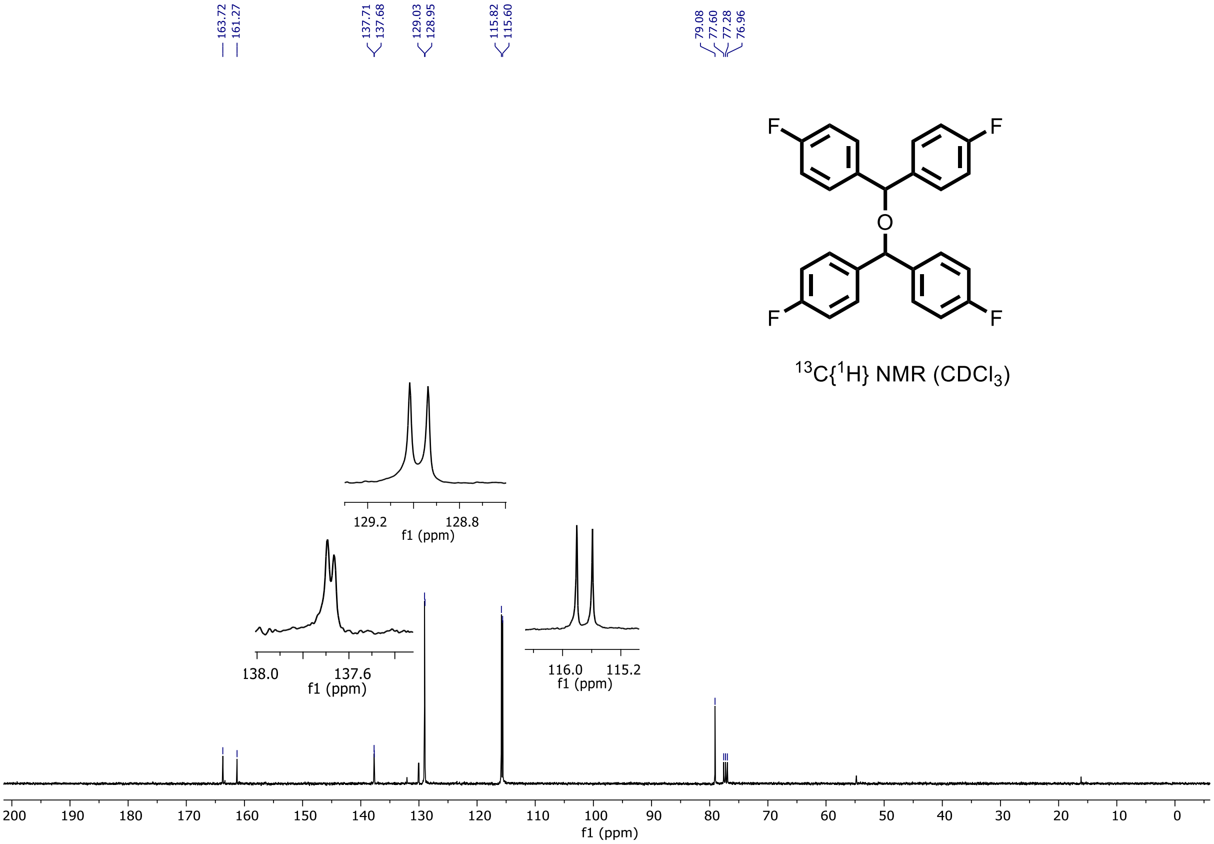
163.72
161.27
137.71
137.68
129.03
128.95
115.82
115.60
79.08
77.60
77.28
76.96
F
F
O
F
F
^{13}C{^{1}H} NMR (CDCl$_3$)
129.2
128.8
f1 (ppm)
138.0
137.6
f1 (ppm)
116.0
115.2
f1 (ppm)
200
190
180
170
160
150
140
130
120
110
100
90
80
70
60
50
40
30
20
10
0
f1 (ppm)

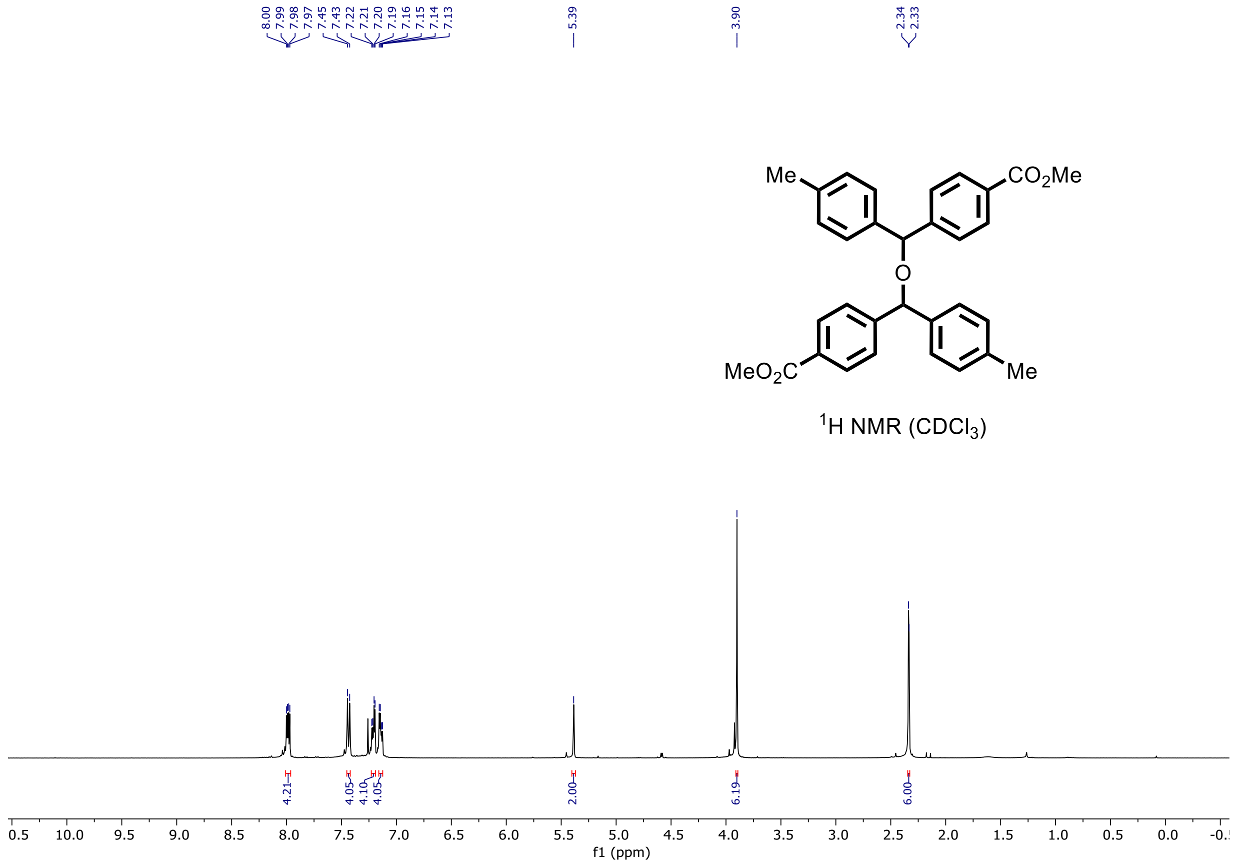
8.00 7.99 7.98 7.97 7.45 7.43 7.22 7.21 7.20 7.19 7.16 7.15 7.14 7.13
5.39
3.90
2.34 2.33
Me
CO2Me
O
MeO2C
Me
1H NMR (CDCl3)
4.21
4.05
4.10
4.05
2.00
6.19
6.00
f1 (ppm)

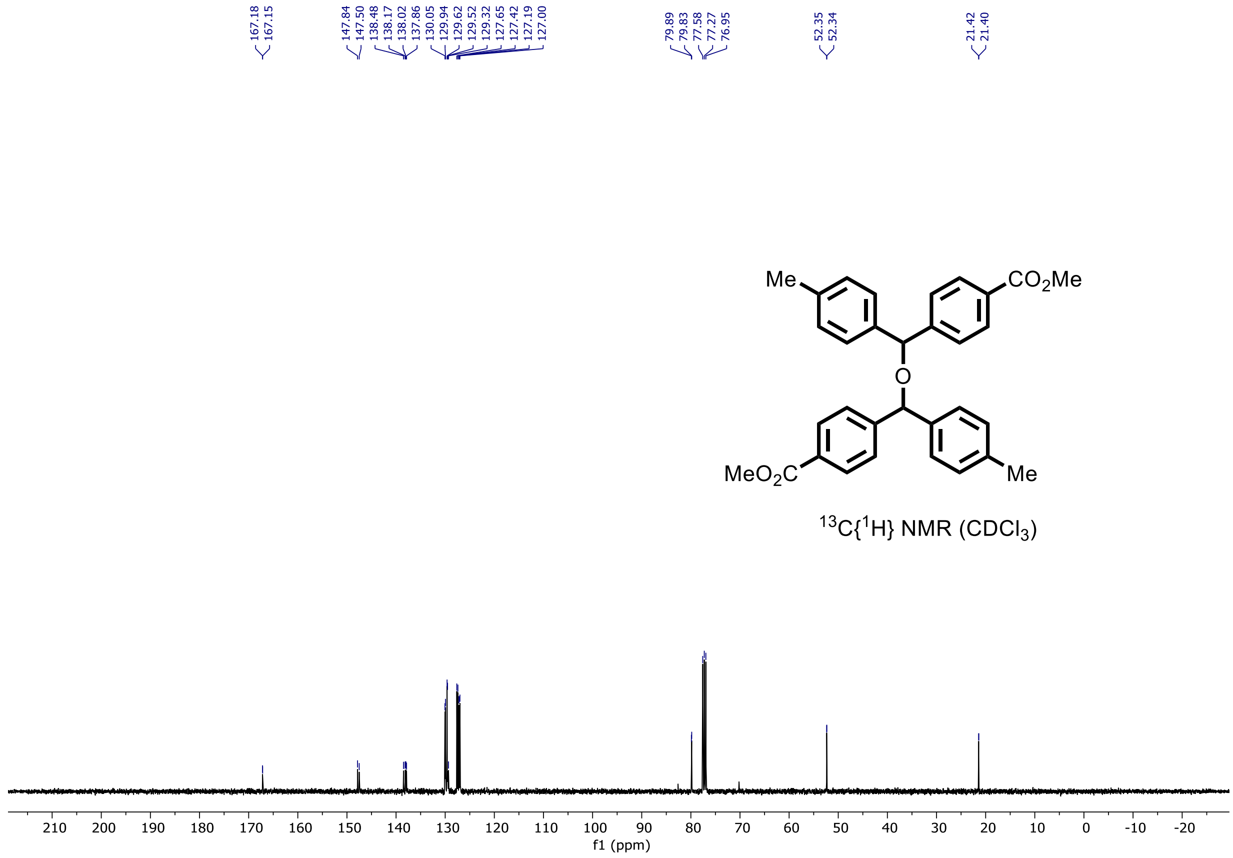
167.18 167.15
147.84 147.50 138.48 138.17 138.02 137.86 130.05 129.94 129.62 129.52 129.32 127.65 127.42 127.19 127.00
79.89 79.83 77.58 77.27 76.95
52.35 52.34
21.42 21.40
Me
CO2Me
O
MeO2C
Me
13C{1H} NMR (CDCl3)
f1 (ppm)

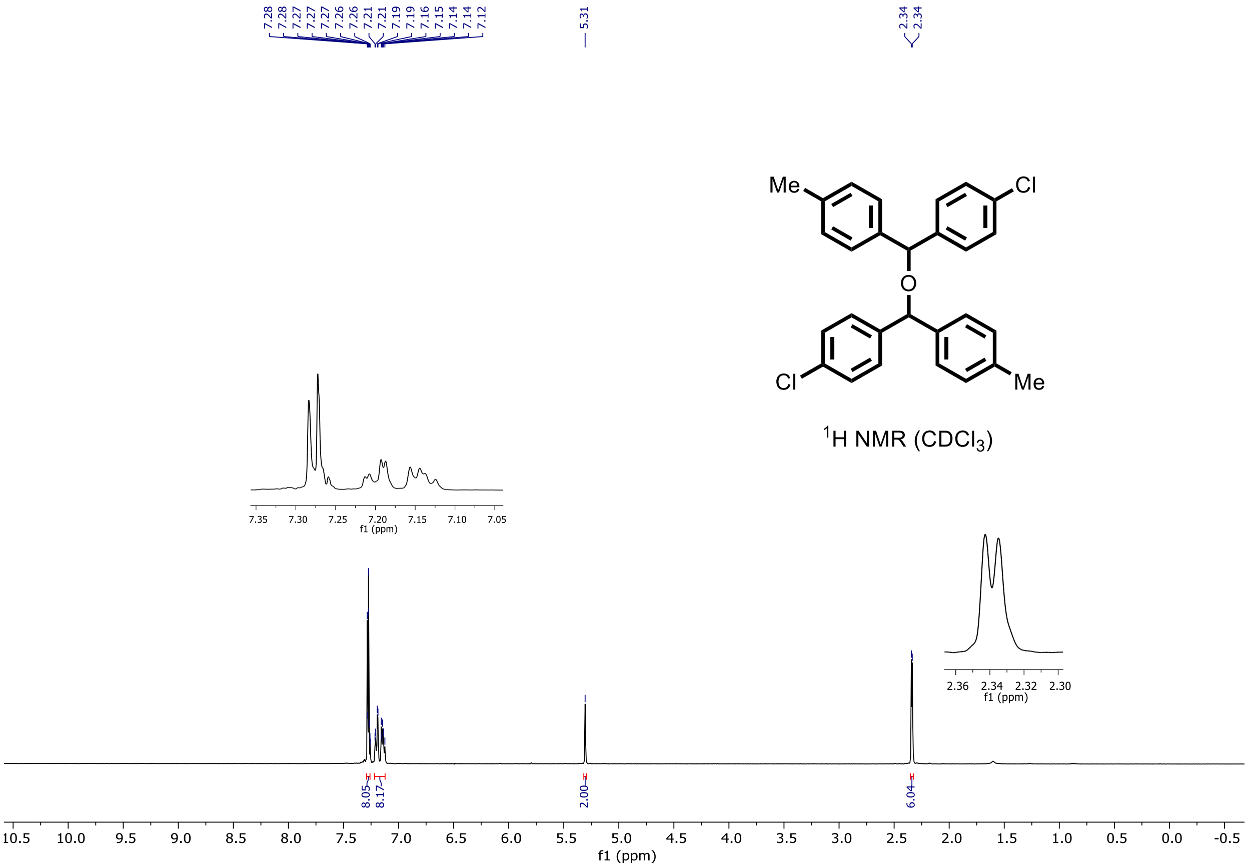
7.28 7.28 7.27 7.27 7.27 7.26 7.26 7.21 7.21 7.21 7.19 7.19 7.16 7.15 7.14 7.14 7.12
5.31
2.34 2.34
Me
Cl
O
Cl
Me
^{1}H NMR (CDCl$_3$)
8.05
8.17
2.00
6.04
f1 (ppm)

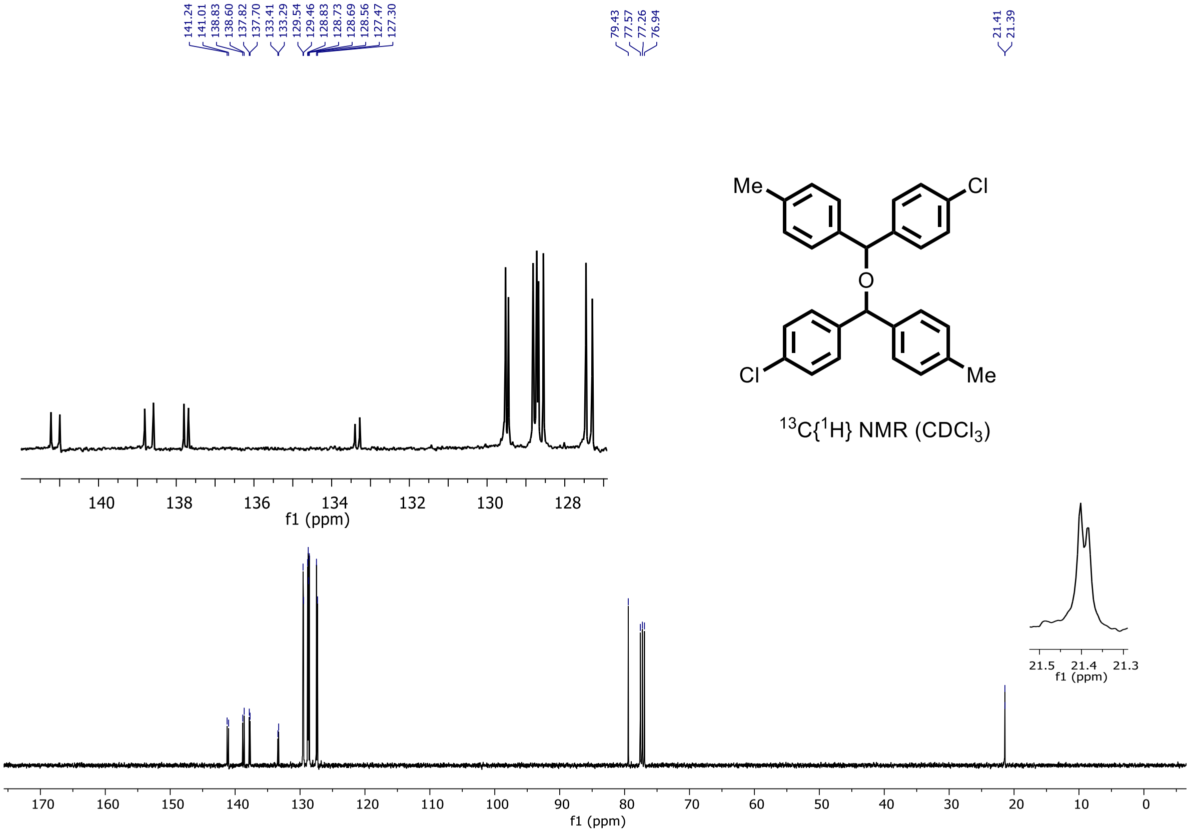
141.24 141.01 138.83 138.60 137.82 137.70 133.41 133.29 129.54 129.46 128.83 128.73 128.69 128.56 127.47 127.30
79.43 77.57 77.26 76.94
21.41 21.39
Me
Cl
O
Cl
Me
^{13}C{^{1}H} NMR (CDCl$_3$)
f1 (ppm)

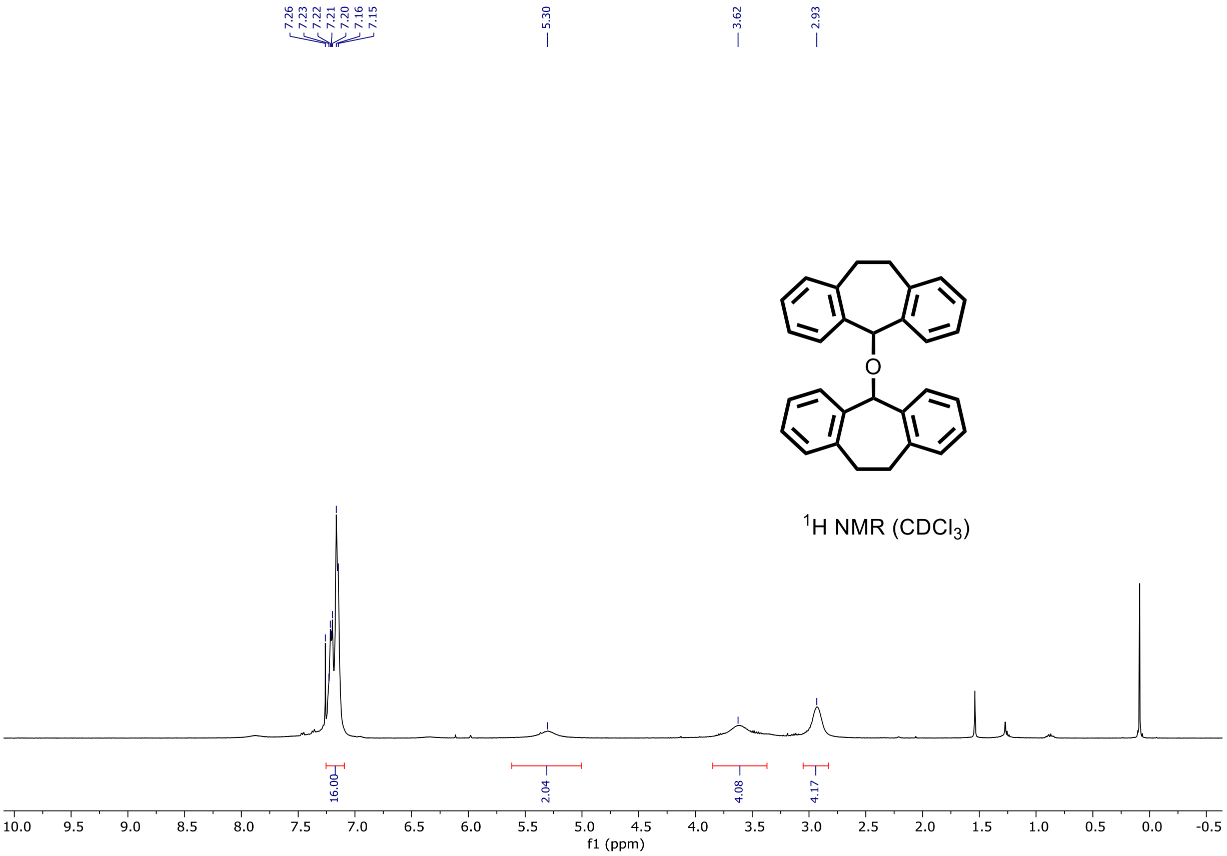

7.26
7.23
7.22
7.21
7.20
7.16
7.15
5.30
3.62
2.93
O
1H NMR (CDCl3)
16.00
2.04
4.08
4.17
10.0 9.5 9.0 8.5 8.0 7.5 7.0 6.5 6.0 5.5 5.0 4.5 4.0 3.5 3.0 2.5 2.0 1.5 1.0 0.5 0.0 -0.5
f1 (ppm)

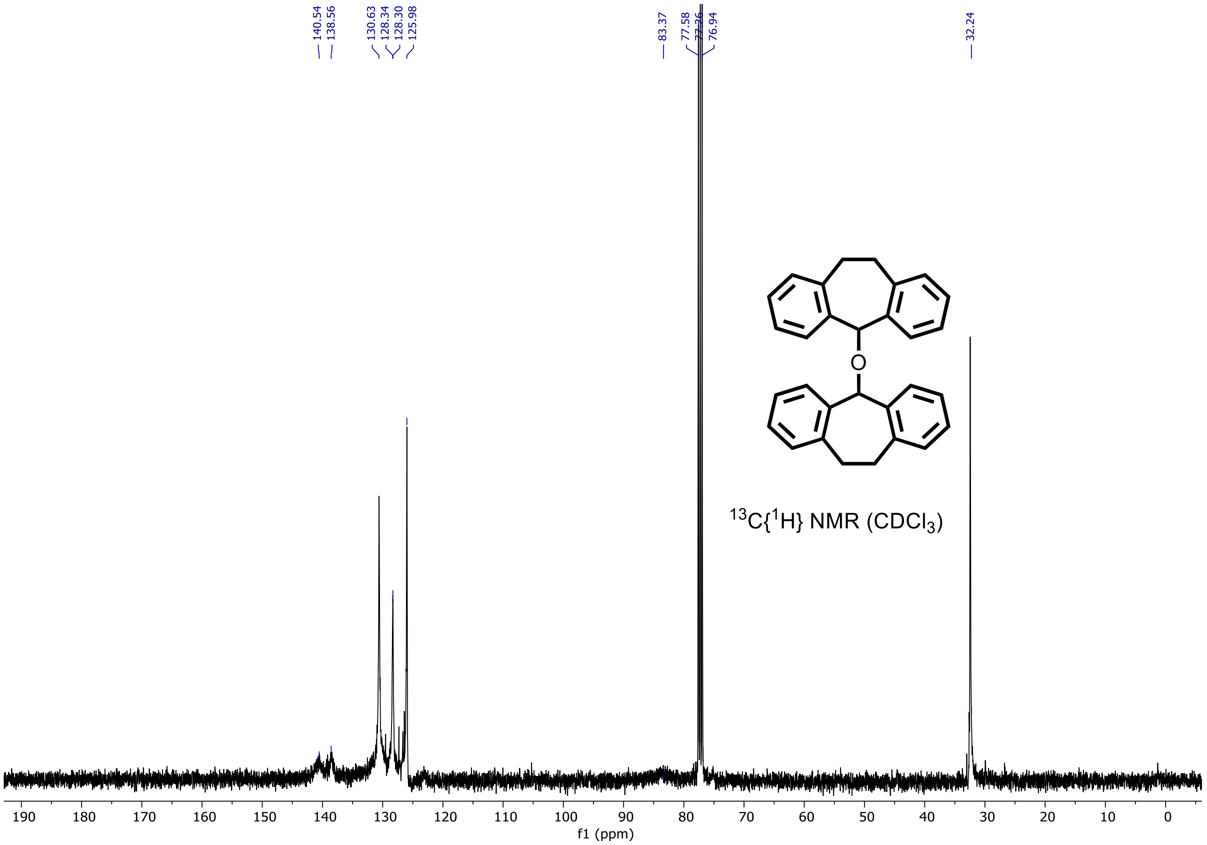

140.54
138.56
130.63
128.34
128.30
125.98
83.37
77.58
76.94
32.24
O
13C{1H} NMR (CDCl3)
190 180 170 160 150 140 130 120 110 100 90 80 70 60 50 40 30 20 10 0
f1 (ppm)

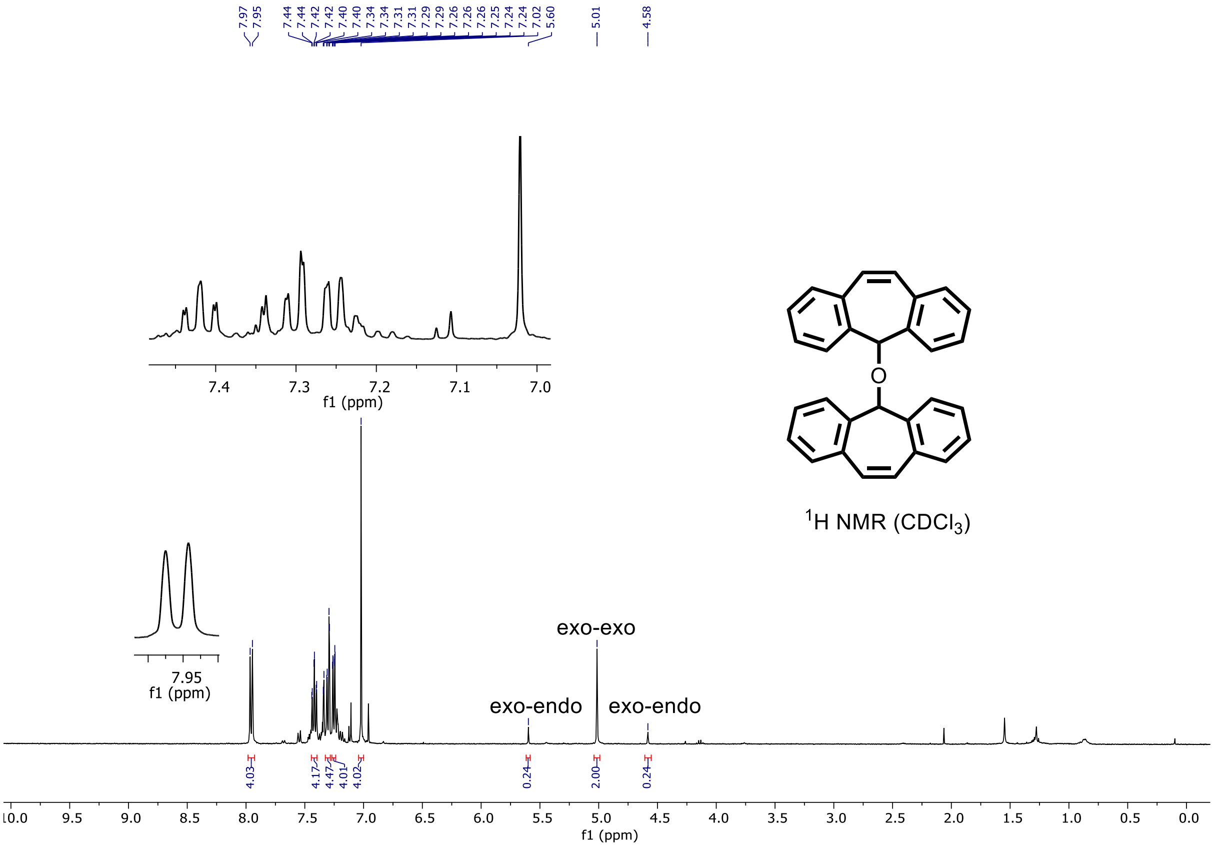
7.97
7.95
7.44
7.44
7.42
7.42
7.40
7.40
7.34
7.34
7.31
7.31
7.29
7.29
7.26
7.26
7.25
7.24
7.24
7.02
5.60
5.01
4.58
O
^{1}H NMR ($CDCl_3$)
exo-exo
exo-endo
exo-endo
7.95
f1 (ppm)
4.03
4.17
4.47
4.01
4.02
0.24
2.00
0.24
f1 (ppm)

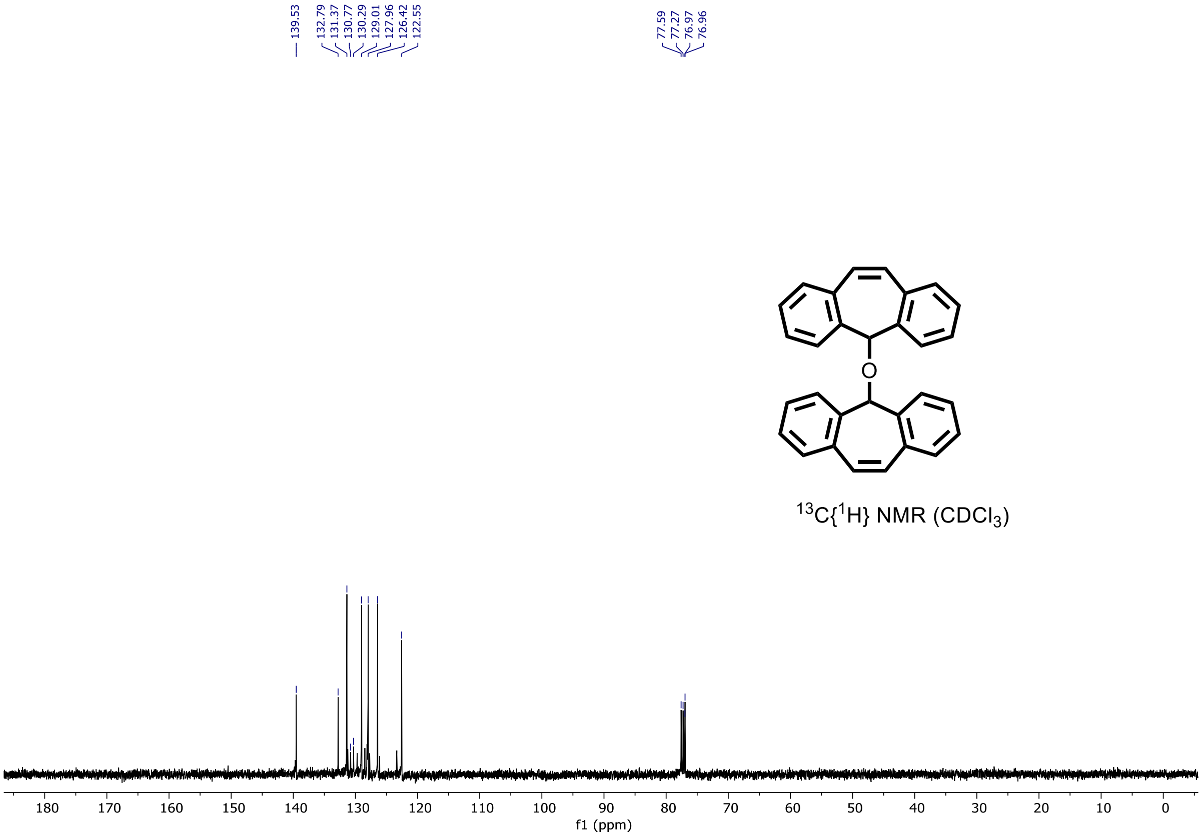
139.53
132.79
131.37
130.77
130.29
129.01
127.96
126.42
122.55
77.59
77.27
76.97
76.96
O
$^{13}C\{^1H\}$ NMR ($CDCl_3$)
f1 (ppm)

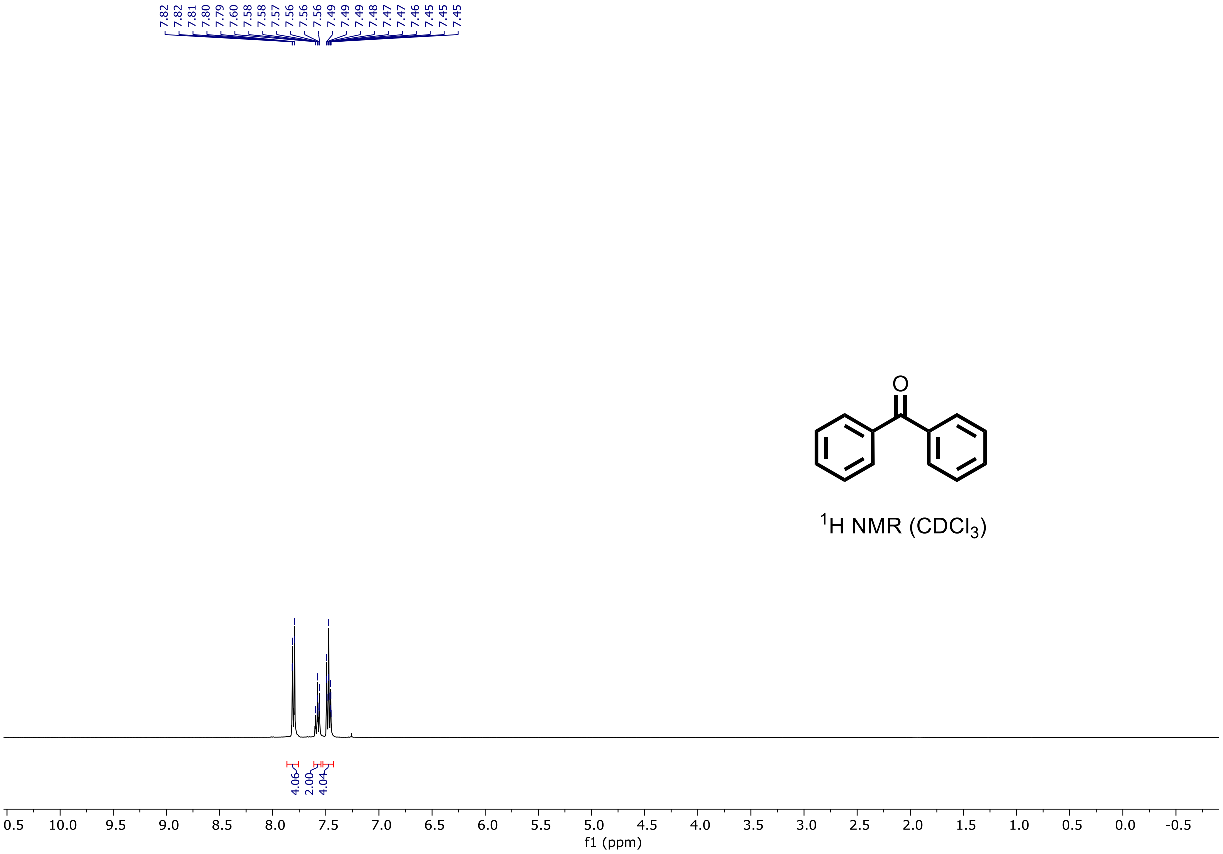
7.82
7.82
7.81
7.80
7.79
7.60
7.58
7.58
7.57
7.56
7.56
7.56
7.49
7.49
7.49
7.48
7.47
7.46
7.45
7.45
O
1H NMR (CDCl3)
4.06
2.00
4.04
0.5 10.0 9.5 9.0 8.5 8.0 7.5 7.0 6.5 6.0 5.5 5.0 4.5 4.0 3.5 3.0 2.5 2.0 1.5 1.0 0.5 0.0 -0.5
f1 (ppm)

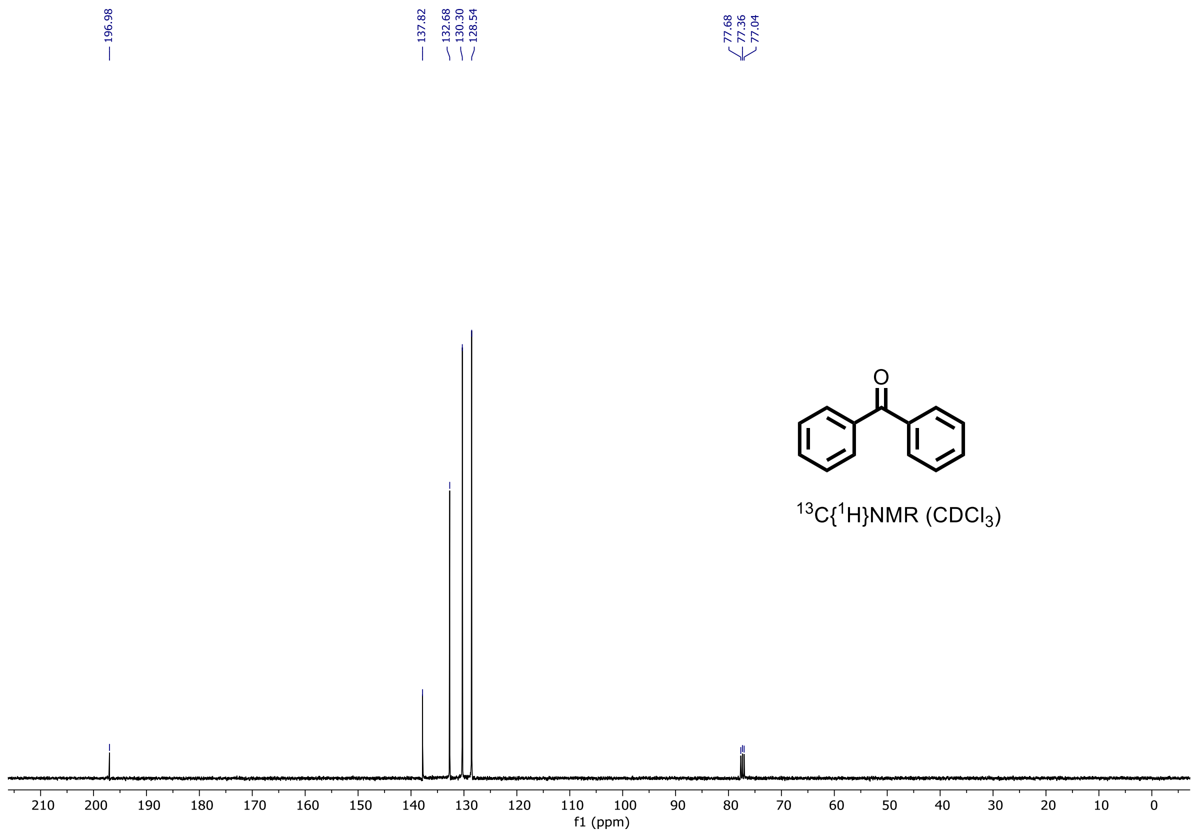
196.98
137.82
132.68
130.30
128.54
77.68
77.36
77.04
O
13C{1H}NMR (CDCl3)
210 200 190 180 170 160 150 140 130 120 110 100 90 80 70 60 50 40 30 20 10 0
f1 (ppm)

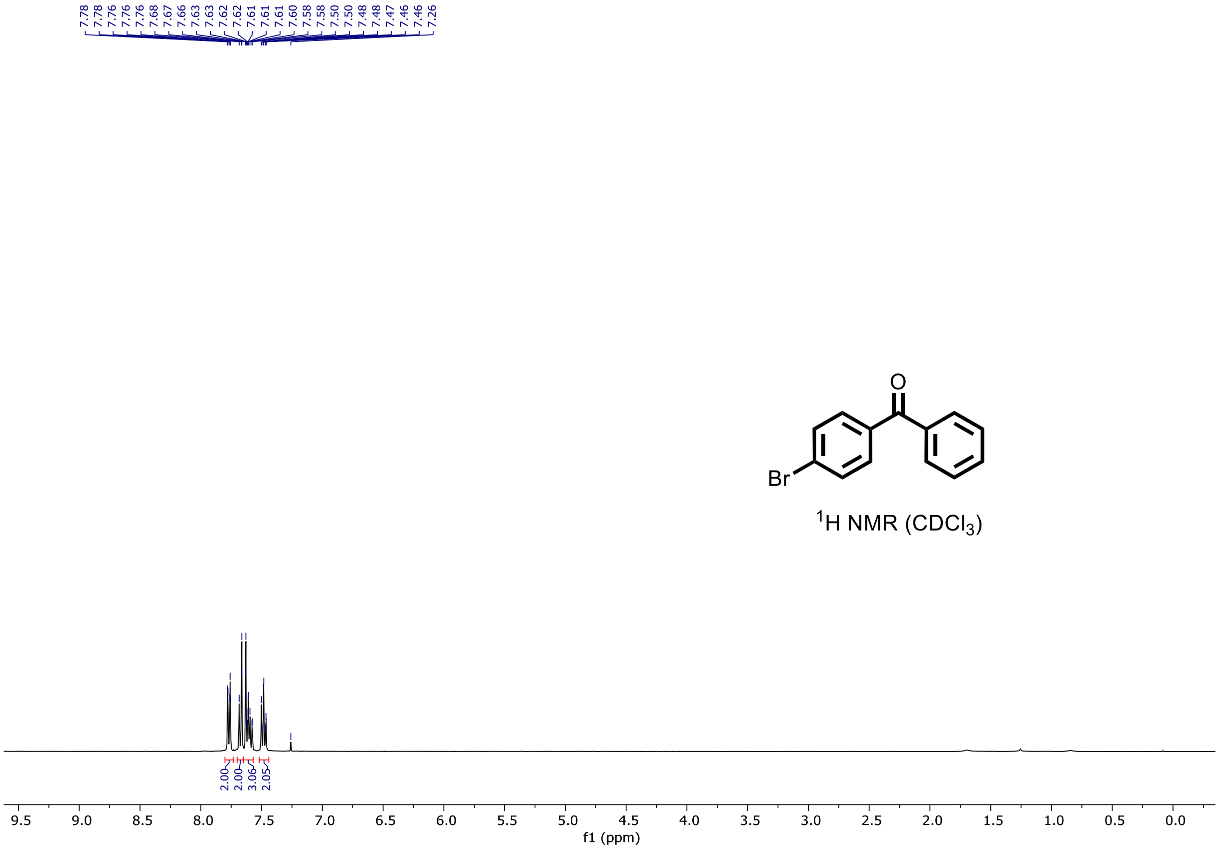
7.78 7.78 7.76 7.76 7.76 7.68 7.67 7.66 7.63 7.63 7.62 7.62 7.61 7.61 7.61 7.60 7.58 7.58 7.50 7.50 7.48 7.48 7.47 7.46 7.46 7.26
O
Br
^{1}H NMR ($CDCl_3$)
2.00
2.00
3.06
2.05
f1 (ppm)

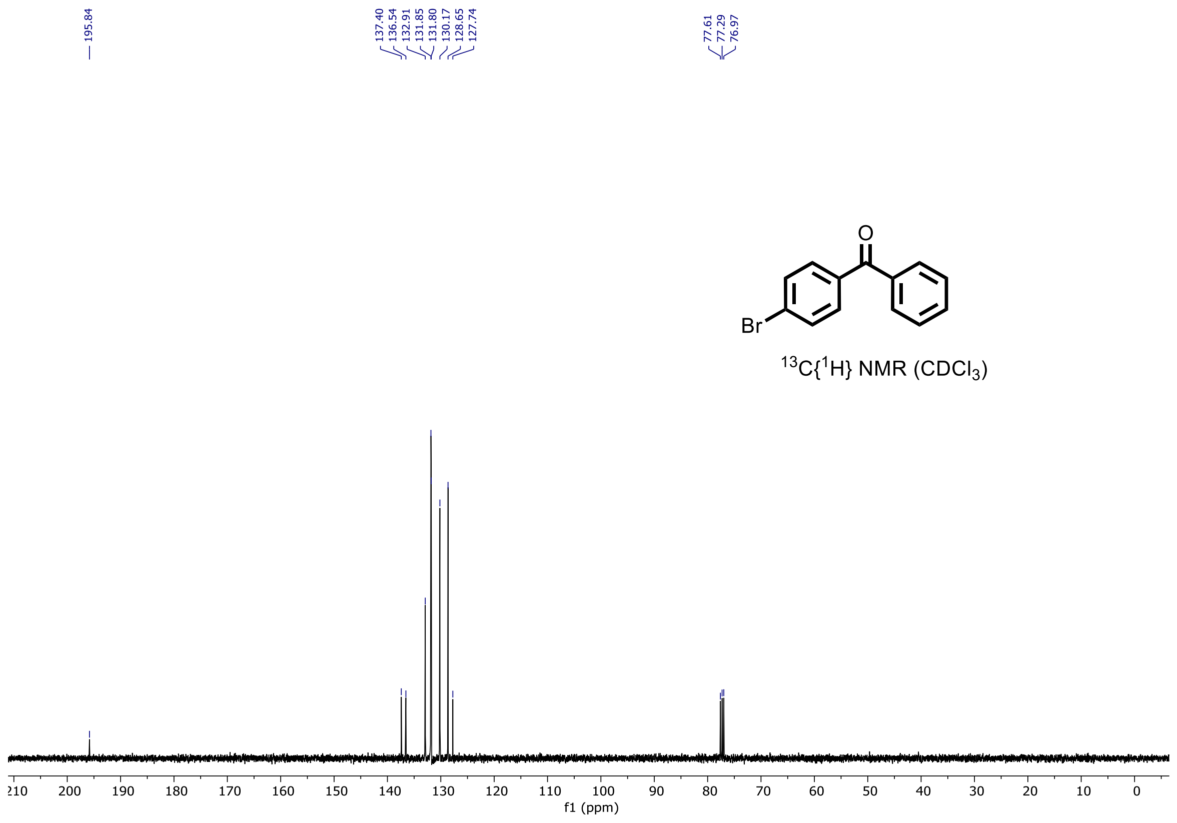
195.84
137.40 136.54 132.91 131.85 131.80 130.17 128.65 127.74
77.61 77.29 76.97
O
Br
^{13}C{^{1}H} NMR ($CDCl_3$)
f1 (ppm)

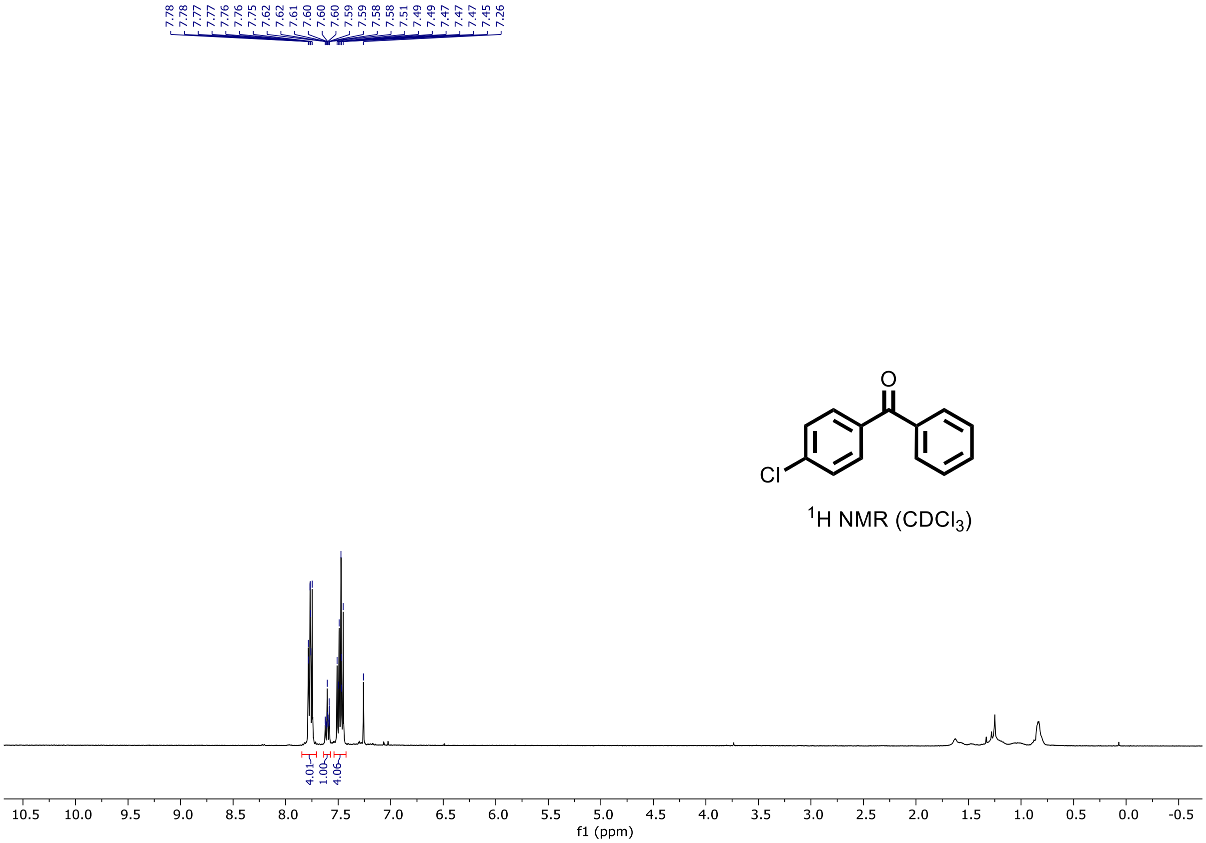
7.78 7.78 7.77 7.77 7.76 7.76 7.75 7.62 7.62 7.61 7.60 7.60 7.60 7.59 7.59 7.58 7.58 7.51 7.49 7.49 7.47 7.47 7.47 7.45 7.26
Cl
O
^{1}H NMR ($CDCl_3$)
4.01
1.00
4.06
10.5 10.0 9.5 9.0 8.5 8.0 7.5 7.0 6.5 6.0 5.5 5.0 4.5 4.0 3.5 3.0 2.5 2.0 1.5 1.0 0.5 0.0 -0.5
f1 (ppm)

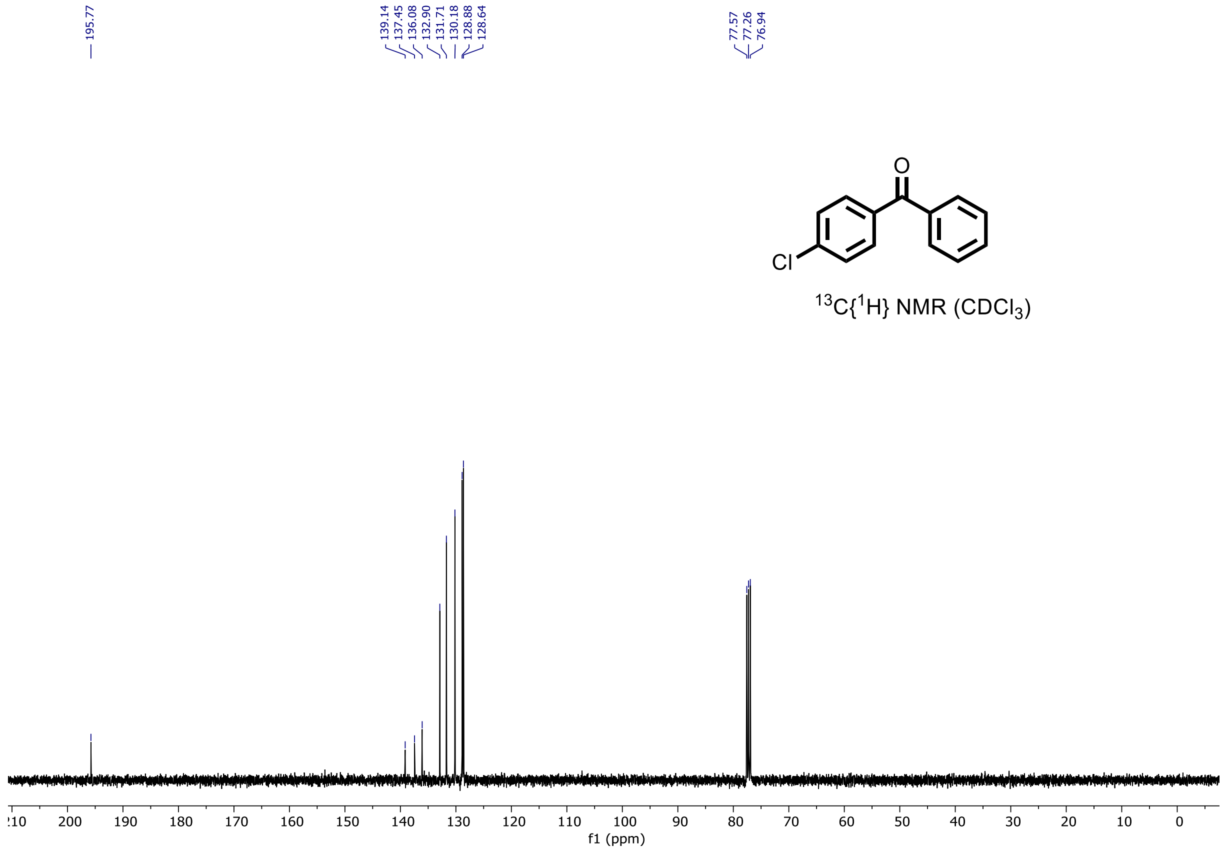
195.77
139.14 137.45 136.08 132.90 131.71 130.18 128.88 128.64
77.57 77.26 76.94
Cl
O
$^{13}C\{^1H\}$ NMR ($CDCl_3$)
210 200 190 180 170 160 150 140 130 120 110 100 90 80 70 60 50 40 30 20 10 0
f1 (ppm)

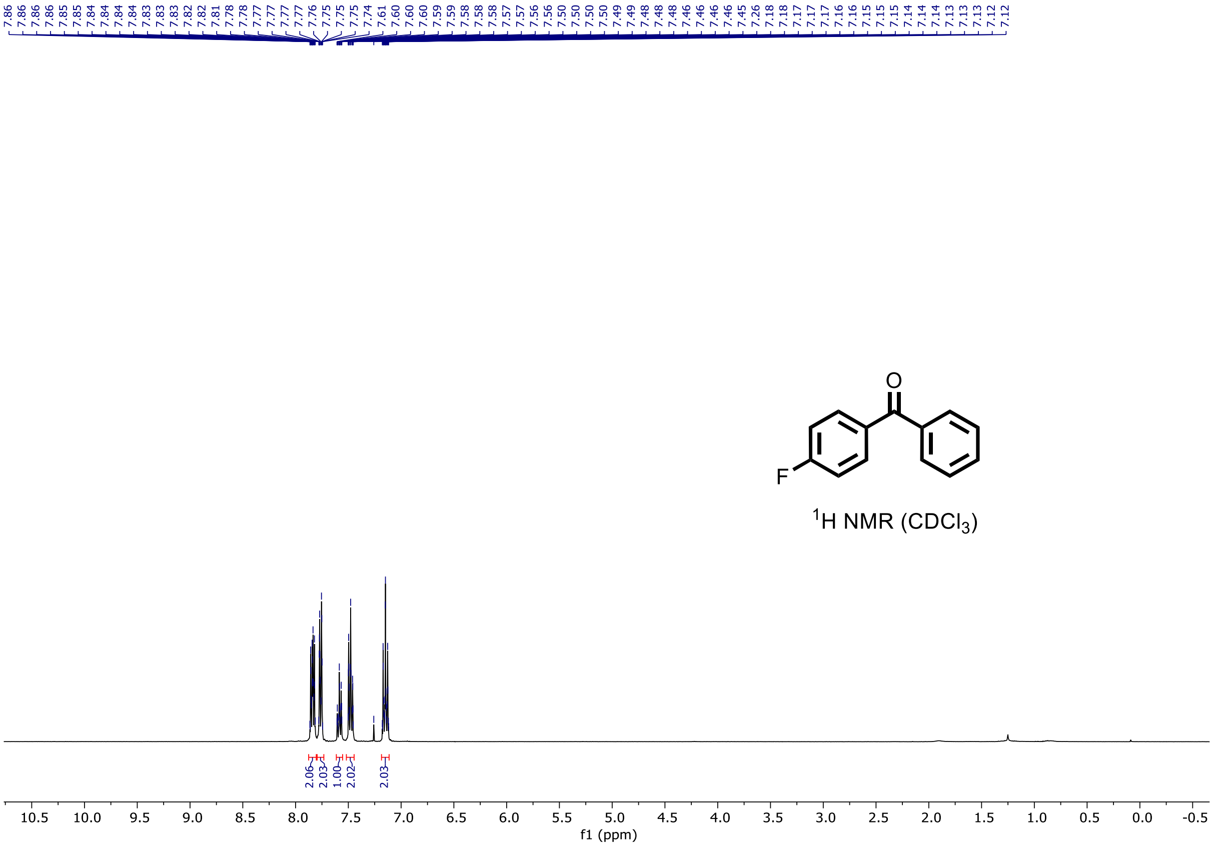

7.86 7.86 7.86 7.85 7.84 7.84 7.84 7.83 7.83 7.82 7.81 7.78 7.77 7.77 7.77 7.76 7.75 7.75 7.75 7.74 7.61 7.60 7.60 7.59 7.59 7.58 7.58 7.58 7.57 7.57 7.56 7.56 7.50 7.50 7.50 7.50 7.49 7.49 7.48 7.48 7.46 7.46 7.46 7.45 7.26 7.18 7.18 7.17 7.17 7.17 7.16 7.16 7.15 7.15 7.15 7.14 7.14 7.13 7.13 7.13 7.12 7.12
O
F
1H NMR (CDCl3)
2.06
2.03
1.00
2.02
2.03
10.5 10.0 9.5 9.0 8.5 8.0 7.5 7.0 6.5 6.0 5.5 5.0 4.5 4.0 3.5 3.0 2.5 2.0 1.5 1.0 0.5 0.0 -0.5
f1 (ppm)

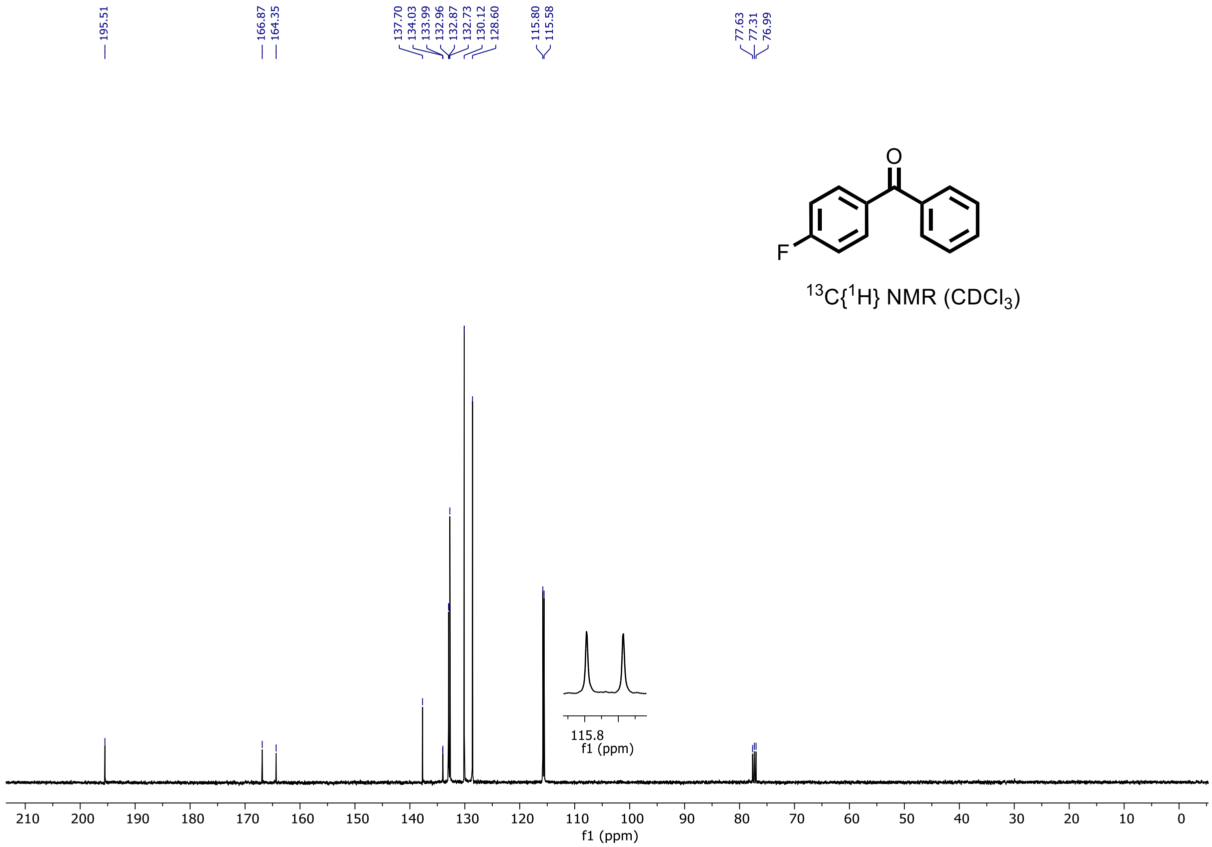

195.51
166.87
164.35
137.70
134.03
133.99
132.96
132.87
132.73
130.12
128.60
115.80
115.58
77.63
77.31
76.99
O
F
13C{1H} NMR (CDCl3)
115.8
f1 (ppm)
210 200 190 180 170 160 150 140 130 120 110 100 90 80 70 60 50 40 30 20 10 0
f1 (ppm)

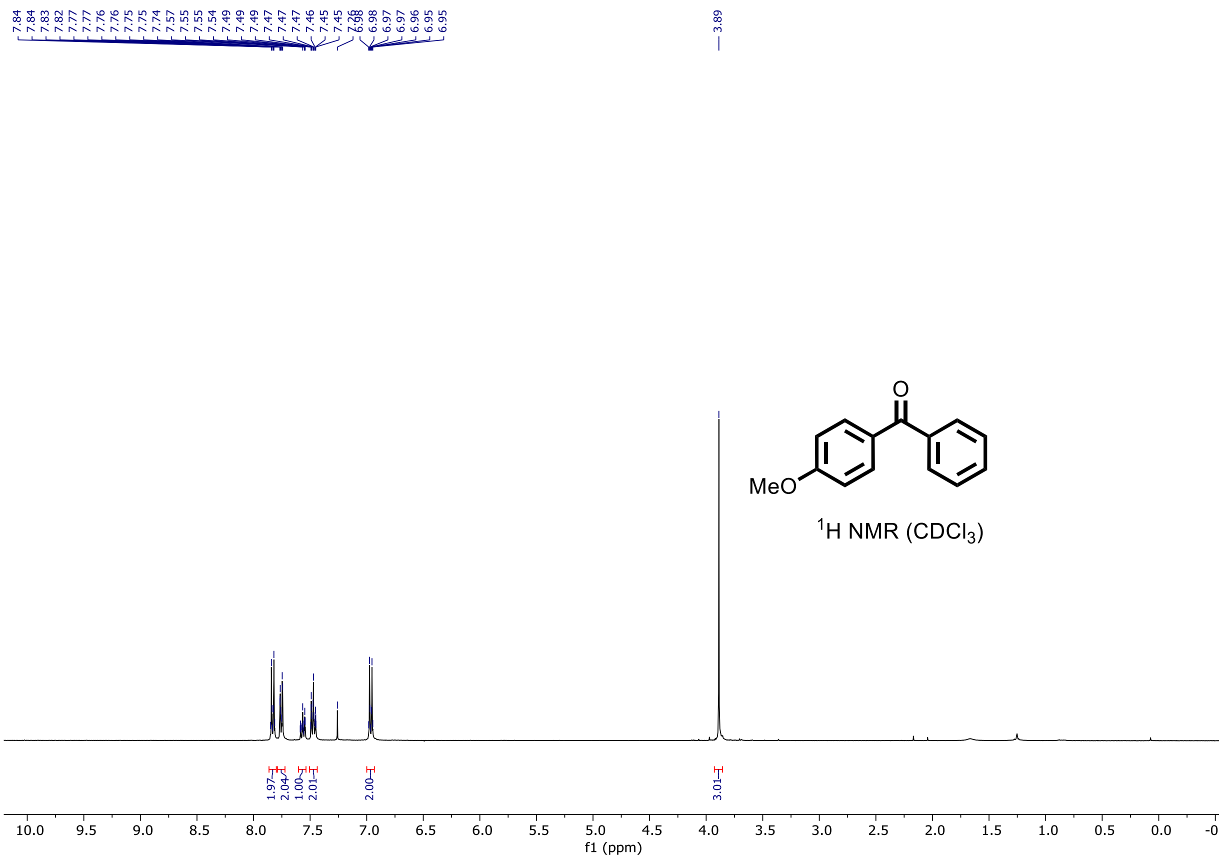

MeO
O
^{1}H NMR ($CDCl_3$)
f1 (ppm)

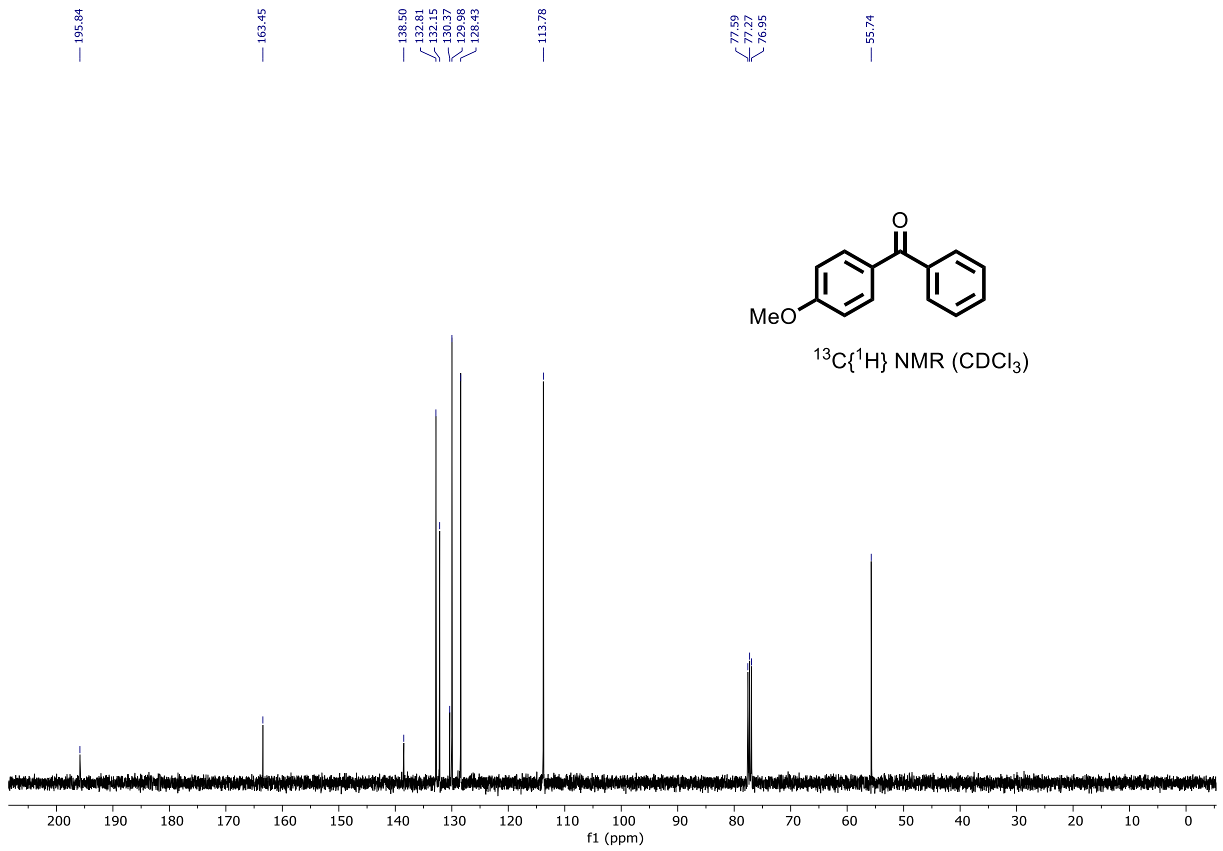

MeO
O
$^{13}C\{^1H\}$ NMR ($CDCl_3$)
f1 (ppm)

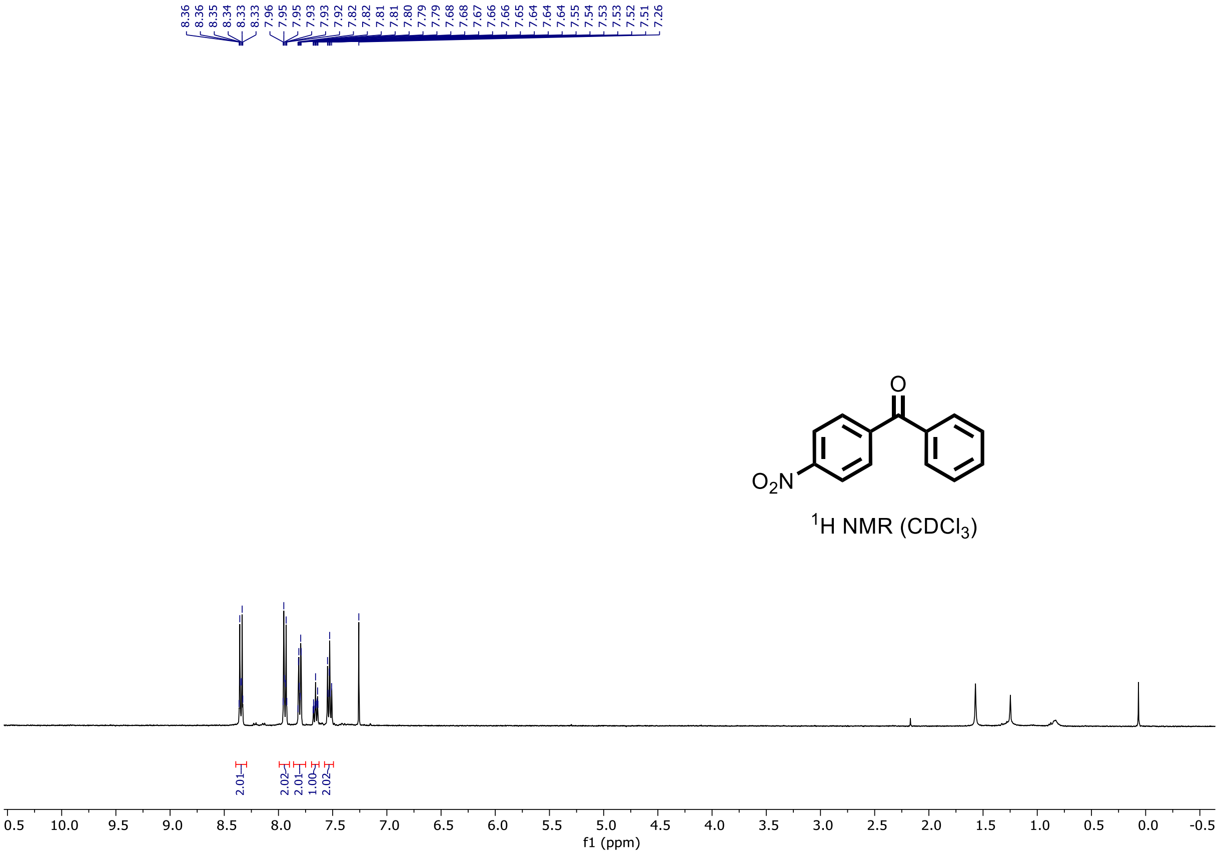
8.36 8.36 8.35 8.34 8.33 8.33 7.96 7.95 7.95 7.93 7.93 7.92 7.82 7.82 7.81 7.80 7.79 7.79 7.68 7.68 7.67 7.66 7.66 7.65 7.64 7.64 7.64 7.55 7.54 7.53 7.53 7.52 7.51 7.26
O
O_2N
^1H NMR ($CDCl_3$)
2.01
2.02
2.01
1.00
2.02
f1 (ppm)

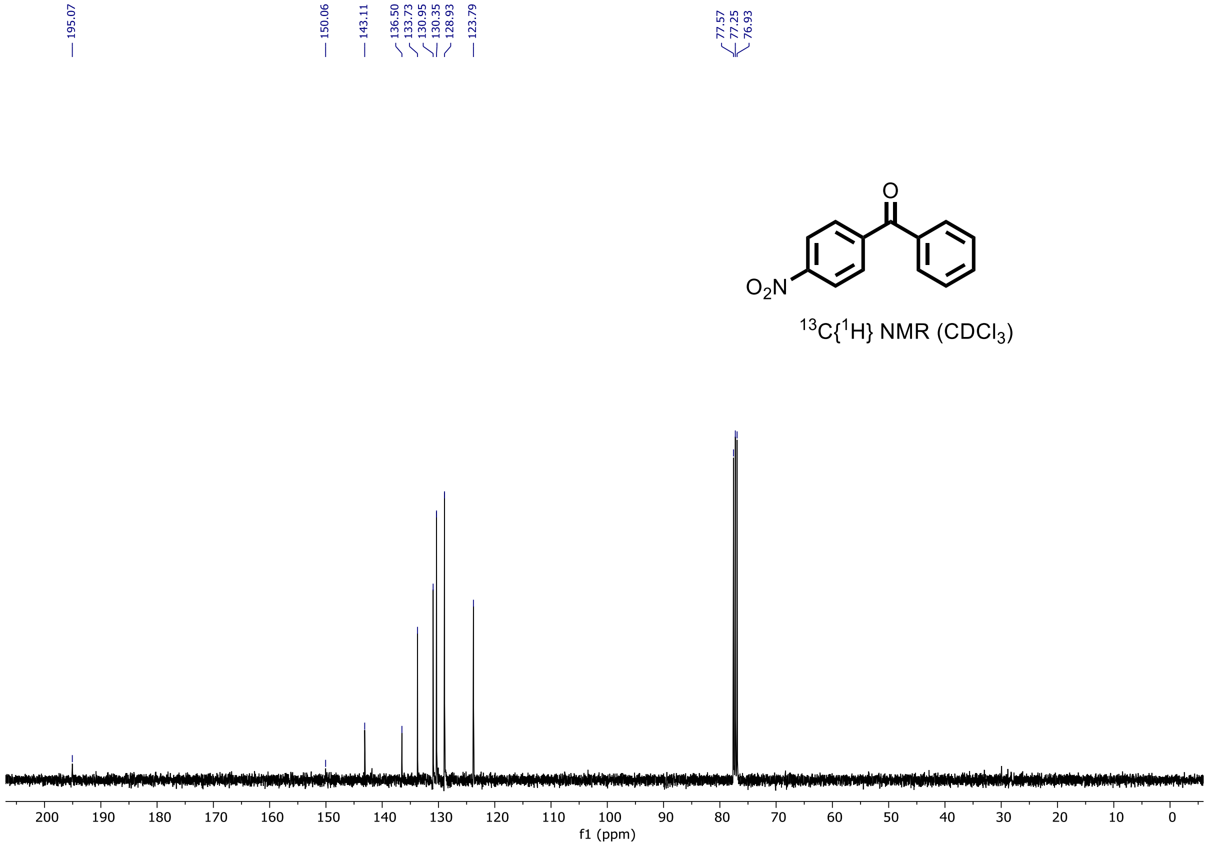
195.07
150.06
143.11
136.50 133.73 130.95 130.35 128.93
123.79
77.57 77.25 76.93
O
O_2N
$^{13}C\{^1H\}$ NMR ($CDCl_3$)
f1 (ppm)

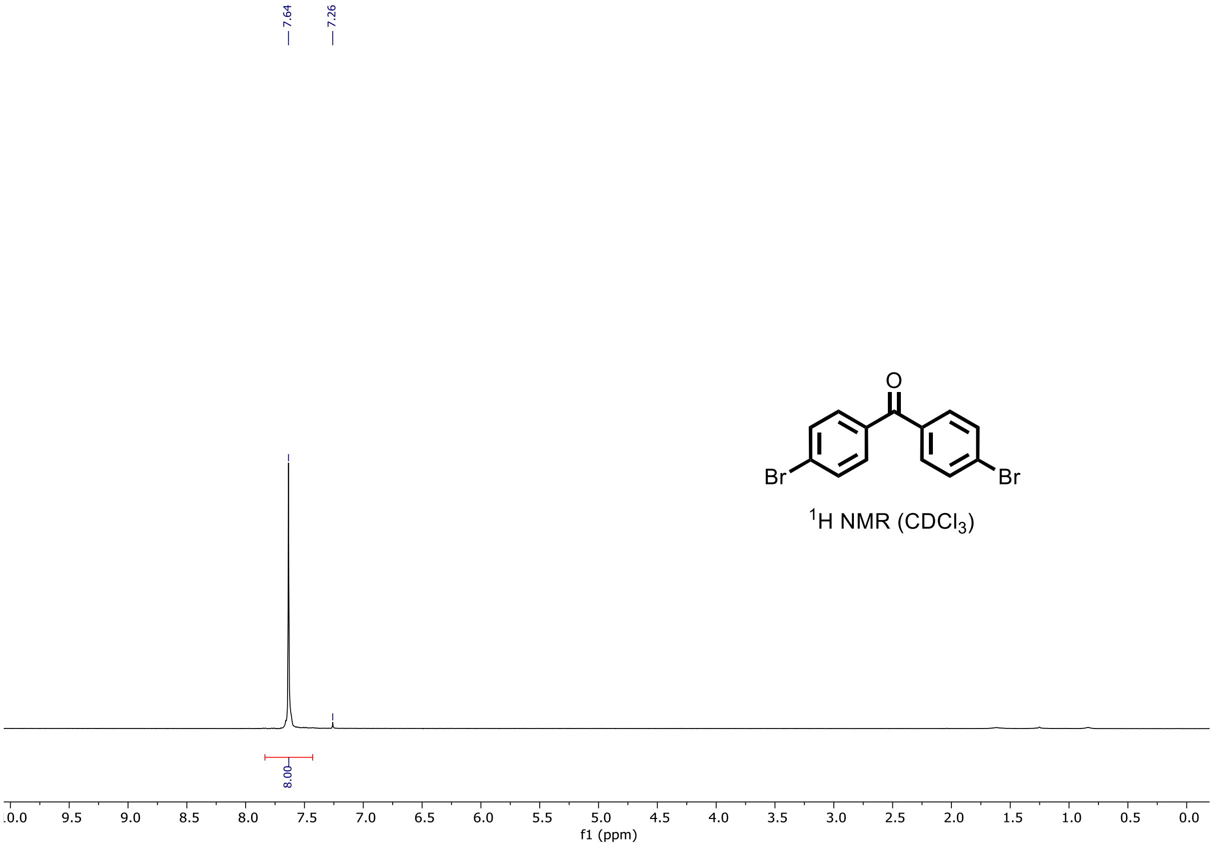
7.64
7.26
Br
O
Br
^{1}H NMR ($CDCl_3$)
8.00
f1 (ppm)

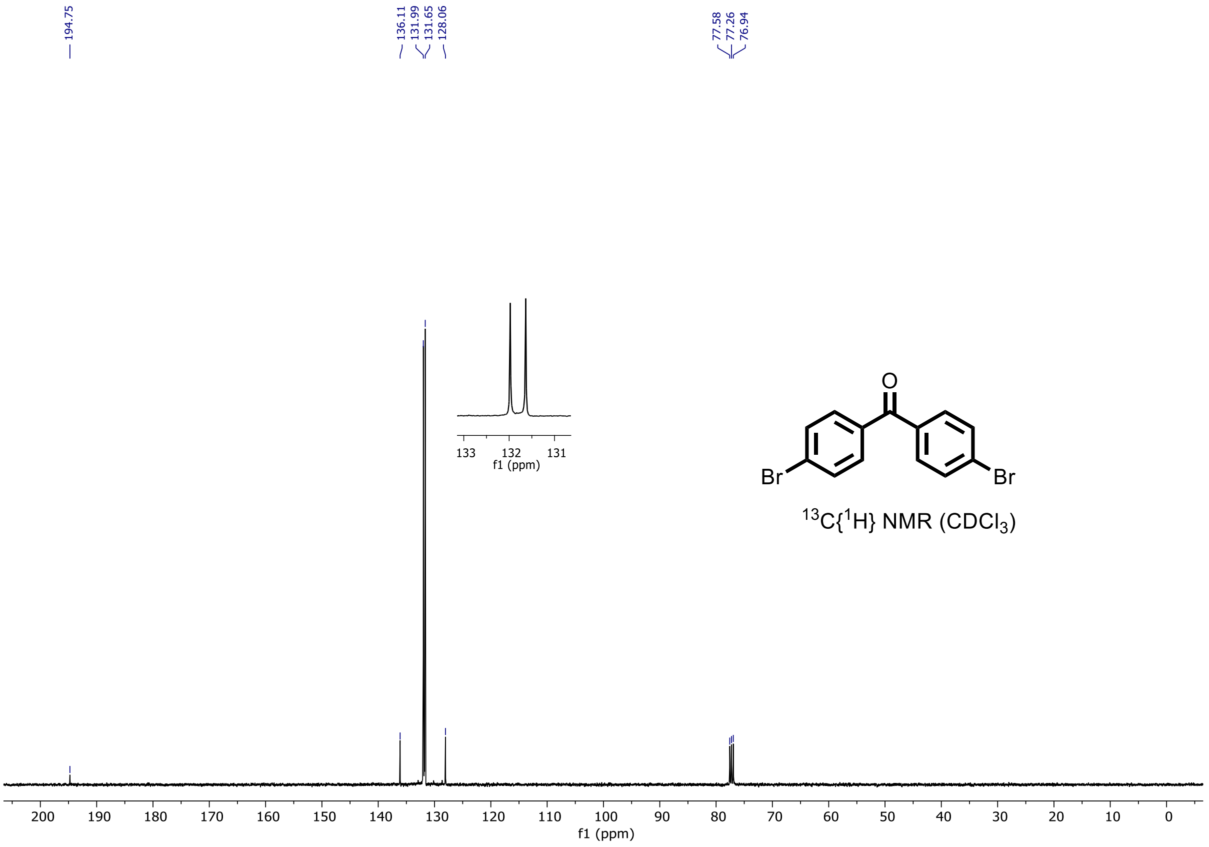
194.75
136.11
131.99
131.65
128.06
77.58
77.26
76.94
133
132
131
f1 (ppm)
Br
O
Br
^{13}C{^{1}H} NMR ($CDCl_3$)
f1 (ppm)

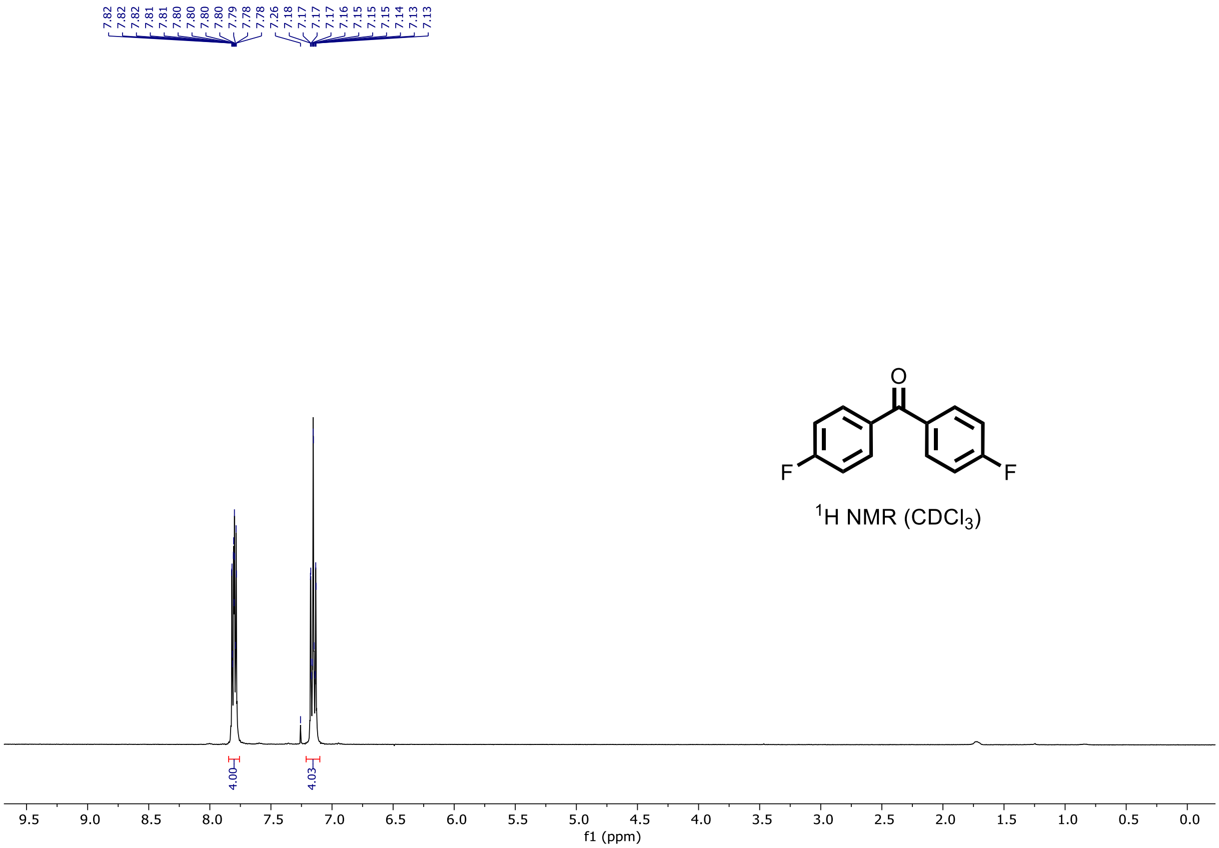
7.82 7.82 7.82 7.81 7.81 7.80 7.80 7.80 7.80 7.79 7.78 7.78 7.26 7.18 7.17 7.17 7.17 7.16 7.15 7.15 7.15 7.14 7.13 7.13
O
F
F
1H NMR (CDCl3)
4.00
4.03
9.5 9.0 8.5 8.0 7.5 7.0 6.5 6.0 5.5 5.0 4.5 4.0 3.5 3.0 2.5 2.0 1.5 1.0 0.5 0.0
f1 (ppm)

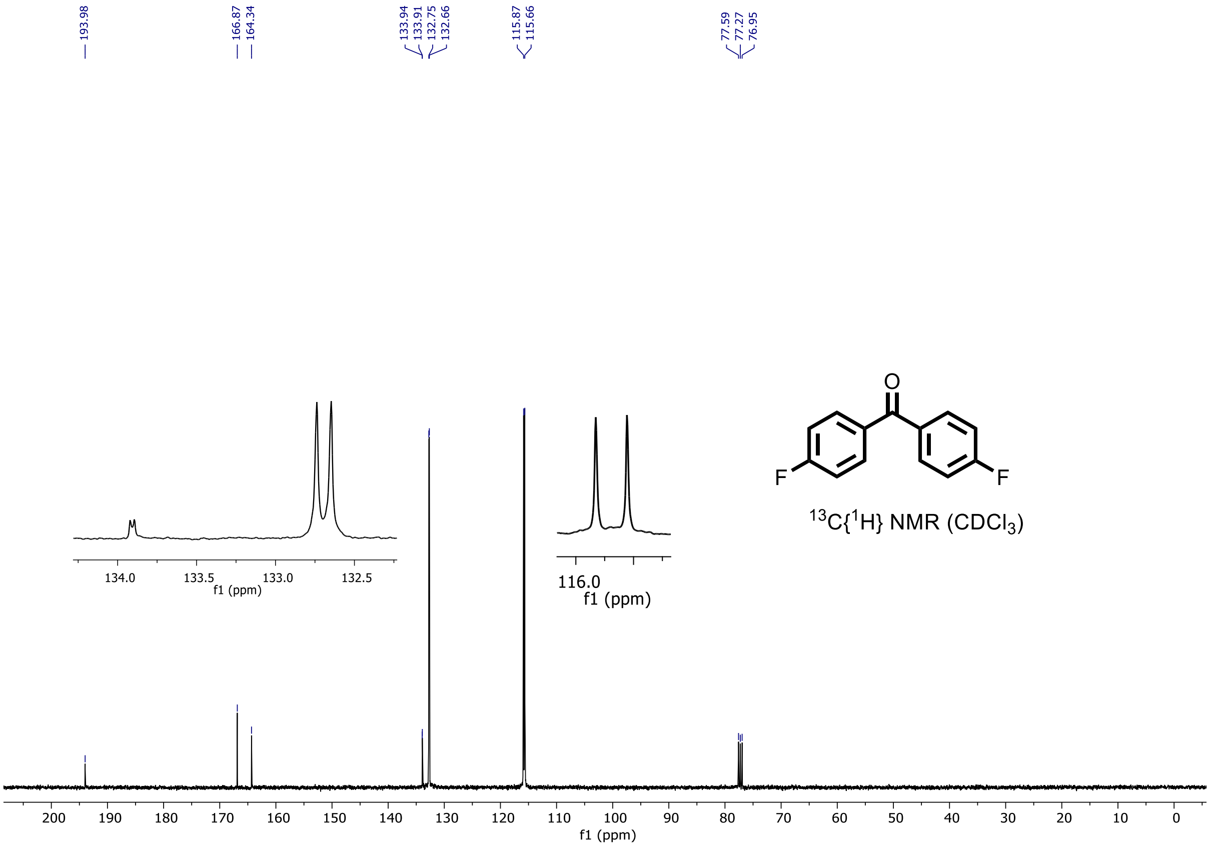
193.98
166.87 164.34
133.94 133.91 132.75 132.66
115.87 115.66
77.59 77.27 76.95
O
F
F
13C{1H} NMR (CDCl3)
134.0 133.5 133.0 132.5
f1 (ppm)
116.0
f1 (ppm)
200 190 180 170 160 150 140 130 120 110 100 90 80 70 60 50 40 30 20 10 0
f1 (ppm)

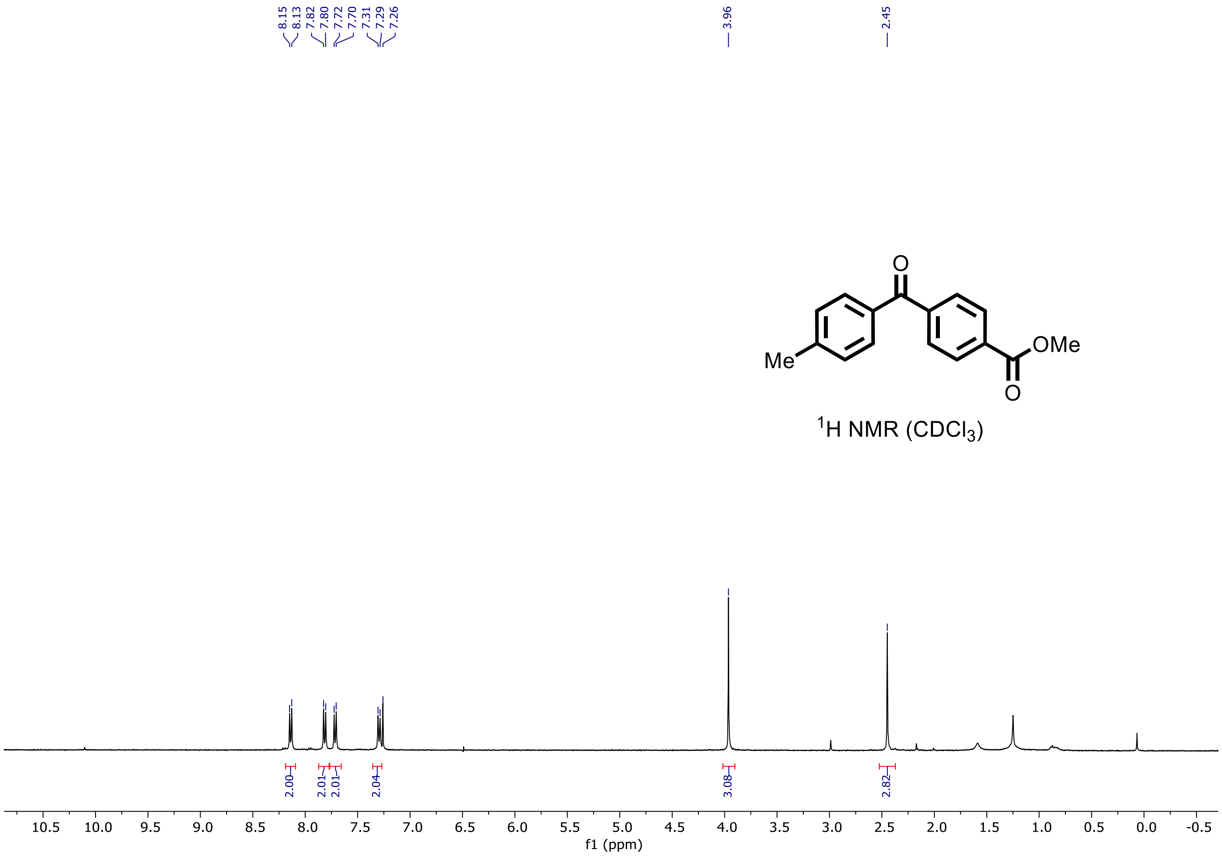
8.15
8.13
7.82
7.80
7.72
7.70
7.31
7.29
7.26
3.96
2.45
Me
OMe
O
O
^{1}H NMR ($CDCl_3$)
2.00
2.01
2.01
2.04
3.08
2.82
f1 (ppm)

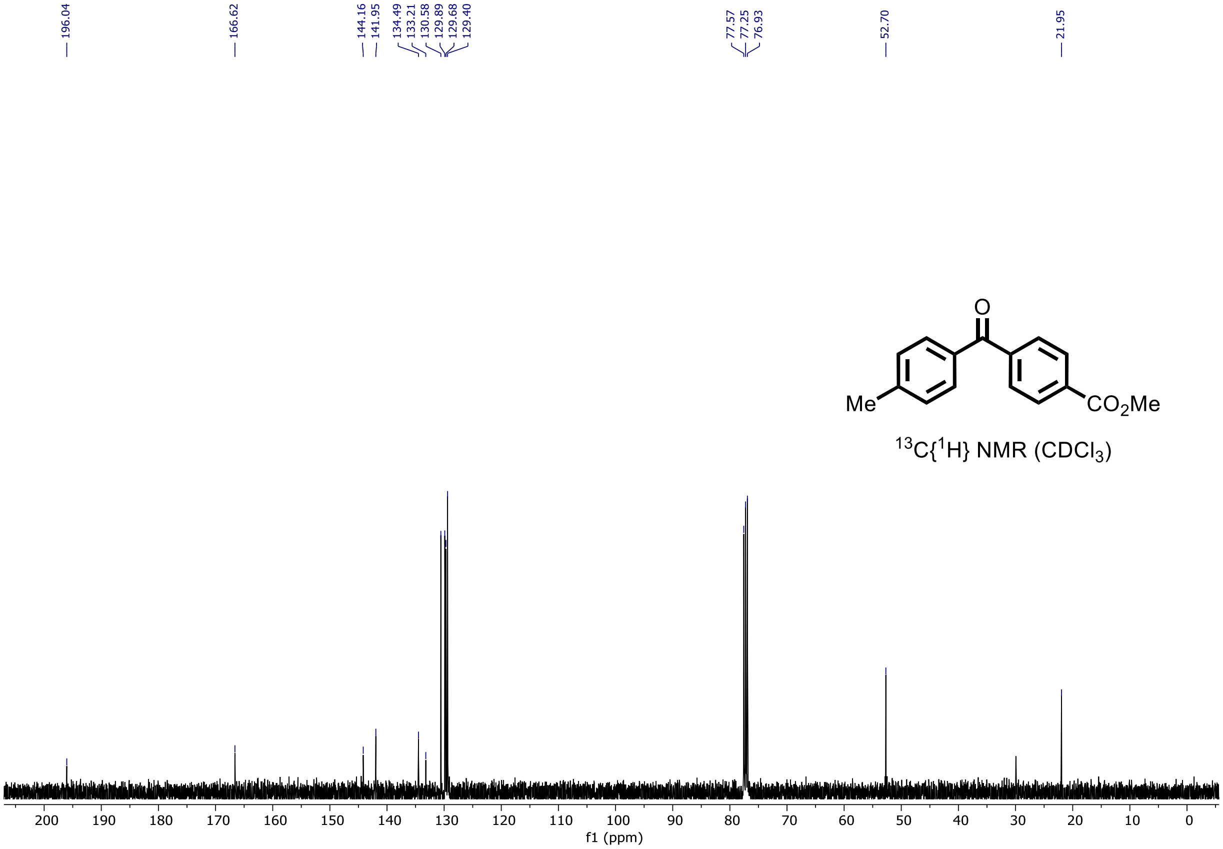
196.04
166.62
144.16
141.95
134.49
133.21
130.58
129.89
129.68
129.40
77.57
77.25
76.93
52.70
21.95
Me
O
CO_2Me
$^{13}C\{^{1}H\}$ NMR ($CDCl_3$)
f1 (ppm)

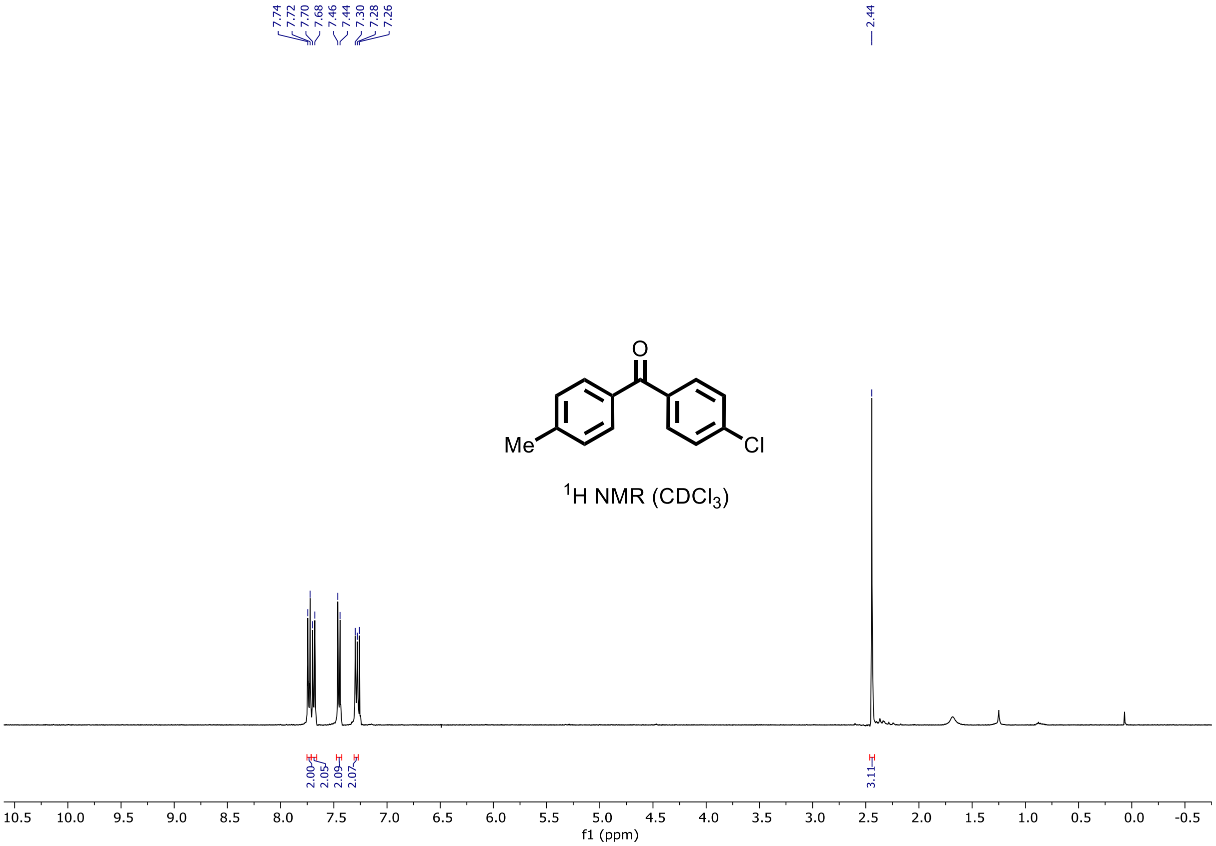
7.74
7.72
7.70
7.68
7.46
7.44
7.30
7.28
7.26
2.44
O
Me
Cl
^{1}H NMR (CDCl$_3$)
2.00
2.05
2.09
2.07
3.11
10.5 10.0 9.5 9.0 8.5 8.0 7.5 7.0 6.5 6.0 5.5 5.0 4.5 4.0 3.5 3.0 2.5 2.0 1.5 1.0 0.5 0.0 -0.5
f1 (ppm)

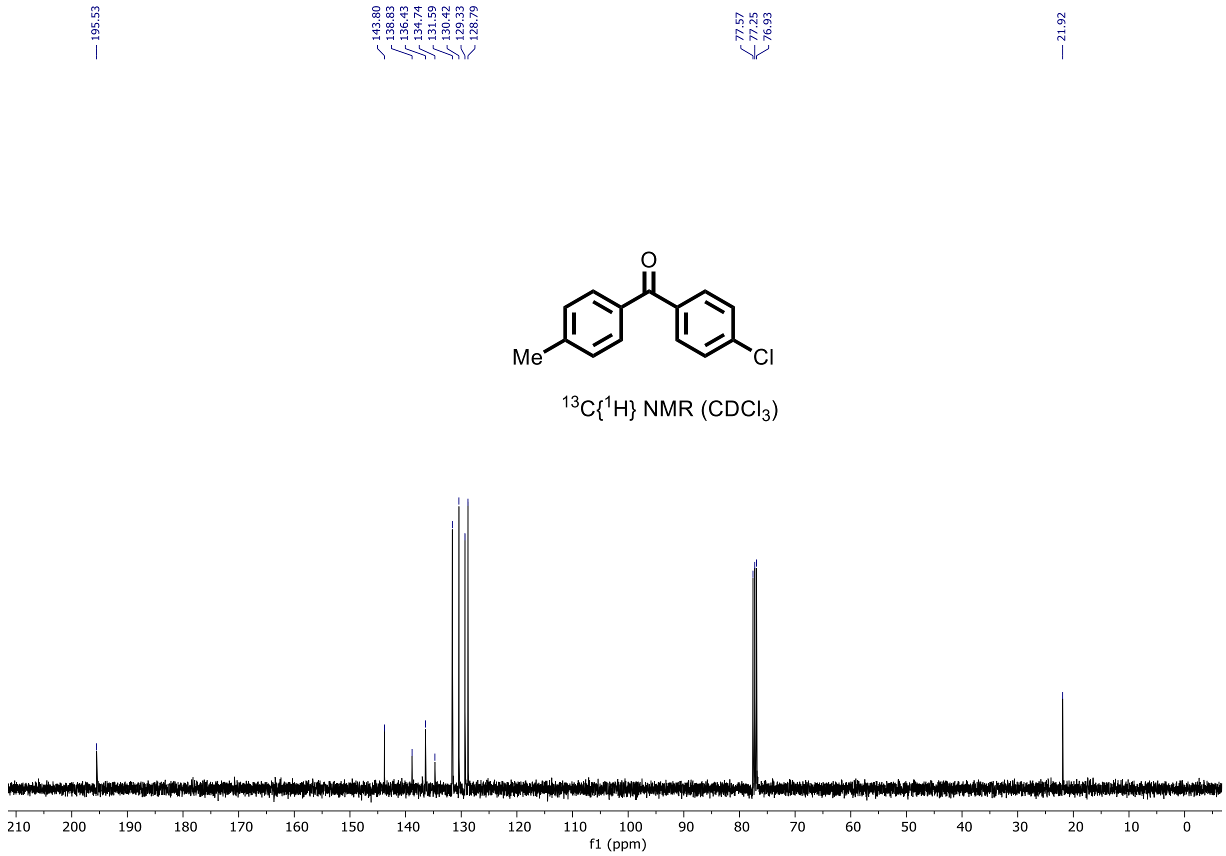
195.53
143.80
138.83
136.43
134.74
131.59
130.42
129.33
128.79
77.57
77.25
76.93
21.92
O
Me
Cl
^{13}C{^{1}H} NMR (CDCl$_3$)
210 200 190 180 170 160 150 140 130 120 110 100 90 80 70 60 50 40 30 20 10 0
f1 (ppm)

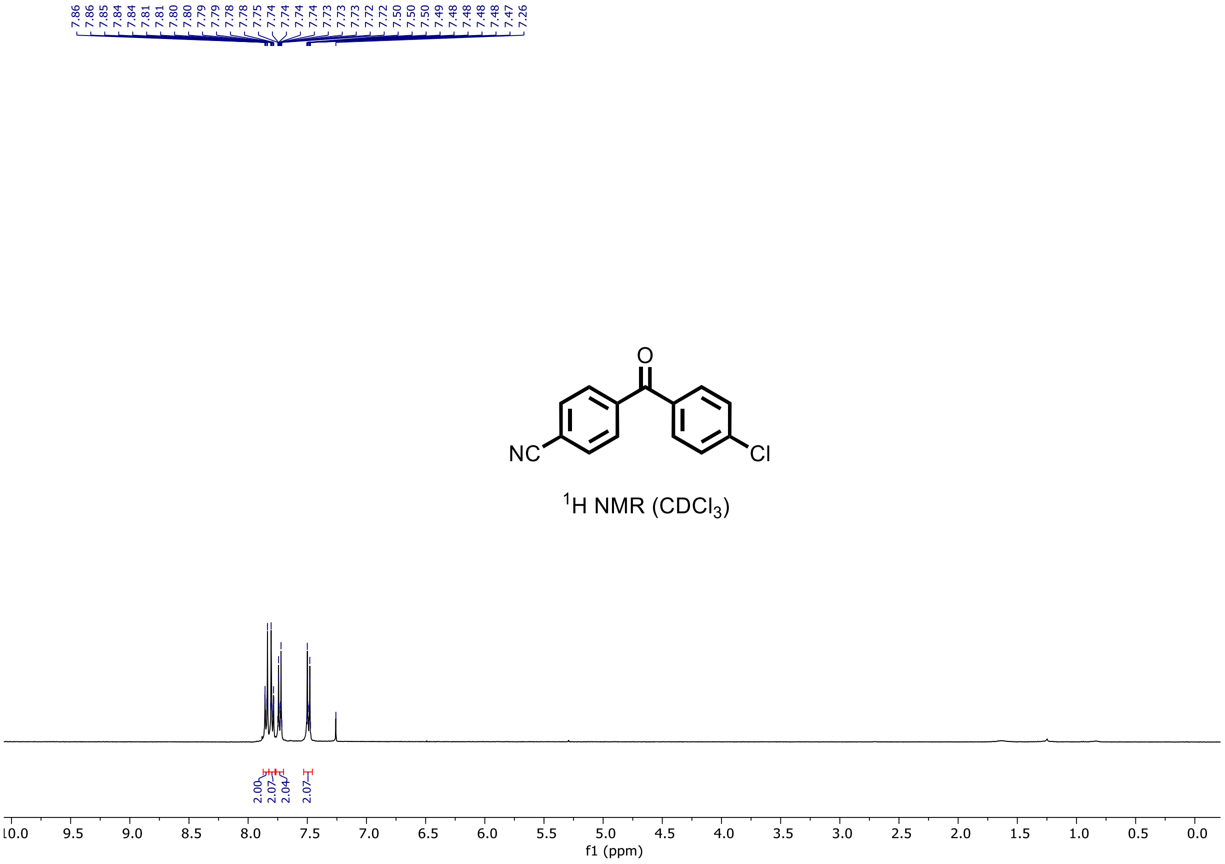
NC
Cl
O
^{1}H NMR (CDCl$_3$)
f1 (ppm)

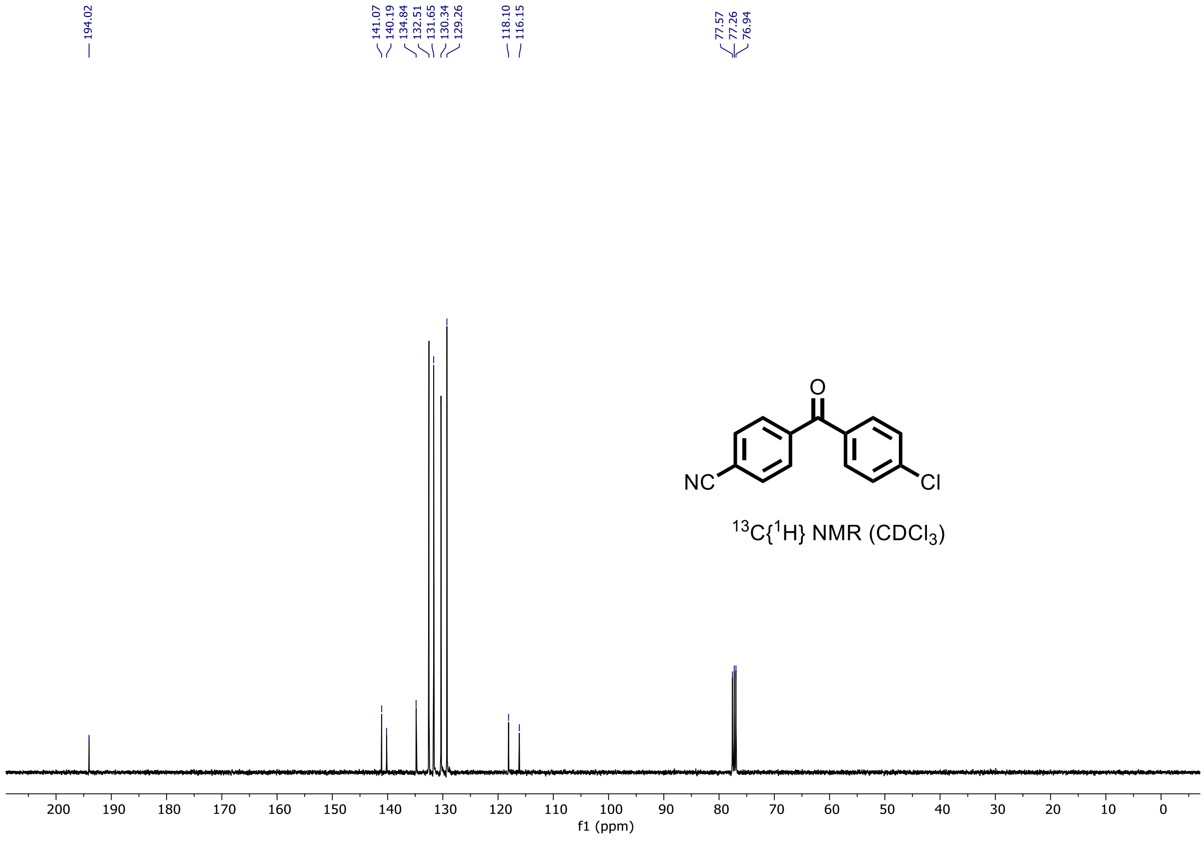
NC
Cl
O
^{13}C{^{1}H} NMR (CDCl$_3$)
f1 (ppm)

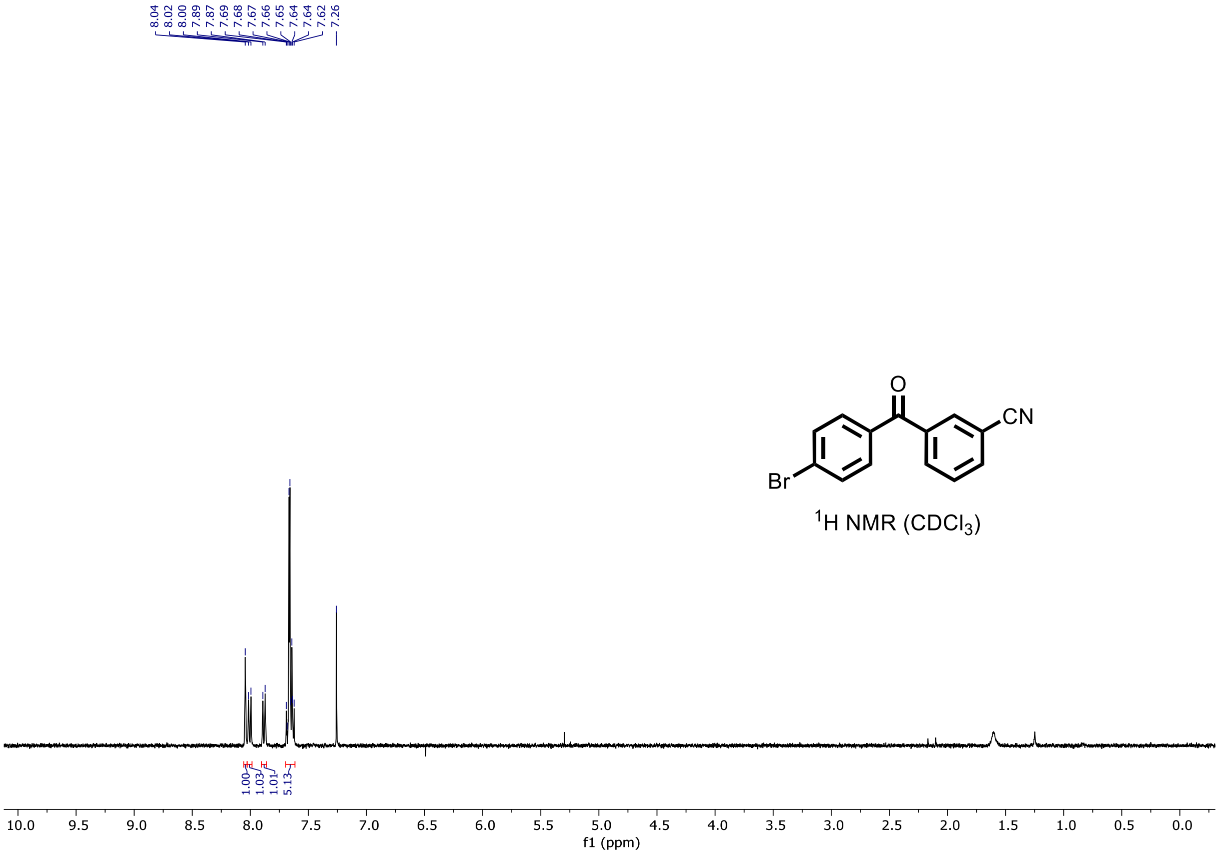
8.04
8.02
8.00
7.89
7.87
7.69
7.68
7.67
7.66
7.65
7.64
7.64
7.62
7.26
CN
O
Br
^{1}H NMR (CDCl$_3$)
1.00
1.03
1.01
5.13
10.0 9.5 9.0 8.5 8.0 7.5 7.0 6.5 6.0 5.5 5.0 4.5 4.0 3.5 3.0 2.5 2.0 1.5 1.0 0.5 0.0
f1 (ppm)

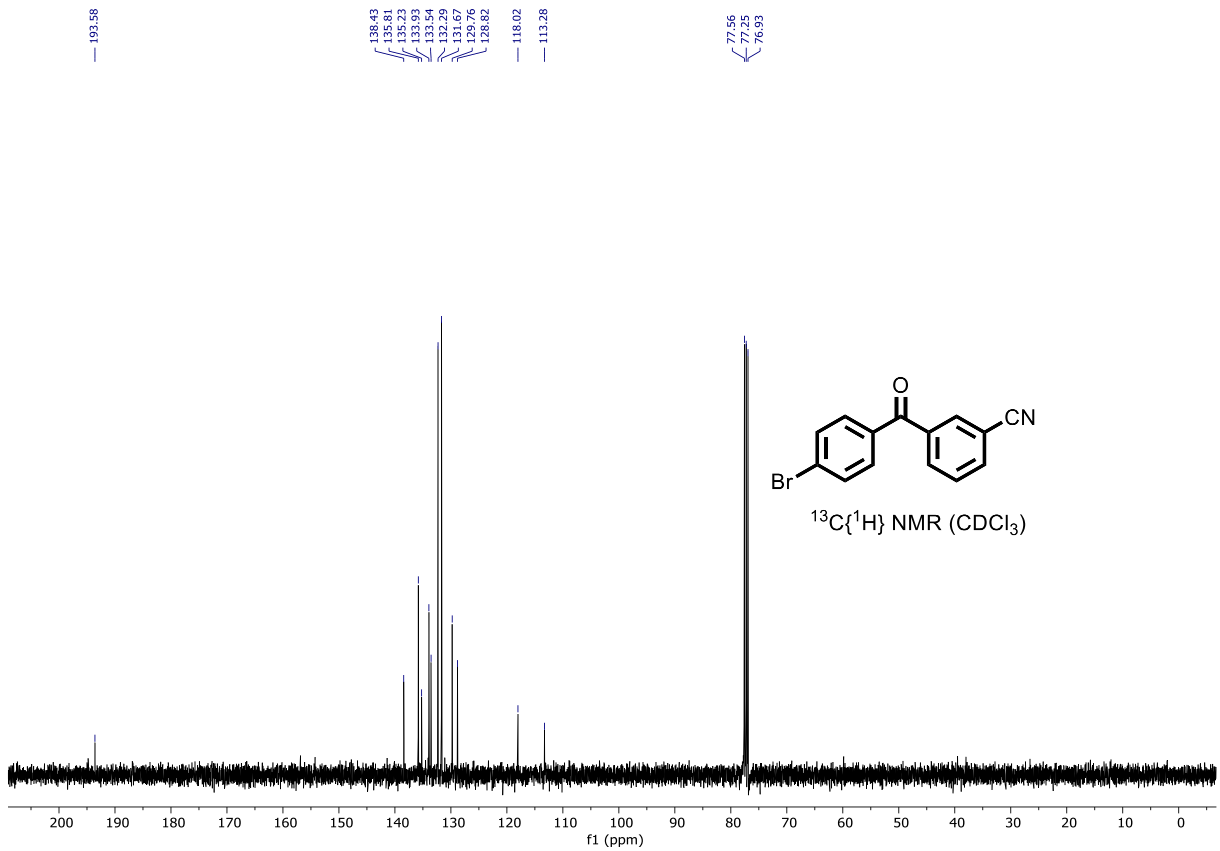
193.58
138.43
135.81
135.23
133.93
133.54
132.29
131.67
129.76
128.82
118.02
113.28
77.56
77.25
76.93
O
CN
Br
^{13}C{^{1}H} NMR (CDCl$_3$)
200 190 180 170 160 150 140 130 120 110 100 90 80 70 60 50 40 30 20 10 0
f1 (ppm)

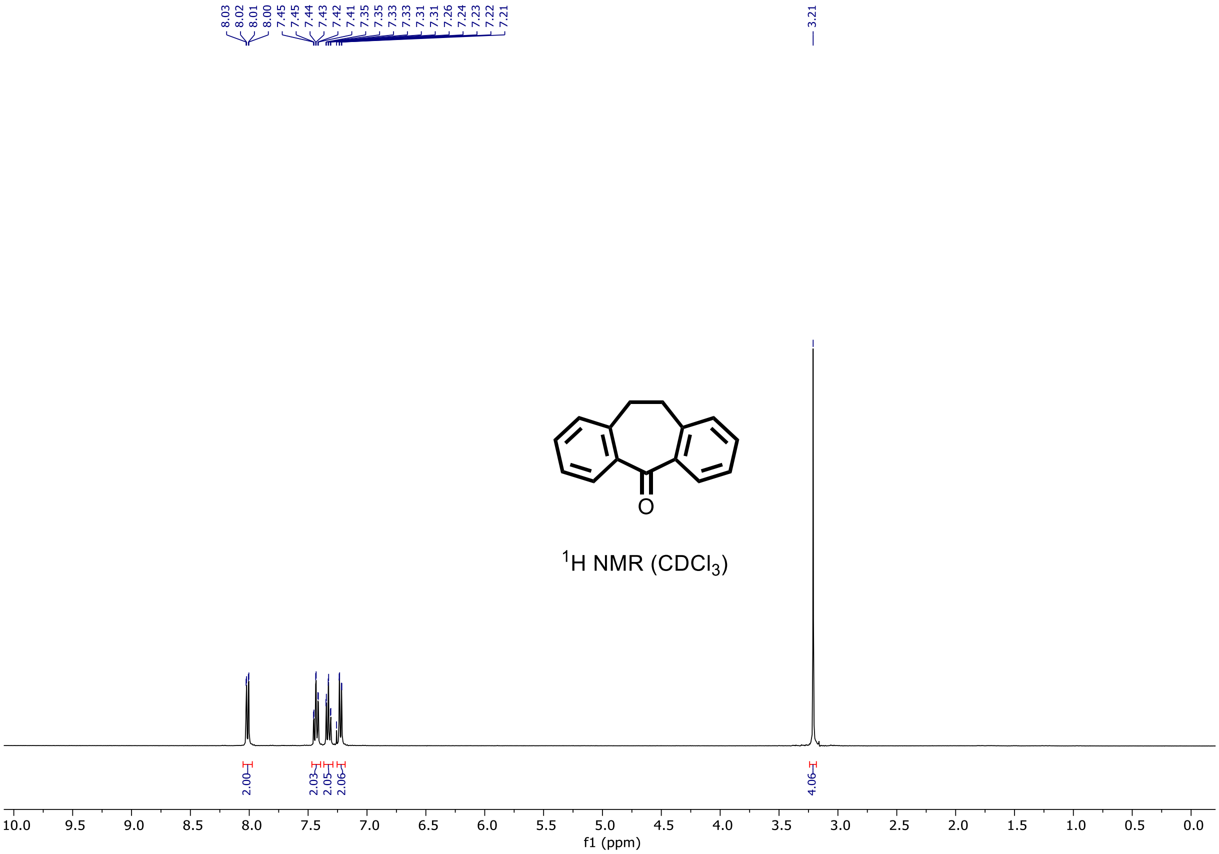

8.03
8.02
8.01
8.00
7.45
7.45
7.44
7.43
7.42
7.41
7.35
7.35
7.33
7.33
7.31
7.31
7.26
7.24
7.23
7.22
7.21
3.21
O
^{1}H NMR ($CDCl_3$)
2.00
2.03
2.05
2.06
4.06
10.0 9.5 9.0 8.5 8.0 7.5 7.0 6.5 6.0 5.5 5.0 4.5 4.0 3.5 3.0 2.5 2.0 1.5 1.0 0.5 0.0
f1 (ppm)

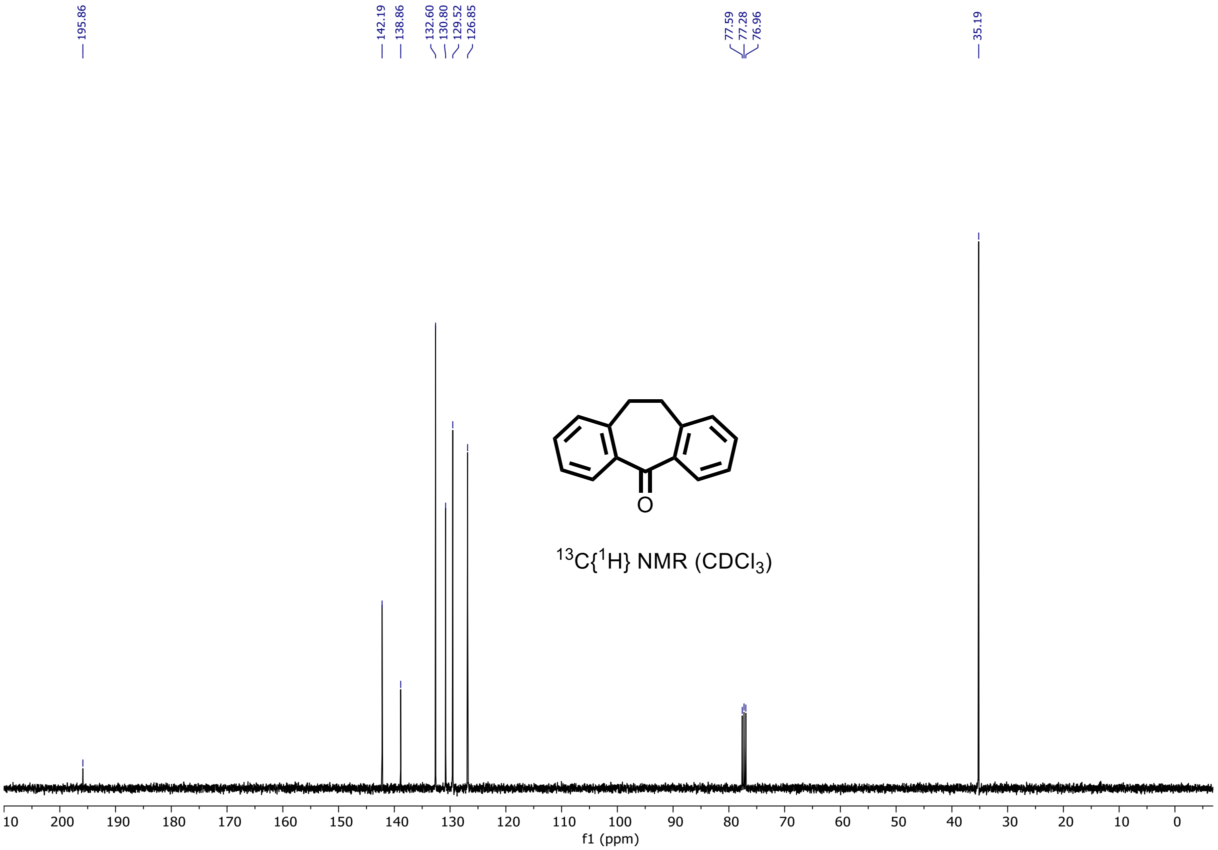

195.86
142.19
138.86
132.60
130.80
129.52
126.85
77.59
77.28
76.96
35.19
O
$^{13}C\{^{1}H\}$ NMR ($CDCl_3$)
10 200 190 180 170 160 150 140 130 120 110 100 90 80 70 60 50 40 30 20 10 0
f1 (ppm)

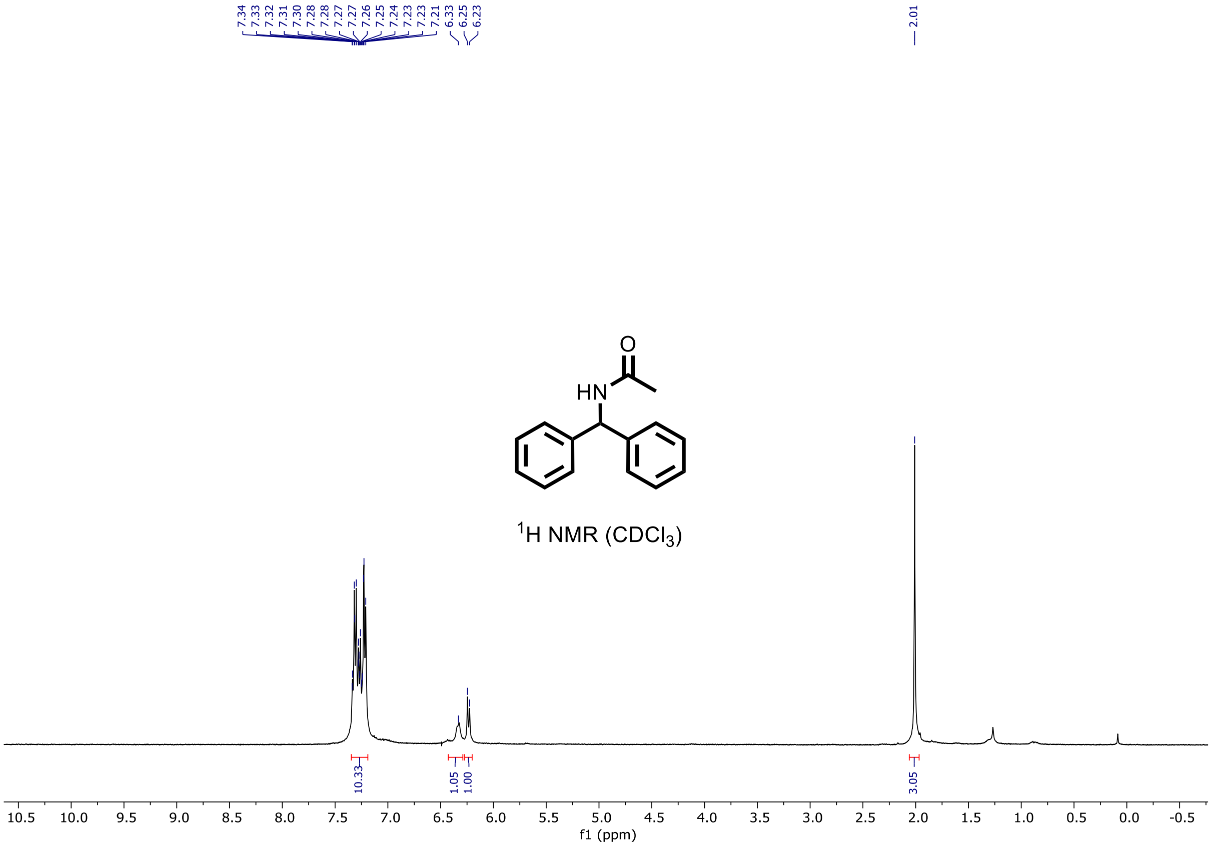
7.34
7.33
7.32
7.31
7.30
7.28
7.28
7.27
7.27
7.26
7.25
7.24
7.23
7.23
7.21
6.33
6.25
6.23
2.01
O
HN
1H NMR (CDCl3)
10.33
1.05
1.00
3.05
10.5 10.0 9.5 9.0 8.5 8.0 7.5 7.0 6.5 6.0 5.5 5.0 4.5 4.0 3.5 3.0 2.5 2.0 1.5 1.0 0.5 0.0 -0.5
f1 (ppm)

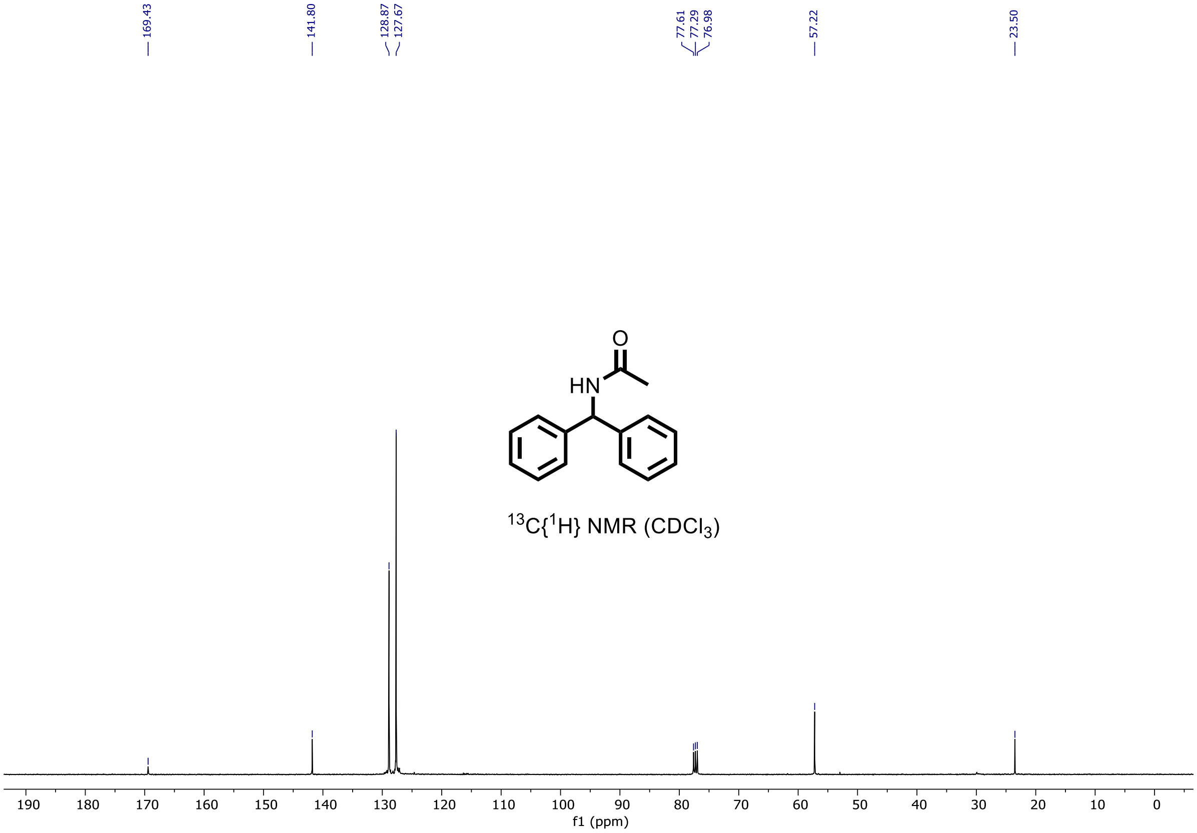
169.43
141.80
128.87
127.67
77.61
77.29
76.98
57.22
23.50
O
HN
13C{1H} NMR (CDCl3)
190 180 170 160 150 140 130 120 110 100 90 80 70 60 50 40 30 20 10 0
f1 (ppm)

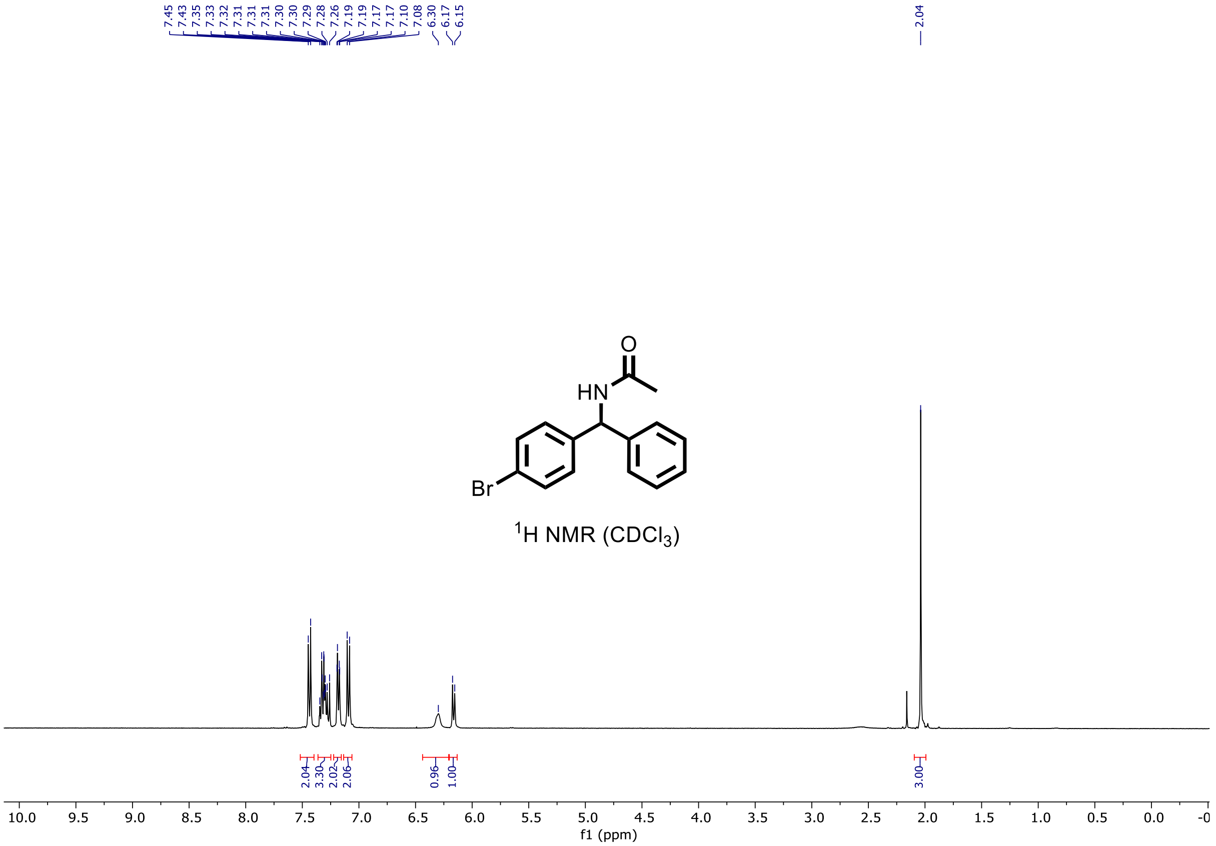
7.45
7.43
7.35
7.33
7.32
7.31
7.31
7.30
7.30
7.29
7.28
7.26
7.19
7.19
7.17
7.17
7.10
7.08
6.30
6.17
6.15
2.04
O
HN
Br
^{1}H NMR (CDCl$_3$)
2.04
3.30
2.02
2.06
0.96
1.00
3.00
f1 (ppm)

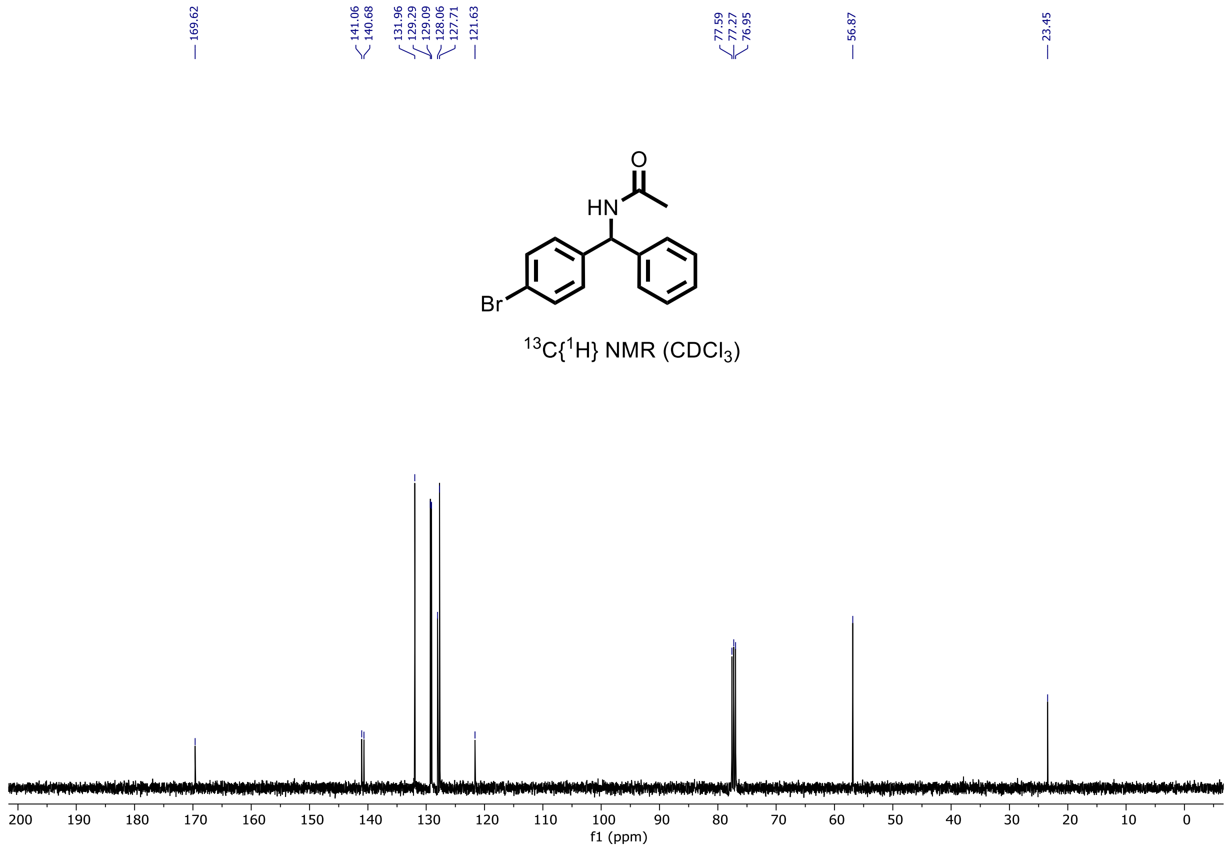
169.62
141.06
140.68
131.96
129.29
129.09
128.06
127.71
121.63
77.59
77.27
76.95
56.87
23.45
O
HN
Br
^{13}C{^{1}H} NMR (CDCl$_3$)
f1 (ppm)

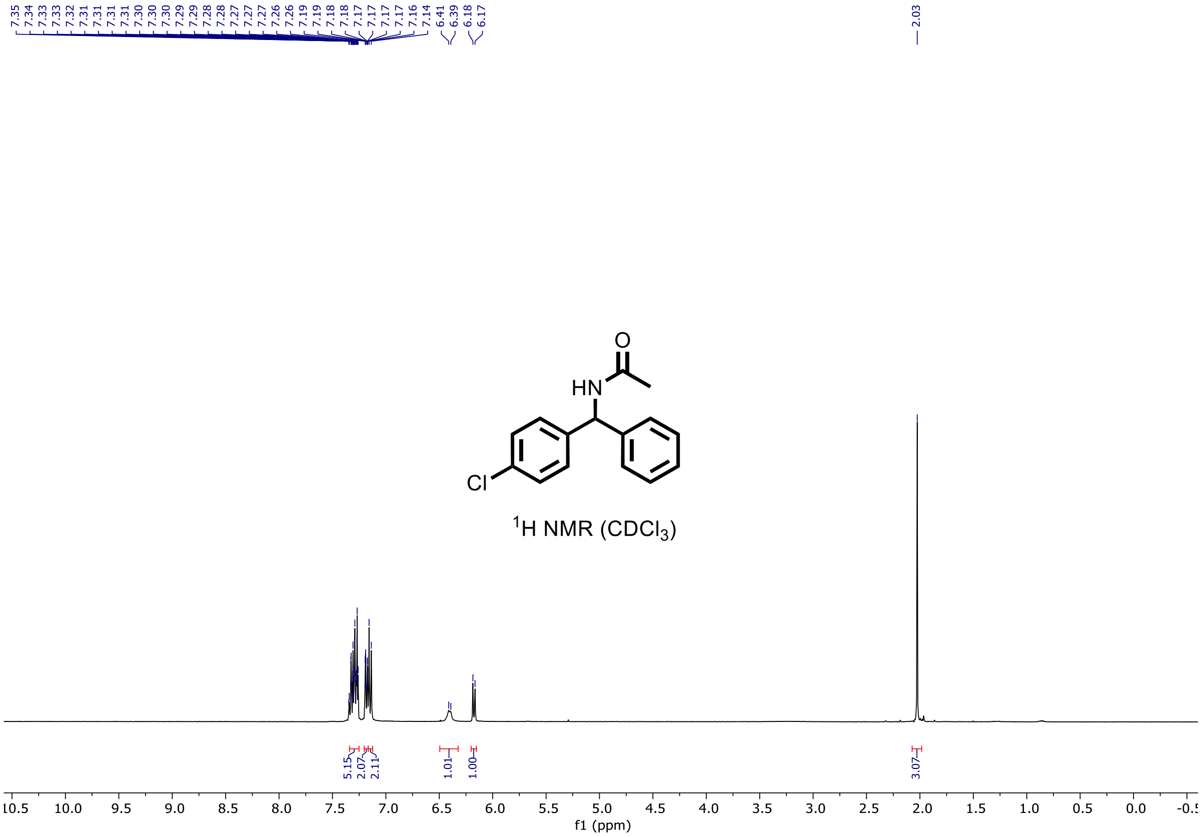

¹H NMR (CDCl₃)
f1 (ppm)

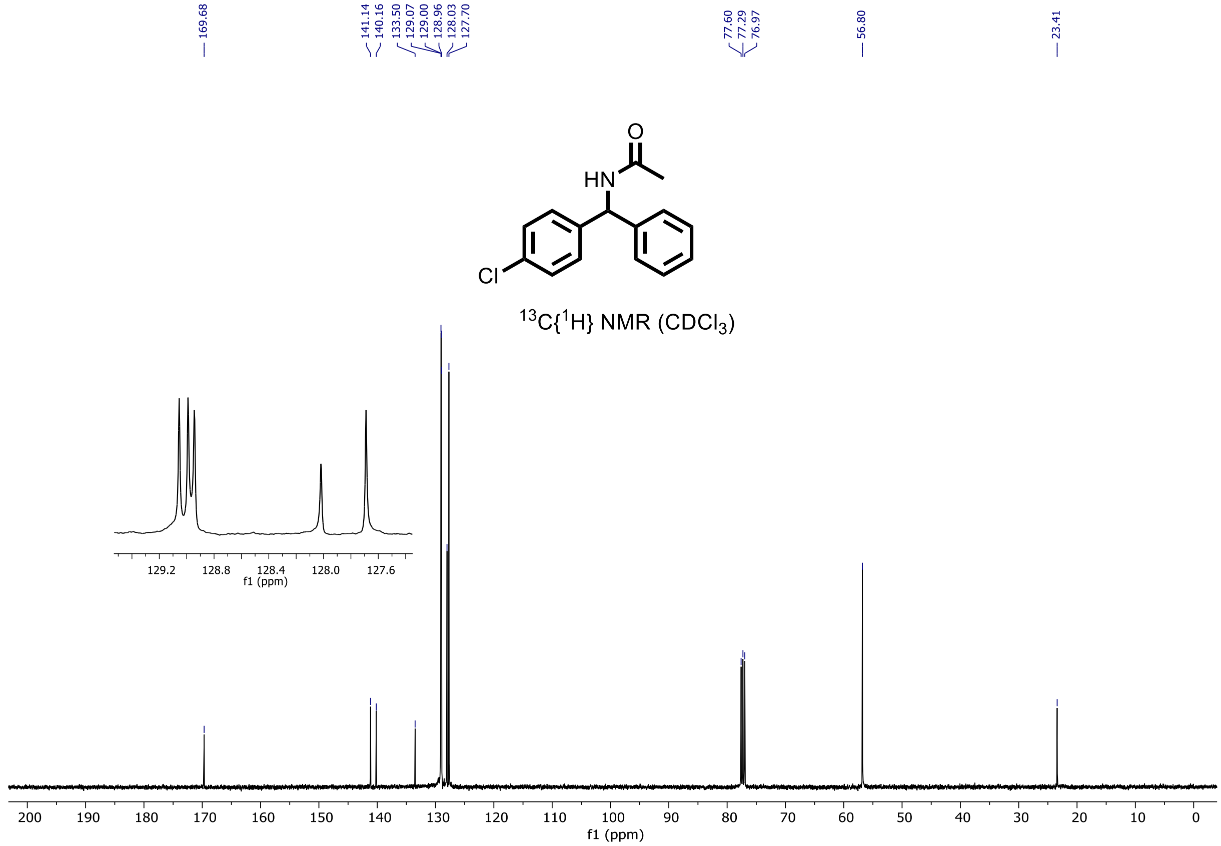

¹³C{¹H} NMR (CDCl₃)
f1 (ppm)

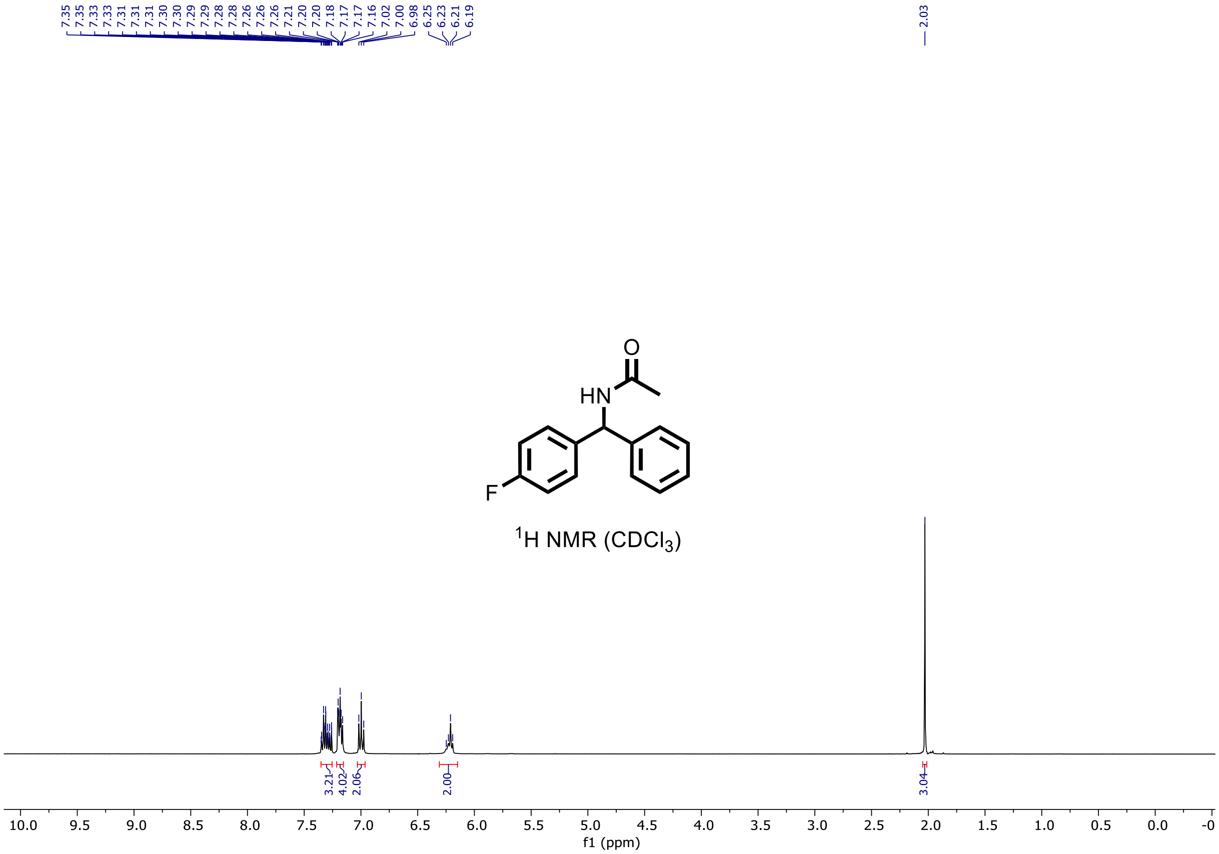

7.35
7.35
7.33
7.33
7.31
7.31
7.31
7.30
7.30
7.29
7.29
7.28
7.28
7.26
7.26
7.26
7.21
7.20
7.20
7.18
7.17
7.17
7.16
7.02
7.00
6.98
6.25
6.23
6.21
6.19
2.03
O
HN
F
^{1}H NMR (CDCl$_3$)
3.21
4.02
2.06
2.00
3.04
f1 (ppm)

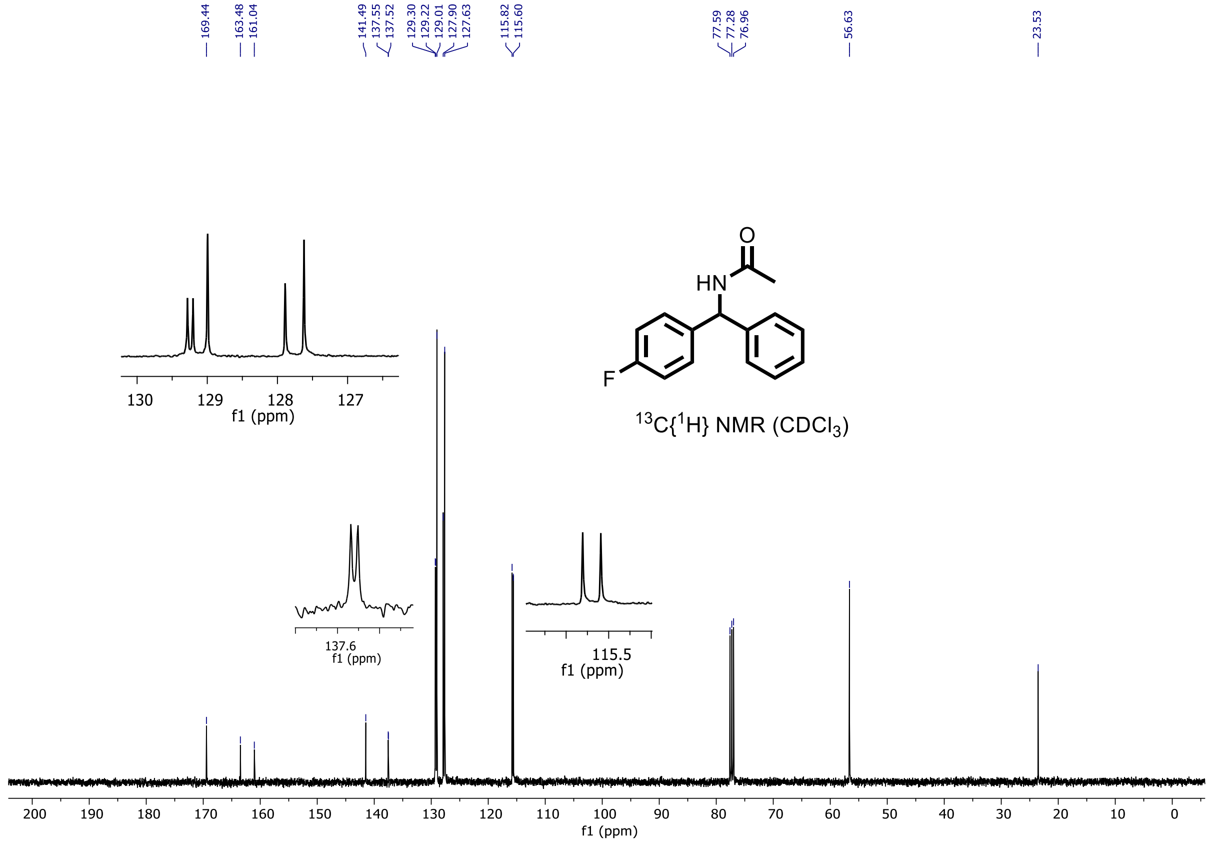

169.44
163.48
161.04
141.49
137.55
137.52
129.30
129.22
129.01
127.90
127.63
115.82
115.60
77.59
77.28
76.96
56.63
23.53
O
HN
F
^{13}C{^{1}H} NMR (CDCl$_3$)
f1 (ppm)

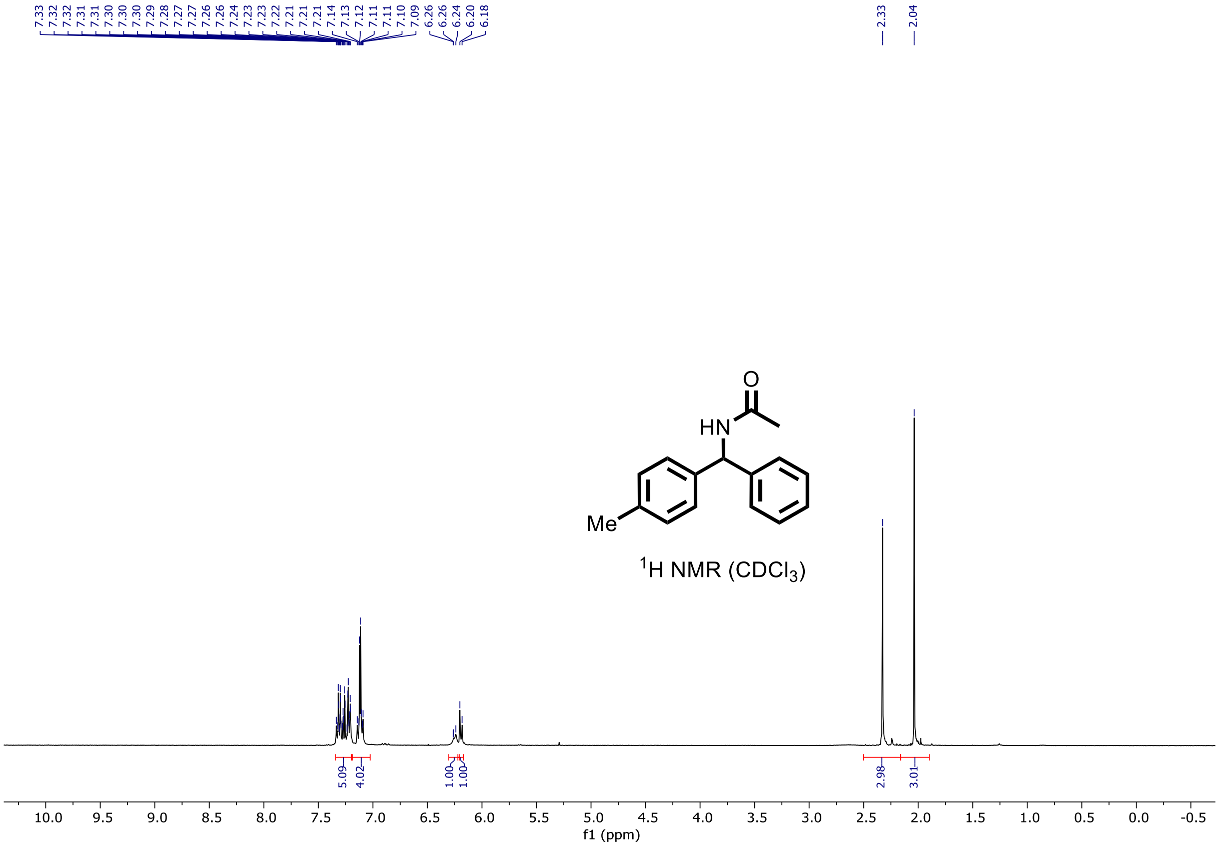
7.33 7.32 7.32 7.31 7.31 7.30 7.30 7.30 7.29 7.28 7.27 7.27 7.26 7.26 7.24 7.23 7.23 7.22 7.21 7.21 7.21 7.14 7.13 7.12 7.11 7.11 7.10 7.09 6.26 6.26 6.24 6.20 6.18
2.33
2.04
O
HN
Me
^{1}H NMR ($CDCl_3$)
5.09 4.02 1.00 1.00 2.98 3.01
10.0 9.5 9.0 8.5 8.0 7.5 7.0 6.5 6.0 5.5 5.0 4.5 4.0 3.5 3.0 2.5 2.0 1.5 1.0 0.5 0.0 -0.5
f1 (ppm)

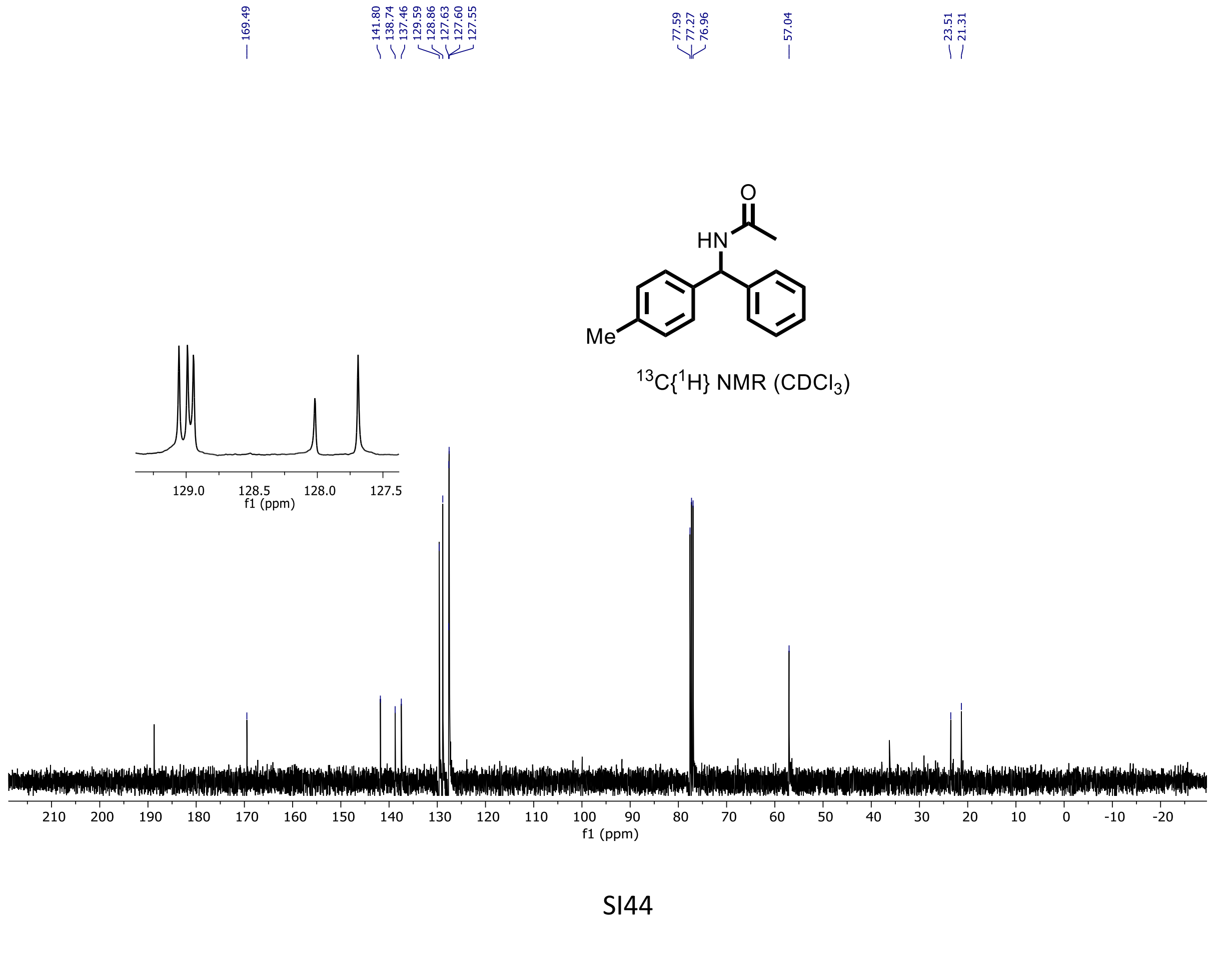
169.49
141.80 138.74 137.46 129.59 128.86 127.63 127.60 127.55
77.59 77.27 76.96
57.04
23.51 21.31
O
HN
Me
^{13}C{^{1}H} NMR ($CDCl_3$)
129.0 128.5 128.0 127.5
f1 (ppm)
210 200 190 180 170 160 150 140 130 120 110 100 90 80 70 60 50 40 30 20 10 0 -10 -20
f1 (ppm)

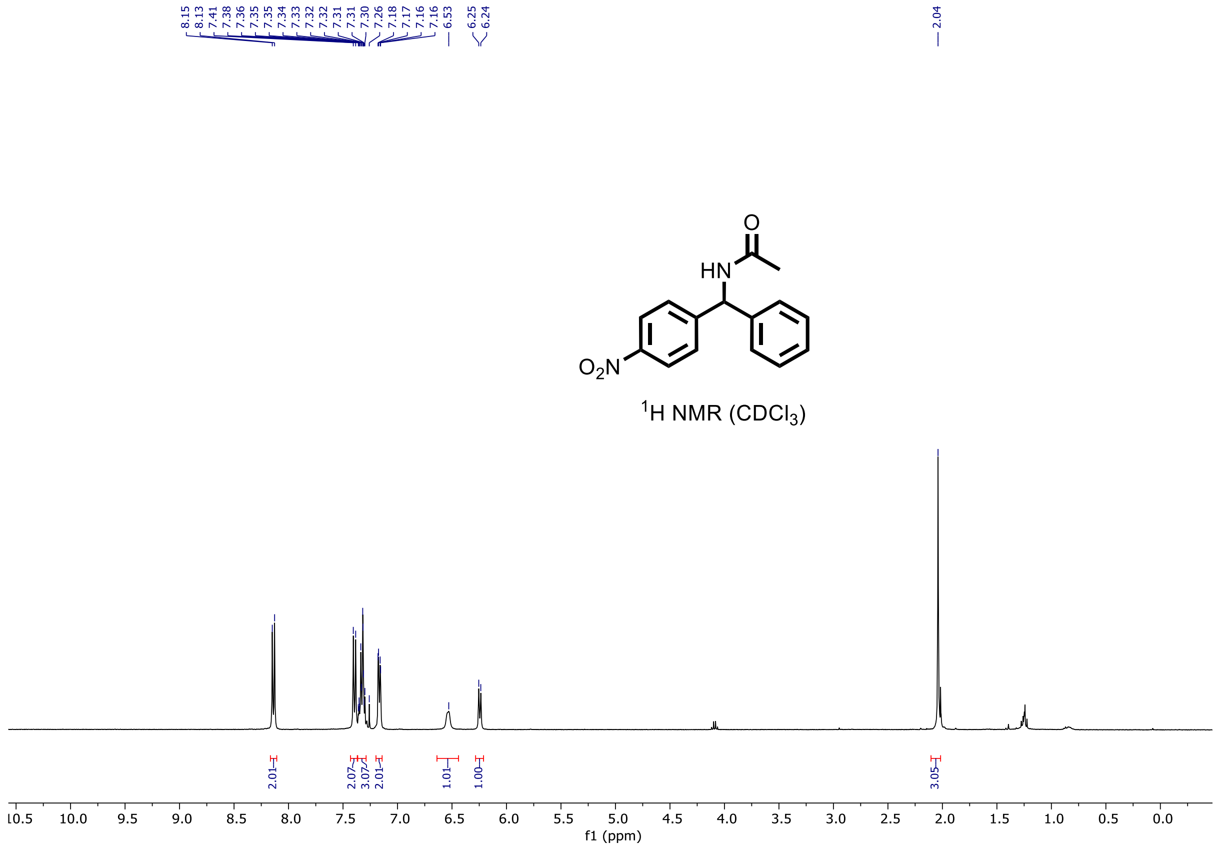
8.15
8.13
7.41
7.38
7.36
7.35
7.35
7.34
7.33
7.32
7.32
7.31
7.30
7.26
7.18
7.17
7.16
6.53
6.25
6.24
2.04
O
HN
O2N
1H NMR (CDCl3)
2.01
2.07
3.07
2.01
1.01
1.00
3.05
f1 (ppm)

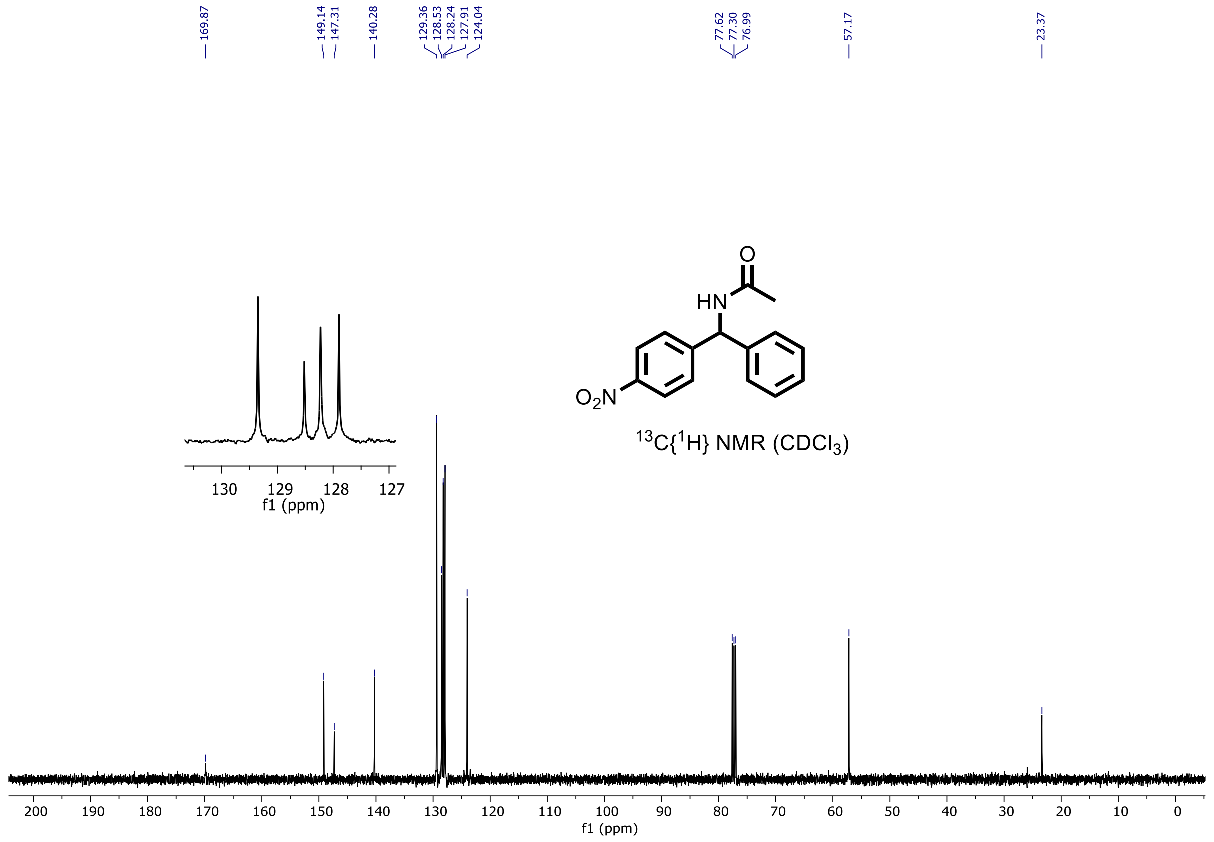
169.87
149.14
147.31
140.28
129.36
128.53
128.24
127.91
124.04
77.62
77.30
76.99
57.17
23.37
O
HN
O2N
13C{1H} NMR (CDCl3)
f1 (ppm)
f1 (ppm)

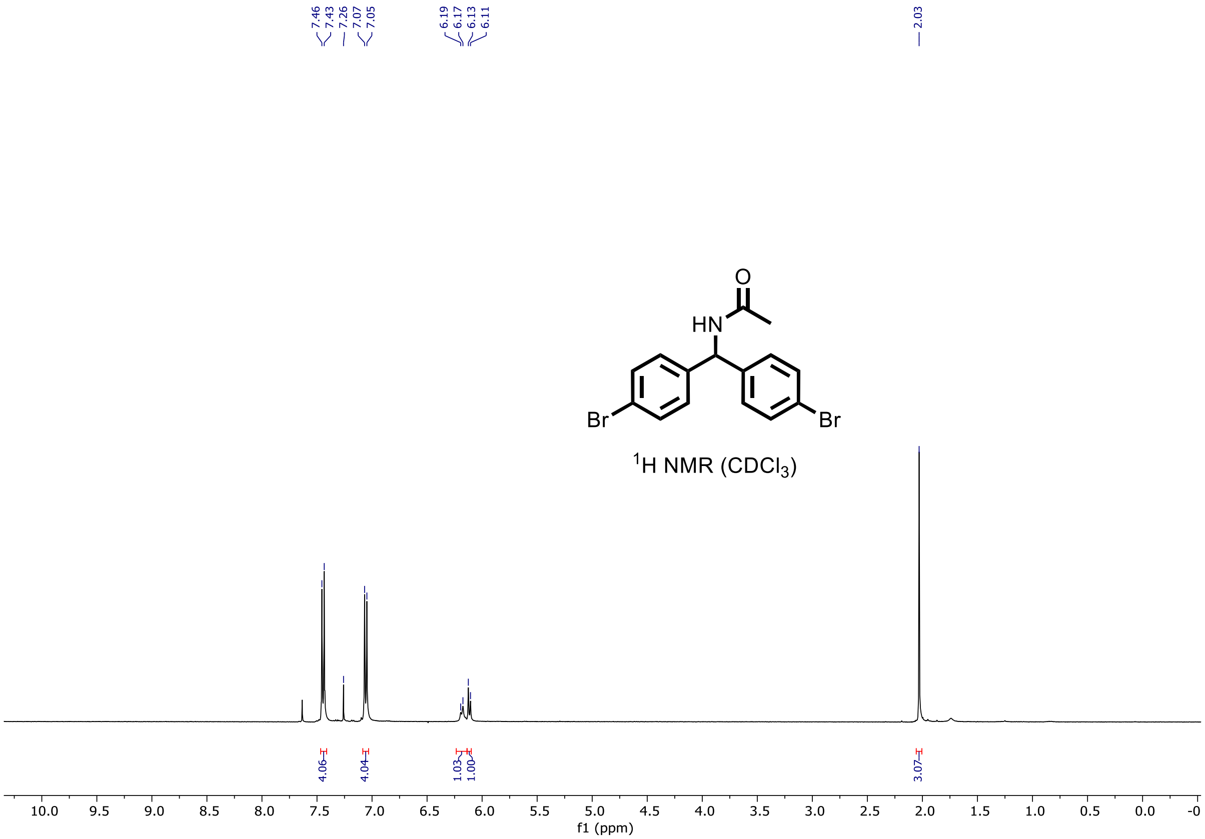
7.46
7.43
7.26
7.07
7.05
6.19
6.17
6.13
6.11
2.03
O
HN
Br
Br
1H NMR (CDCl3)
4.06
4.04
1.03
1.00
3.07
f1 (ppm)

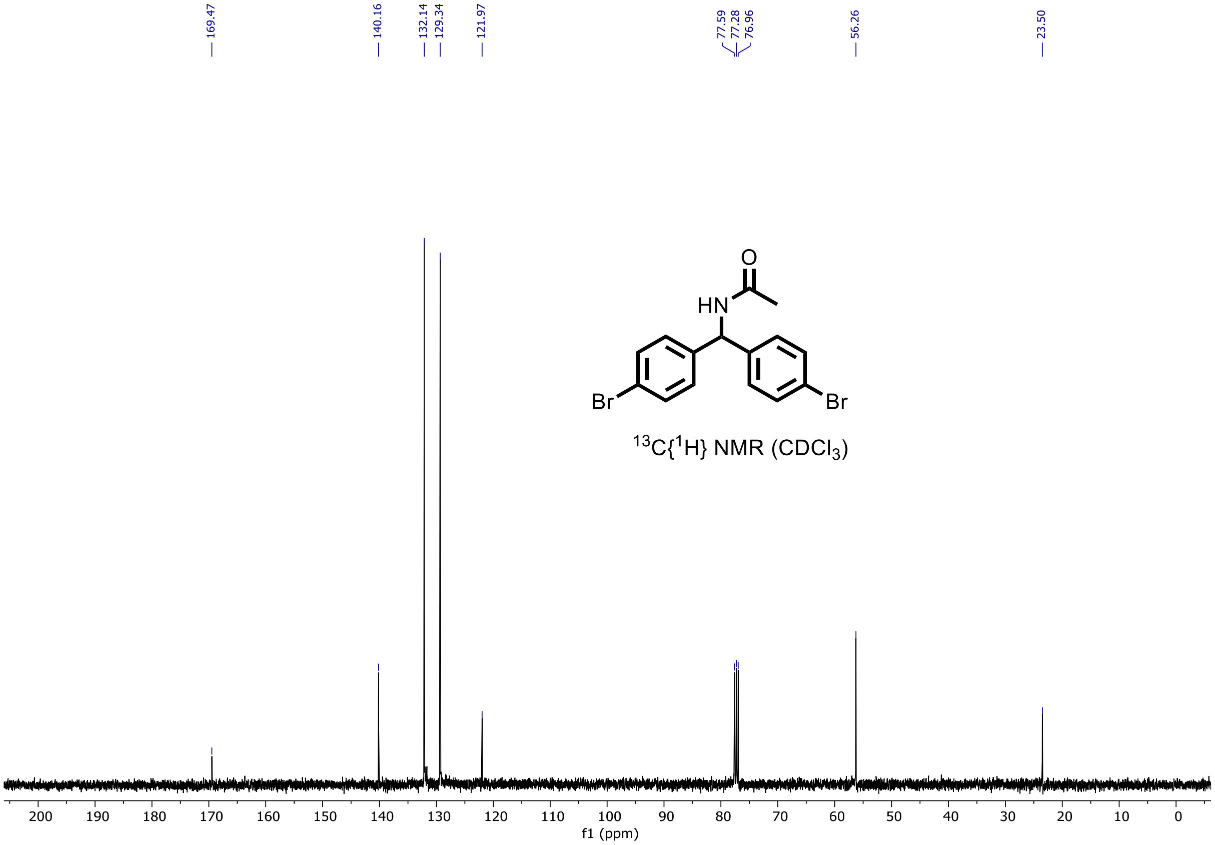
169.47
140.16
132.14
129.34
121.97
77.59
77.28
76.96
56.26
23.50
O
HN
Br
Br
13C{1H} NMR (CDCl3)
f1 (ppm)

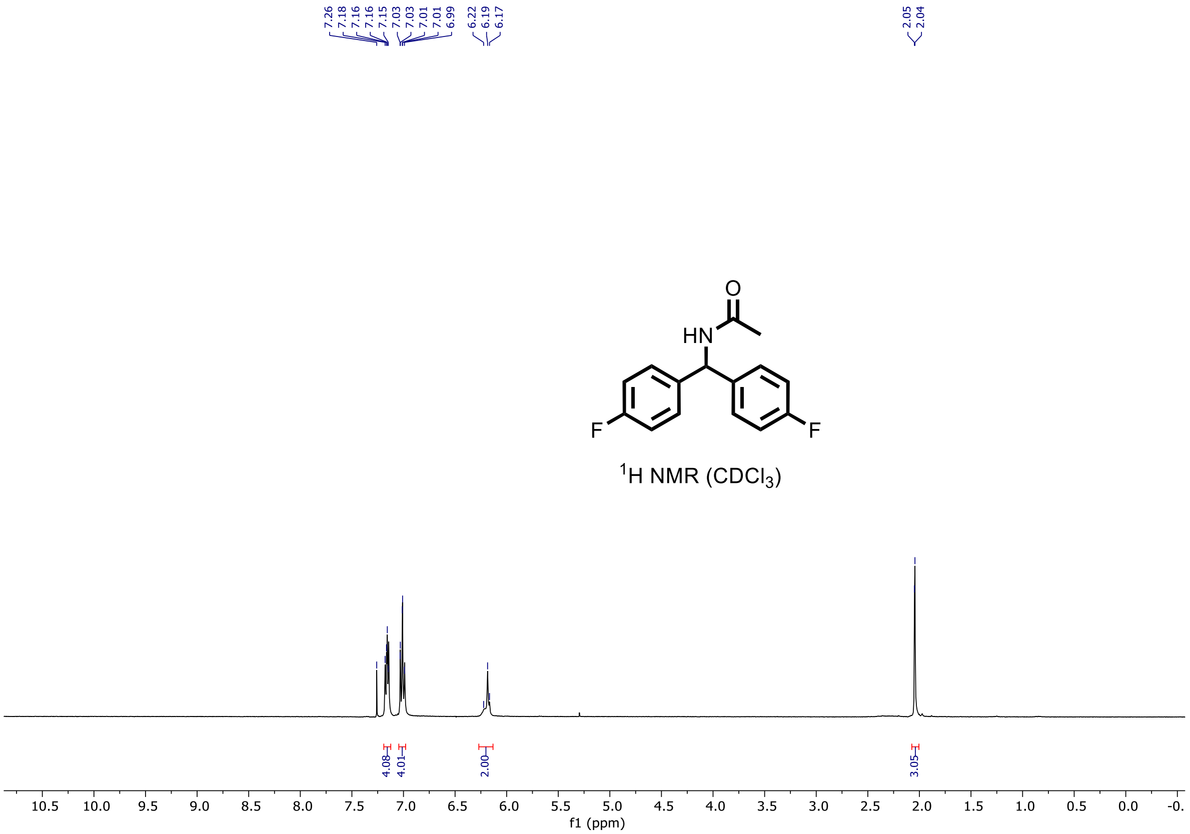
7.26 7.18 7.16 7.16 7.15 7.03 7.03 7.01 7.01 6.99 6.22 6.19 6.17
2.05 2.04
O
HN
F
F
^{1}H NMR (CDCl$_3$)
4.08 4.01 2.00 3.05
f1 (ppm)

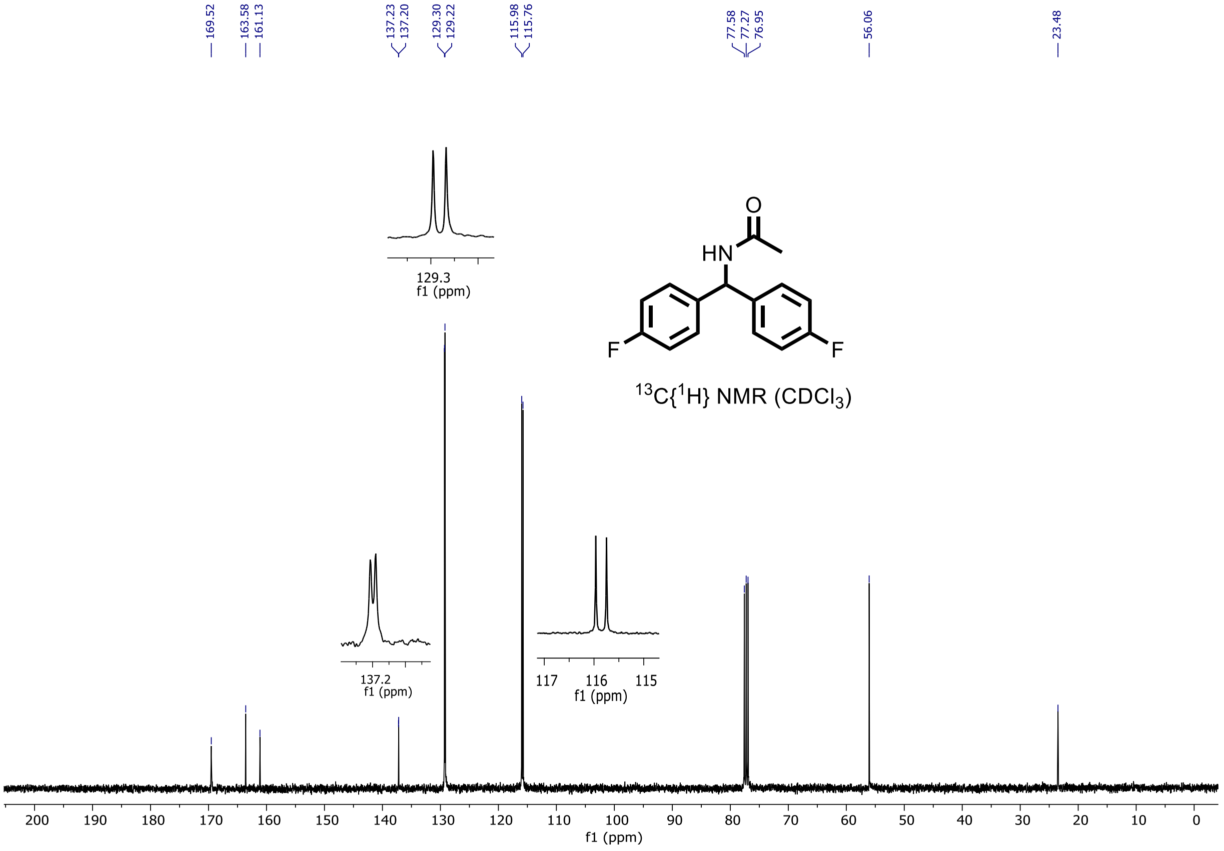
169.52 163.58 161.13 137.23 137.20 129.30 129.22 115.98 115.76 77.58 77.27 76.95 56.06 23.48
129.3
f1 (ppm)
O
HN
F
F
^{13}C{^{1}H} NMR (CDCl$_3$)
137.2
f1 (ppm)
117 116 115
f1 (ppm)
f1 (ppm)

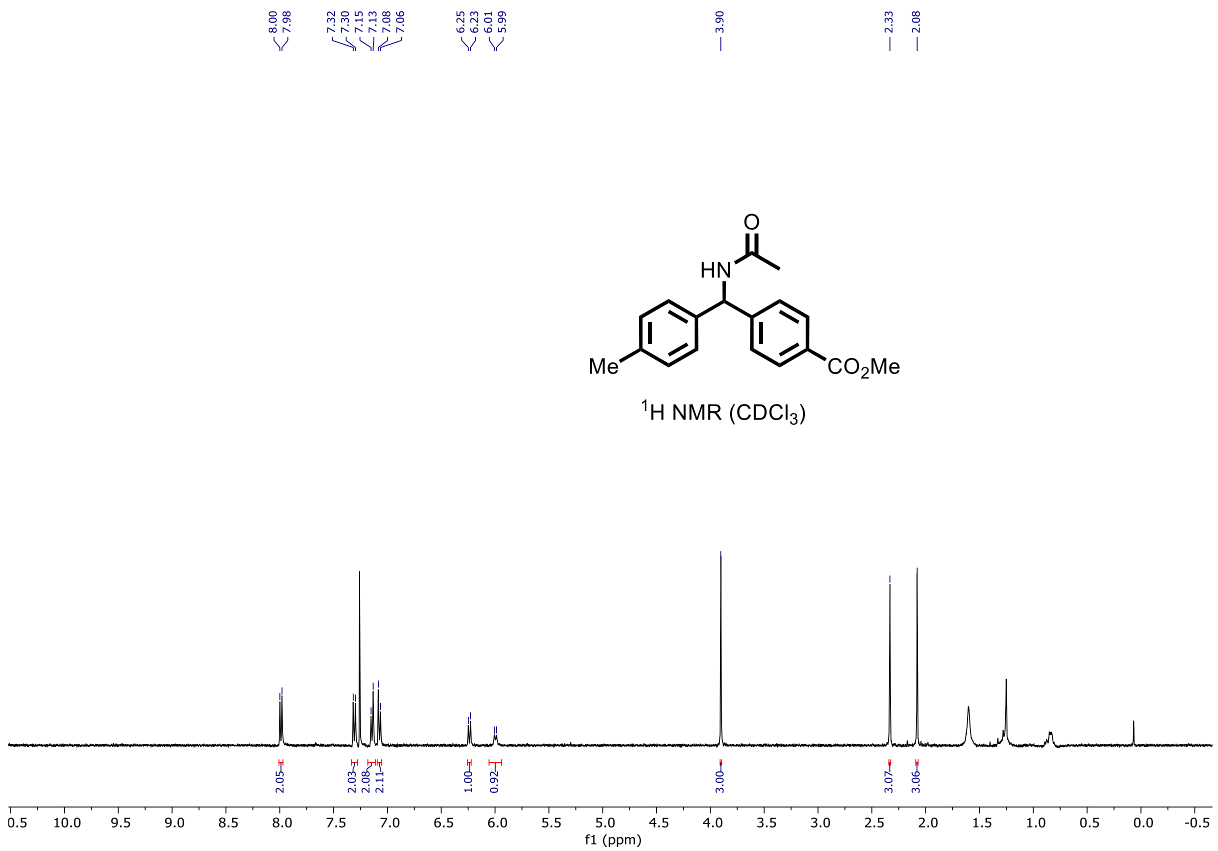
8.00
7.98
7.32
7.30
7.15
7.13
7.08
7.06
6.25
6.23
6.01
5.99
3.90
2.33
2.08
O
HN
Me
CO2Me
1H NMR (CDCl3)
2.05
2.03
2.08
2.11
1.00
0.92
3.00
3.07
3.06
f1 (ppm)

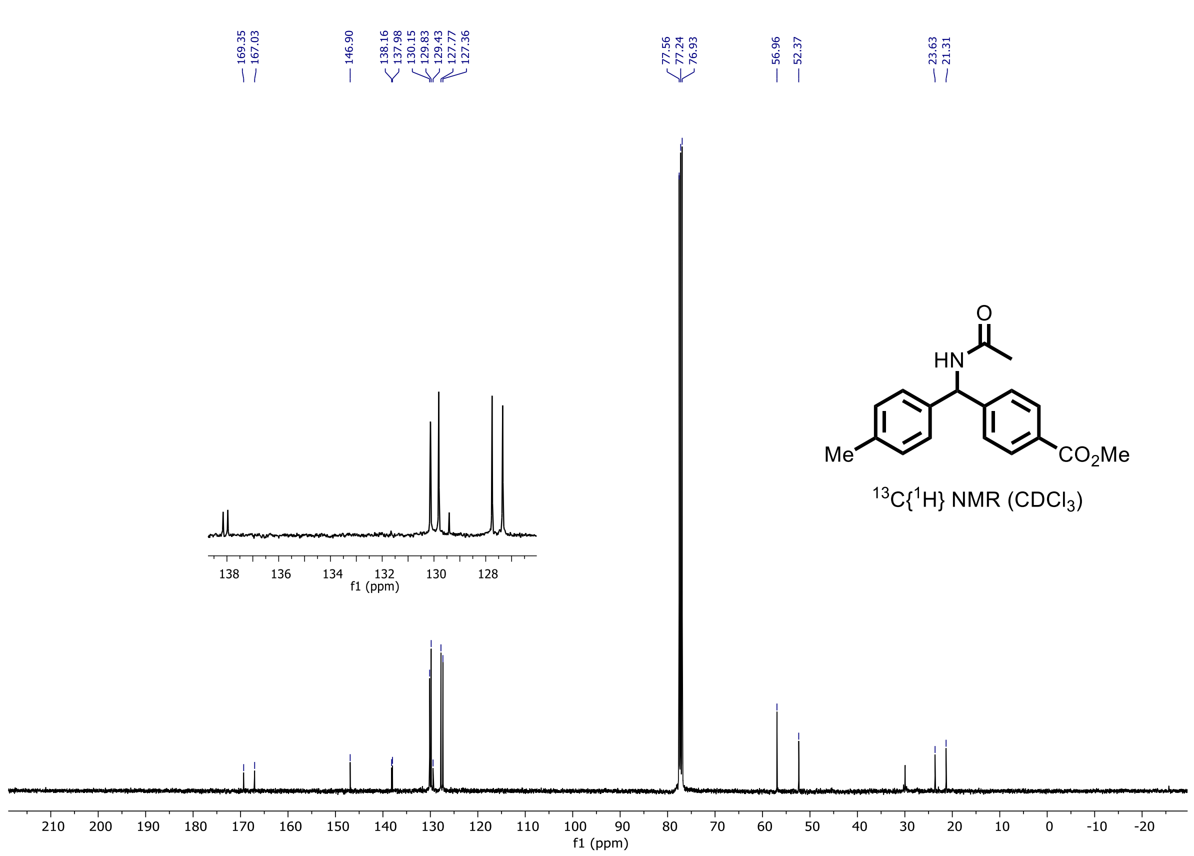
169.35
167.03
146.90
138.16
137.98
130.15
129.83
129.43
127.77
127.36
77.56
77.24
76.93
56.96
52.37
23.63
21.31
O
HN
Me
CO2Me
13C{1H} NMR (CDCl3)
f1 (ppm)
f1 (ppm)

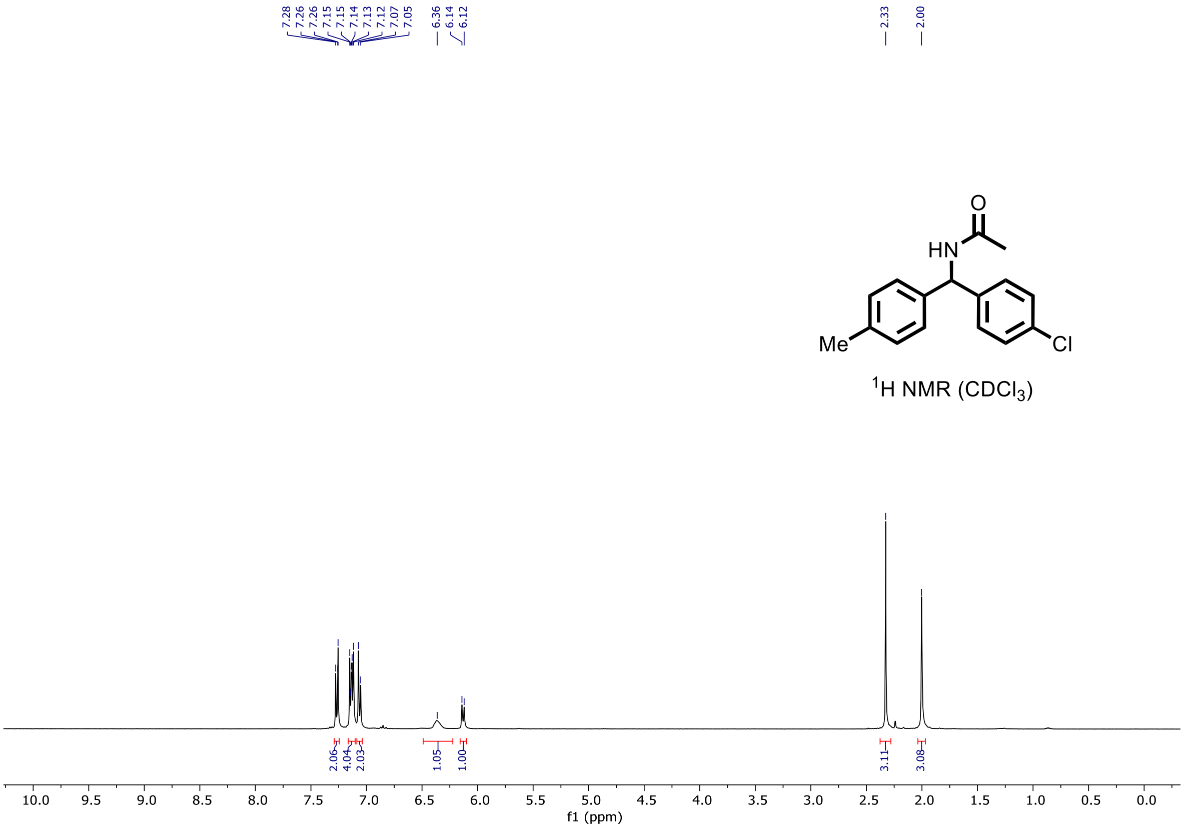
7.28
7.26
7.26
7.15
7.15
7.14
7.13
7.12
7.07
7.05
6.36
6.14
6.12
2.33
2.00
O
HN
Me
Cl
^{1}H NMR ($CDCl_3$)
2.06
4.04
2.03
1.05
1.00
3.11
3.08
f1 (ppm)

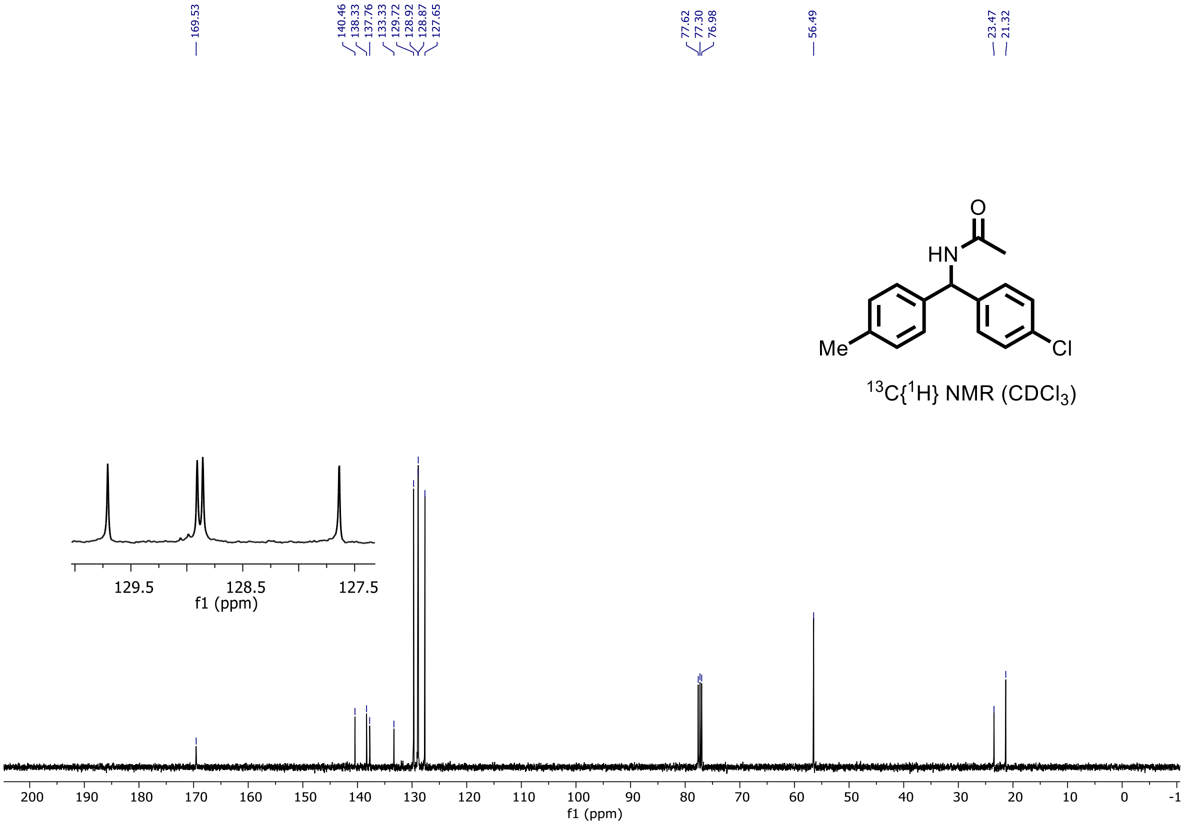
169.53
140.46
138.33
137.76
133.33
129.72
128.92
128.87
127.65
77.62
77.30
76.98
56.49
23.47
21.32
O
HN
Me
Cl
^{13}C{^{1}H} NMR ($CDCl_3$)
129.5
128.5
127.5
f1 (ppm)
f1 (ppm)

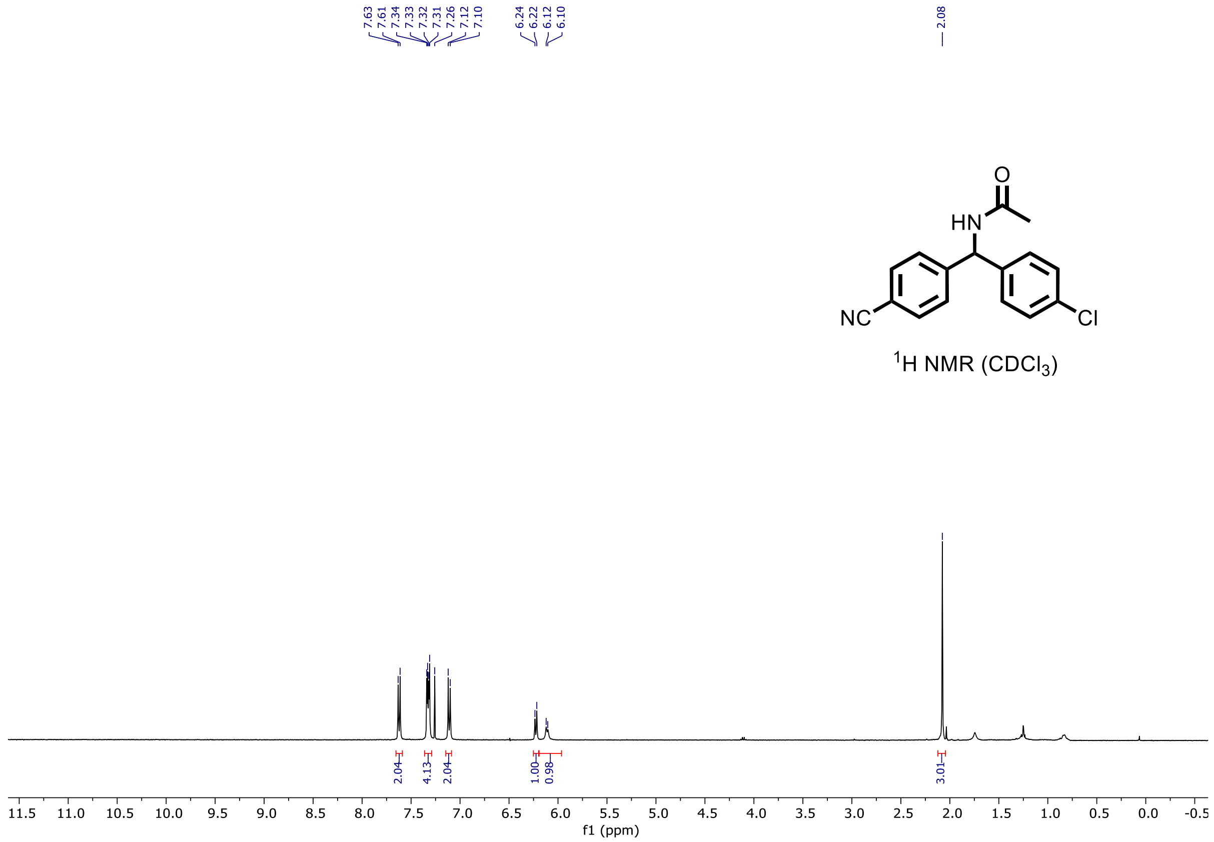
7.63
7.61
7.34
7.33
7.32
7.31
7.26
7.12
7.10
6.24
6.22
6.12
6.10
2.08
HN
O
NC
Cl
1H NMR (CDCl3)
2.04
4.13
2.04
1.00
0.98
3.01
f1 (ppm)

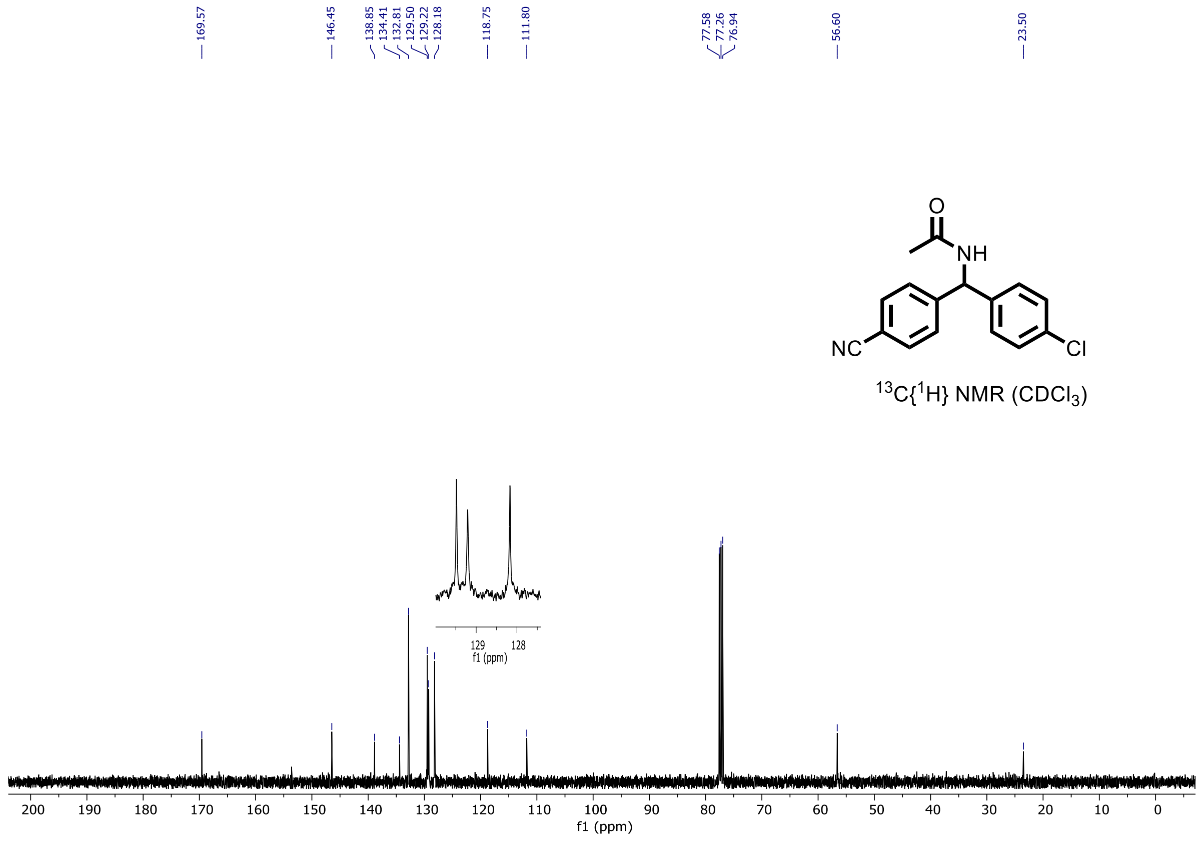
169.57
146.45
138.85
134.41
132.81
129.50
129.22
128.18
118.75
111.80
77.58
77.26
76.94
56.60
23.50
O
NH
NC
Cl
13C{1H} NMR (CDCl3)
129
128
f1 (ppm)
f1 (ppm)

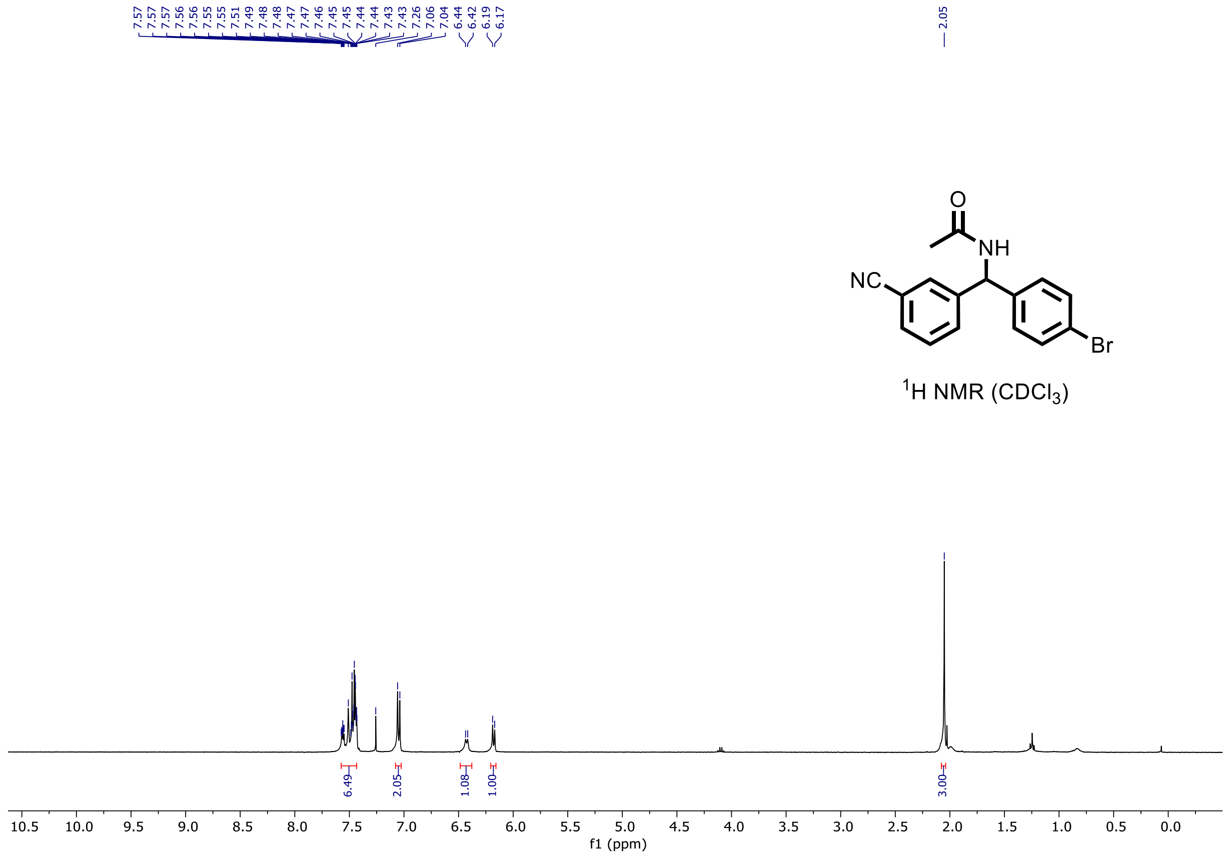
7.57
7.57
7.57
7.56
7.56
7.55
7.55
7.51
7.49
7.48
7.48
7.47
7.47
7.46
7.45
7.45
7.44
7.44
7.43
7.43
7.26
7.06
7.04
6.44
6.42
6.19
6.17
2.05
O
NH
NC
Br
1H NMR (CDCl3)
6.49
2.05
1.08
1.00
3.00
f1 (ppm)

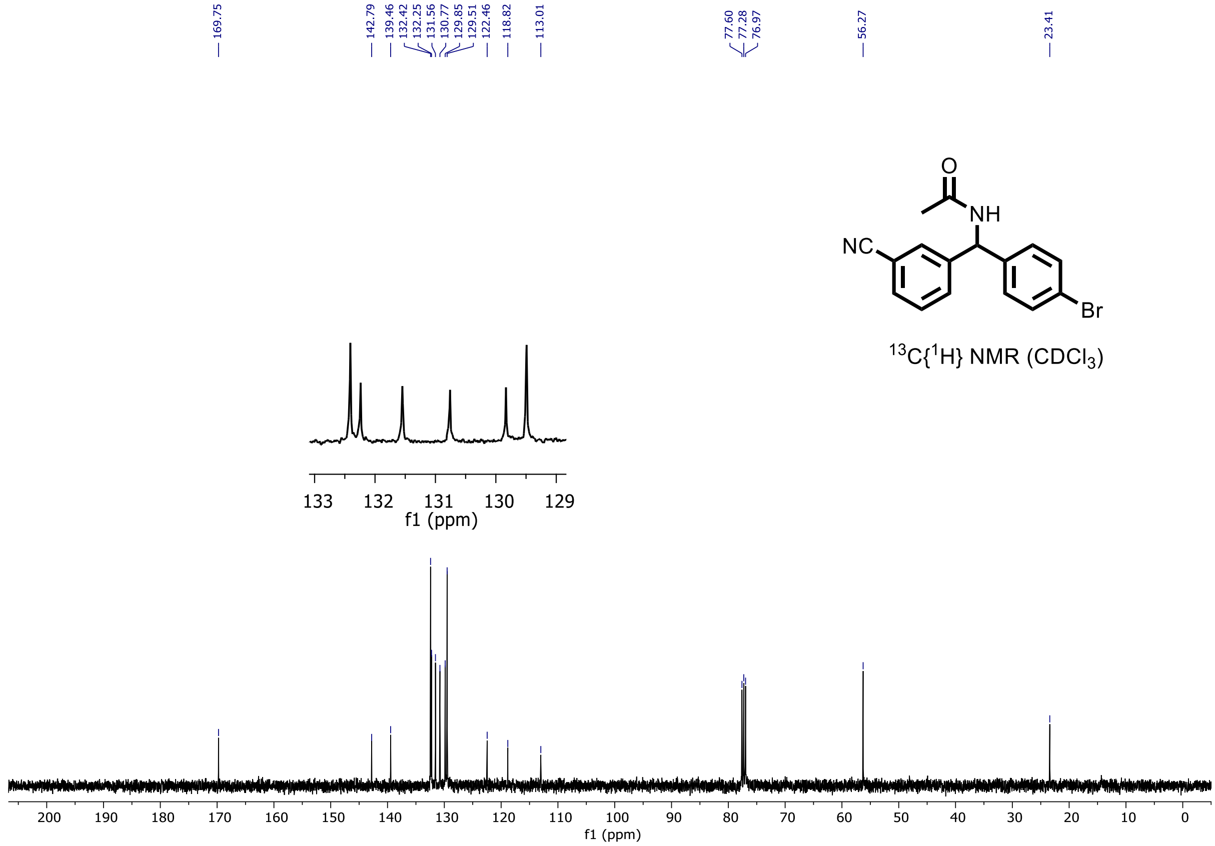
169.75
142.79
139.46
132.42
132.25
131.56
130.77
129.85
129.51
122.46
118.82
113.01
77.60
77.28
76.97
56.27
23.41
O
NH
NC
Br
13C{1H} NMR (CDCl3)
f1 (ppm)
f1 (ppm)

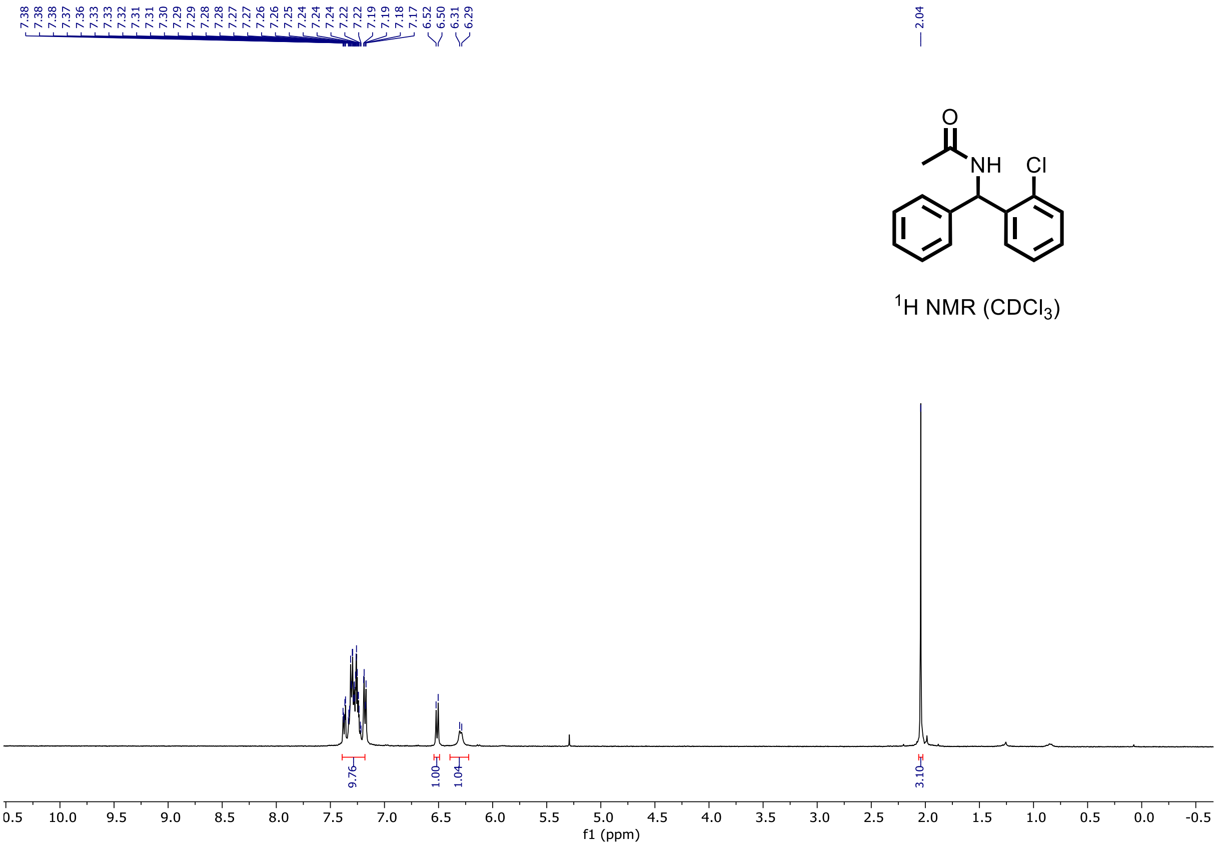
7.38 7.38 7.38 7.37 7.36 7.33 7.33 7.32 7.31 7.31 7.30 7.29 7.29 7.28 7.28 7.27 7.27 7.26 7.26 7.25 7.24 7.24 7.24 7.22 7.22 7.19 7.19 7.18 7.17 6.52 6.50 6.31 6.29
2.04
O
NH
Cl
^{1}H NMR (CDCl$_3$)
9.76
1.00
1.04
3.10
f1 (ppm)

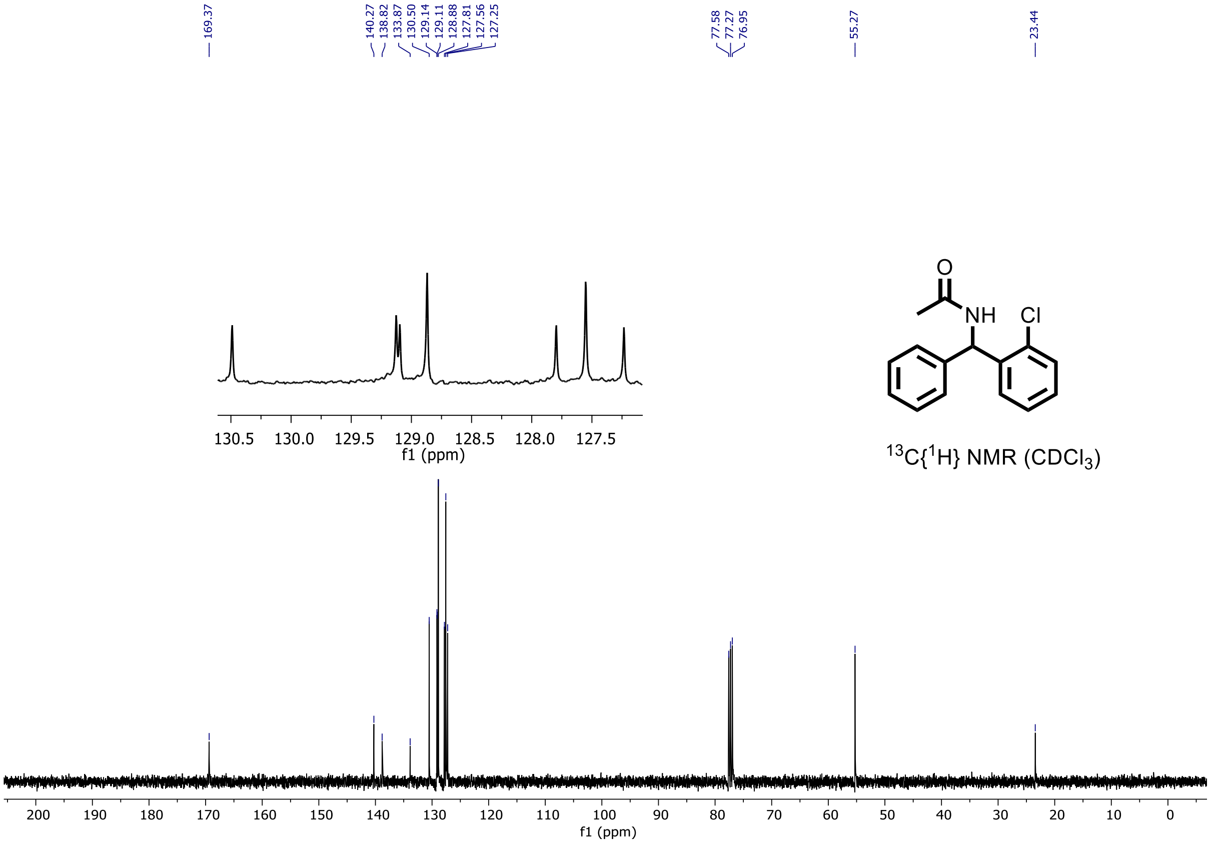
169.37
140.27 138.82 133.87 130.50 129.14 129.11 128.88 127.81 127.56 127.25
77.58 77.27 76.95
55.27
23.44
O
NH
Cl
^{13}C{^{1}H} NMR (CDCl$_3$)
f1 (ppm)

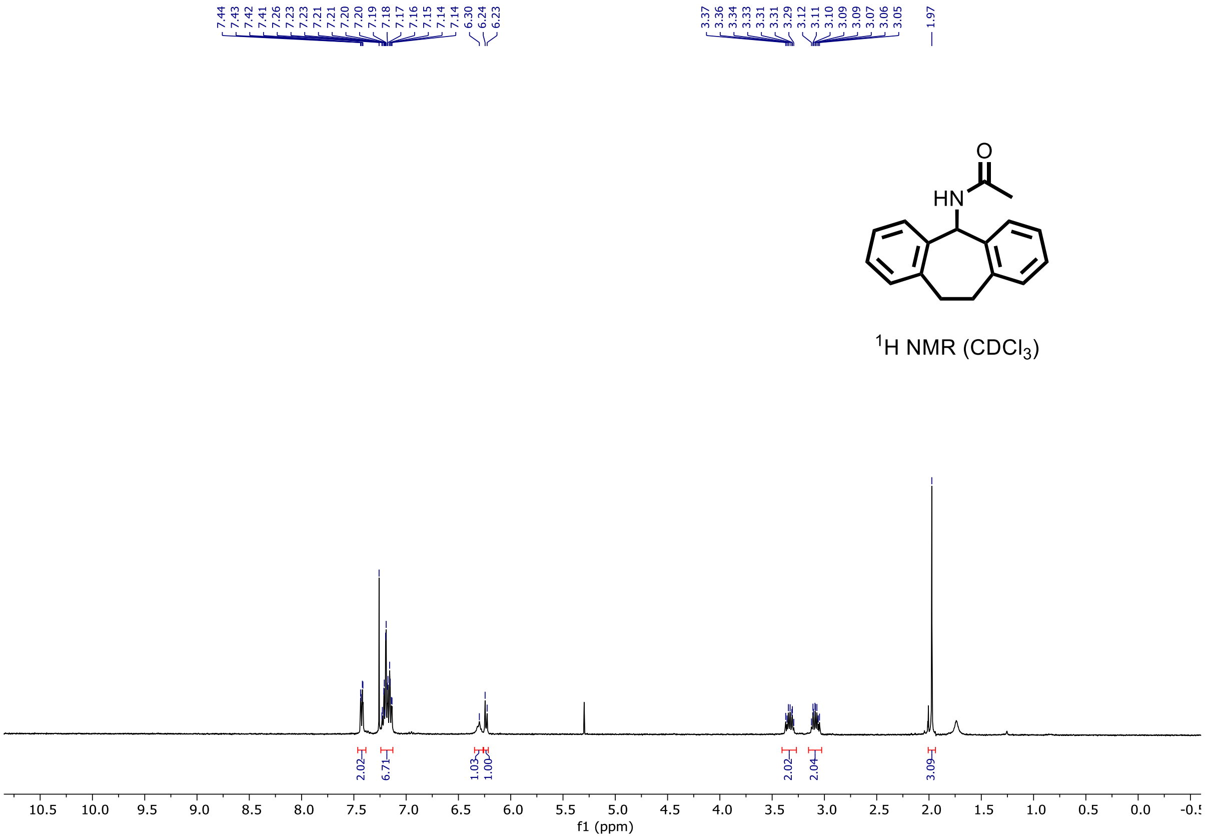

^{1}H NMR ($CDCl_3$)
7.44 7.43 7.42 7.41 7.26 7.23 7.23 7.21 7.21 7.20 7.19 7.18 7.17 7.16 7.15 7.14 7.14 6.30 6.24 6.23
3.37 3.36 3.34 3.33 3.31 3.31 3.29 3.12 3.11 3.10 3.09 3.09 3.07 3.06 3.05
1.97
2.02 6.71 1.03 1.00 2.02 2.04 3.09
f1 (ppm)

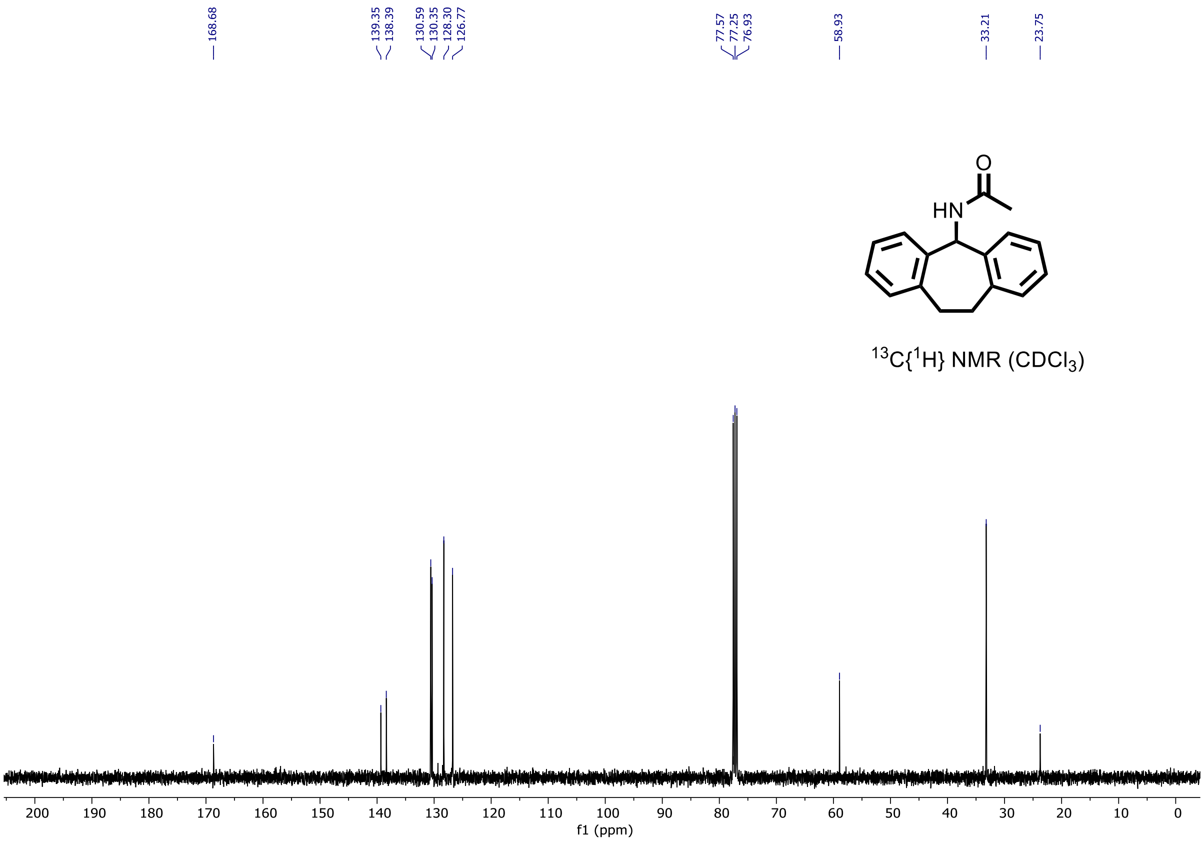

$^{13}C\{^1H\}$ NMR ($CDCl_3$)
168.68
139.35 138.39
130.59 130.35 128.30 126.77
77.57 77.25 76.93
58.93
33.21
23.75
f1 (ppm)
